# Genome-wide analyses of multiple obesity-related cytokines and hormones informs biology of cardiometabolic traits

**DOI:** 10.1186/s13073-021-00971-2

**Published:** 2021-10-07

**Authors:** Karlijn A. C. Meeks, Amy R. Bentley, Mateus H. Gouveia, Guanjie Chen, Jie Zhou, Lin Lei, Adebowale A. Adeyemo, Ayo P. Doumatey, Charles N. Rotimi

**Affiliations:** Center for Research on Genomics and Global Health, National Human Genome Research Institute, National Institutes of Health, 12 South Drive Bldg 12A rm 4047, Bethesda, MD 20814 USA

**Keywords:** Adipocytokines, Obesity, Cardiometabolic traits, GWAS, Africans

## Abstract

**Background:**

A complex set of perturbations occur in cytokines and hormones in the etiopathogenesis of obesity and related cardiometabolic conditions such as type 2 diabetes (T2D). Evidence for the genetic regulation of these cytokines and hormones is limited, particularly in African-ancestry populations. In order to improve our understanding of the biology of cardiometabolic traits, we investigated the genetic architecture of a large panel of obesity- related cytokines and hormones among Africans with replication analyses in African Americans.

**Methods:**

We performed genome-wide association studies (GWAS) in 4432 continental Africans, enrolled from Ghana, Kenya, and Nigeria as part of the Africa America Diabetes Mellitus (AADM) study, for 13 obesity-related cytokines and hormones, including adipsin, glucose-dependent insulinotropic peptide (GIP), glucagon-like peptide-1 (GLP-1), interleukin-1 receptor antagonist (IL1-RA), interleukin-6 (IL-6), interleukin-10 (IL-10), leptin, plasminogen activator inhibitor-1 (PAI-1), resistin, visfatin, insulin, glucagon, and ghrelin. Exact and local replication analyses were conducted in African Americans (*n* = 7990). The effects of sex, body mass index (BMI), and T2D on results were investigated through stratified analyses.

**Results:**

GWAS identified 39 significant (*P* value < 5 × 10^−8^) loci across all 13 traits. Notably, 14 loci were African-ancestry specific. In this first GWAS for adipsin and ghrelin, we detected 13 and 4 genome-wide significant loci respectively. Stratified analyses by sex, BMI, and T2D showed a strong effect of these variables on detected loci. Eight novel loci were successfully replicated: adipsin (3), GIP (1), GLP-1 (1), and insulin (3). Annotation of these loci revealed promising links between these adipocytokines and cardiometabolic outcomes as illustrated by rs201751833 for adipsin and blood pressure and locus rs759790 for insulin level and T2D in lean individuals.

**Conclusions:**

Our study identified genetic variants underlying variation in multiple adipocytokines, including the first loci for adipsin and ghrelin. We identified population differences in variants associated with adipocytokines and highlight the importance of stratification for discovery of loci. The high number of African-specific loci detected emphasizes the need for GWAS in African-ancestry populations, as these loci could not have been detected in other populations. Overall, our work contributes to the understanding of the biology linking adipocytokines to cardiometabolic traits.

**Supplementary Information:**

The online version contains supplementary material available at 10.1186/s13073-021-00971-2.

## Background

A complex system of cytokines and hormones is implicated in the pathogenesis of obesity and related cardiometabolic conditions, such as insulin resistance and type 2 diabetes (T2D) [1]. Cytokines are small proteins that can be divided into multiple subtypes depending on their site of production or function, with much functional overlap between these subtypes. One major subtype is adipocytokines, cytokines released by adipose tissue, such as adipsin, leptin, visfatin, plasminogen activator inhibitor-1 (PAI-1), and resistin. Interleukins (ILs) are cytokines that derive their name from their complex immunomodulatory functions in leukocytes. Several ILs including IL-6, IL-10, and IL-1 receptor antagonist (IL-1RA) have been implicated in insulin resistance [1]. Incretins, glucose-dependent insulinotropic peptide (GIP), and glucagon-like peptide-1 (GLP-1) are produced by the gut and influence the production of glucagon and insulin [2].

The role of these cytokines and hormones in the etiology of obesity and T2D is highly complex. Dysfunction of cytokines and hormones such as leptin, ghrelin, insulin, GLP-1, resistin, and visfatin has been implicated causally in the pathogenesis of obesity but also as a mediator between excess fat mass and insulin resistance, T2D, and cardiovascular disease [3–6]. There is a need for improved understanding of the biology of obesity-related cytokines and hormones because of the worldwide increase in obesity [7]. While absolute prevalence rates of obesity in sub-Saharan Africa are still relatively low, the burden of obesity is increasing rapidly, in particular among women residing in urban areas [8, 9]. Furthermore, African Americans (AA) and African migrants to the USA and Europe are disproportionally affected by obesity compared with European ancestry individuals in these environments [8, 10]. The limited evidence available indicates both ethnic [11] and sex differences [12, 13] in circulating levels of obesity-related cytokines and hormones. Leptin and IL-6 were found to be higher in AA compared with European Americans [11, 12, 14], while PAI-1 was found to be lower among South Africans with African ancestry compared with those of European ancestry [15]. Higher leptin and ghrelin levels were found in women compared with men [16, 17]. These ethnic and sex differences in circulating levels of obesity-related cytokines and hormones point to potential ancestry- and sex-specific effects on their biology.

Twin studies report that the circulating levels of obesity-related cytokines and hormones are highly heritable [18, 19]. This suggests that genetic factors play an important role in variation in circulating levels between individuals and between populations. Although several genome-wide association studies (GWAS) have reported genetic loci associated with circulating insulin levels [20–23], the number of GWAS on obesity-related cytokines and hormones other than insulin is limited and these GWAS have been conducted primarily in European ancestry populations [24–26]. Data on genetic loci involved in obesity and diabetes-related cytokines and hormones in African-ancestry populations are scarce [20, 27]. Furthermore, most previous GWAS report a single or a few traits at a time, despite their collective involvement in the etiology of metabolic disorders. Lastly, it has been suggested that the detection of novel loci for blood-based biomarkers is improved by performing sex-stratified analyses [28]. In this study, we aimed to study the genetic architecture of a panel of 13 obesity and diabetes -related cytokines and hormones. Specifically, we set out to (i) identify genetic loci for adipsin, ghrelin, and visfatin by conducting the first GWAS ever for these traits; (ii) compare genetic loci for 13 obesity and diabetes-related cytokines and hormones in African-ancestry populations by conducting the first GWAS for these traits in Africans; (iii) evaluate the effect of strata defined by sex, BMI, and T2D status on GWAS findings for the 13 studied obesity and diabetes-related cytokines and hormones; and (iv) explore the phenotypic and genetic correlation between these obesity-related cytokines and hormones.

## Methods

### Data and participants

#### Discovery cohort

We used data on 4432 sub-Saharan Africans from the Africa America Diabetes Mellitus (AADM) study for the discovery cohort. The AADM study is a cross-sectional study comprising data on individuals residing in sub-Saharan Africa and has been described in detail elsewhere [29–31]. Data were collected in three cities in Nigeria (Ibadan, Enugu, and Lagos), two cities in Ghana (Accra, and Kumasi), and in the city of Eldoret in Kenya. Ethical approval was obtained for each participating institution. All participants gave informed consent prior to enrollment in the study.

#### Replication cohorts

Replication analyses were performed using data on 7990 AA from five different cohorts: the Howard University Family Study (HUFS) [32], the Multi-Ethnic Study of Atherosclerosis (MESA) [33], the Cleveland Family Study (CFS) [34, 35], the Atherosclerosis Risk In Communities (ARIC) study [36, 37], and the Jackson Heart Study (JHS) [38]. The HUFS (Principal Investigator: CNR) was a study of AA from the Washington DC metropolitan area (USA) and was designed to investigate the genetic and environmental basis of common complex traits such as hypertension, obesity, and T2D [32]. The data for the other four cohorts were accessed through dbGaP (ARIC phs000280.v2.p1, phs000090.v2.p1; CFS phs000284.v1.p1; JHS phs000286.v4.p1, phs000499.v2.p1; and MESA phs000209.v13.p1, phs000420.v6.p3). The ARIC cohort aimed to study the risk factors for coronary heart disease and carotid atherosclerosis in four communities across the USA. We included 3039 AA participants of ARIC who were aged 45 to 64 years. The CFS is a family-based cohort designed to gain insight into sleep apnea. Individuals with a confirmed diagnosis of sleep apnea and at least two of their first-degree relatives were recruited from three area hospital sleep labs in the USA. Forty-six percent of the CFS sample was AA, and we included the 304 AA with cytokine data available. We included 1281 AA from the JHS, which aimed to investigate the causes of cardiovascular diseases in AA and recruited from rural and urban areas in Jackson, MS (USA). The aim of MESA was to identify factors of subclinical CVD that predict progression to clinically overt cardiovascular disease in a diverse sample. MESA included four ethnic groups (European American, Chinese American, AA, and Hispanic), from six US communities. Participants were aged 45–84 years and were free of clinical CVD at baseline. One thousand four hundred eighteen AA participants from MESA with cytokine data available were included in the replication effort. All cohorts obtained ethical approval from participating institutions and written informed consent from their participants prior to data collection.

#### Genotyping and quality control

Genotyping for the AADM study was performed using either the Affymetrix Axiom PANAFR SNP array or the Illumina’s Multi-Ethnic Global Array (MEGA) [31]. Quality control was performed for each of the arrays separately, resulting in a sample level genotype call rate of at least 0.95 for all samples. For the replication cohorts, genotyping was performed using the Affymetrix Genome-Wide Human SNP Array 6.0 [39]. For all cohorts, the SNP datasets were filtered for missingness per marker (> 0.05), minor allele frequency (< 0.01), and Hardy-Weinberg equilibrium (*P* value ≤ 1 × 10^−6^). Imputation for all cohorts was performed using the African Genome Resources Haplotype Reference Panel via the Sanger imputation Service [40]. Quality of imputation was evaluated using INFO scores and only SNPs with INFO scores > 0.3 were retained. After filtering, 18,199,418 variants remained in the final dataset for AADM and 18,093,757 variants for replication cohorts. We checked for population stratification using the “epacts-pca-plot” function in the EPACTS software package (version 3.2.6) [41] and identified three significant principal components (PCs) for AADM, one for HUFS, and two for the other replication cohorts [42].

#### Phenotyping

In all cohorts, demographic data such as age and sex were obtained through questionnaires. Height and weight were measured in light clothing and without shoes to the nearest 0.1 cm and kg. Body mass index (BMI) was calculated as weight/height^2^ (kg/m^2^). Type 2 diabetes (T2D) status was determined in all cohorts using the American Diabetes Association (ADA) criteria. This entailed a fasting plasma glucose cut-off of ≥ 7.0 mmol/L (126 mg/dL), or a 2-h post load value of ≥ 11.1 (mmol/L) on an oral glucose tolerance test (OGTT) on more than one occasion, or the reported use of glucose-lowering medication as prescribed by a physician confirmed by review of clinical records.

In the AADM study, the obesity and diabetes-related cytokines and hormones adipsin, GIP, GLP-1, leptin, PAI-1, resistin, visfatin, glucagon, and ghrelin were measured on fasting serum samples using multiplex bead-based flow cytometric immunoassays—containing dyed microspheres linked with monoclonal antibodies specific for each protein plex—according to the manufacturer’s instructions (Bio-Plex Pro human diabetes: 10-plex, Cat#171A7001M and 2-plex, Cat#171A7002M, Bio-Rad, Inc., Hercules, CA, USA). These commercial kits measure the levels of cytokines and hormones involved in human obesity and diabetes. Data were collected using Bio-Plex 200®System (Luminex Corporation, Austin, TX) equipped with Bio-Plex Manager™ Software (Bio-Rad, Inc., Hercules, CA, USA). IL-1RA, IL-6, and IL-10 were analyzed using Enzyme-Linked Immuno Sorbent Assay (ELISA) (Quantikine ELISA, R&D Systems, Minneapolis, MN, USA). Insulin was measured by electrochemiluminescence immunoassay (ECLIA) on Roche Modular-E or Elecsys 2010 analyzers (Roche Diagnostics, Indianapolis, IN). The obesity and diabetes-related cytokines and hormones available in the AA replication cohorts differed by cohort. Ten cytokines and hormones were only available in the HUFS dataset (adipsin, ghrelin, leptin, GLP-1, GIP, resistin, glucagon, IL-10, IL-1RA and visfatin) (Additional File 1: Table S1). For these cytokines and hormones, the same measurement assays were used as in the AADM study. PAI-1, insulin, and IL-6 were evaluated in a combination of the AA cohorts, including HUFS for insulin and IL-6 (Additional File 1: Table S1). PAI-1 was evaluated in MESA, CFS, and ARIC (*n* = 594). PAI-1 was measured by a two-site sandwich ELISA in all three cohorts [43]. Insulin was available in MESA, JHS, ARIC, and HUFS (*n* = 7645). Insulin was measured via a radioimmunoassay method using the Linco Human Insulin Specific RIA kit (Linco Research) in the MESA and in the ARIC study, and using the Vitros 950 or 250, Ortho-Clinical Diagnostics analyzer (Raritan, NJ) in the JHS. IL-6 was available in MESA, CFS, and HUFS (*n* = 2517) and measured using ELISA in all three cohorts (R&D systems, Minneapolis, MN, USA).

### Statistical analyses

#### Discovery

Heritability of all 13 obesity and diabetes-related cytokines and hormones was calculated using the Genome-wide Complex Trait Analysis – Genomic-Relatedness-based restricted Maximum-Likelihood (GCTA-GREML) approach [44] on the inverse normal transformed traits. For these heritability analyses, genotyping data were pruned using *PLINK* 1.9 [45]. The *GCTA* software was used to calculate a genetic relationship matrix (GRM) and based on this GRM we filtered for relatedness > 0.2 as the GCTA-GREML method assumes unrelated individuals, which removed 1382 individuals. Age, sex, T2D, and the first three PCs were included as covariates in heritability analyses. To perform genome-wide quantitative linear regression analyses on each of the traits, we used the *EPACTS* software package (version 3.2.6) [41]. Estimated allele dosages were used rather than hard genotype calls in all GWAS analyses. This was done to take the imputation quality into account and thereby adjusting for uncertainty in genotype prediction. All cytokines and hormones were transformed to normality using inverse normal transformations. The base model for each cytokine and hormone included adjustment for age, sex, T2D, the first three PCs, and the GRM calculated in *EPACTS*. Inclusion of the genotyping array as a covariate in our analysis models was evaluated but array was not included in final models as this adjustment did not alter findings. Bayesian colocalization analyses were performed on GWAS summary statistics with expression Quantitative Trait Loci (eQTL) data using the *coloc* package in R version 4.0.5 [46, 47]. Expression data for 49 tissues were obtained from the Genotype-Tissue Expression (GTEx) Portal V8 [48]. Colocalization analyses were restricted to a 200-kb window around the base model lead variants. A posterior probability of hypothesis 4 ≥ 0.9 or > 0.5 was considered as having strong or moderate evidence for colocalization, respectively. Next, GWAS models were run in stratified analyses: men and women, lean (BMI < 25 kg/m^2^) and overweight (including obese) individuals (BMI ≥ 25 kg/m^2^), and T2D controls and T2D cases. A *P* value of < 5 × 10^−8^ was considered genome-wide significant in all discovery analyses. Loci passing a more stringent threshold taking into account the multiple traits and the stratification have been annotated in the tables (*P* value < 1.32 × 10^−9^ = 5 × 10^−8^/(12 traits × 3 strata + 1 trait [insulin] × 2 strata). The sex, BMI, and T2D strata were each meta-analyzed in METAL [49] to obtain heterogeneity *P* values for all variants identified in stratified analyses. Variants identified, in either the base (non-stratified) model or the stratified models, that are observed in African populations but are not observed in populations without African ancestry in the Allele Frequency Aggregator (ALFA) database [50] were considered African-ancestry specific. Phenotypic correlation between the included cytokines and hormones was studied using partial correlation including age, sex, and T2D as covariates and subsequently by PC factor analysis in STATA version 15.1 (StataCorp, Texas) [51] on the inverse normal transformed traits. For the factor analyses, IL1-RA, IL-6, and IL-10 were excluded because of their relatively low sample size. Only factors with eigenvalue > 1 were retained. Participants with T2D (*n* = 2082) were excluded from all fasting insulin analyses. Lastly, the list of genes from the base model was submitted to *Ingenuity Pathway Analysis* (*IPA*) (QIAGEN Inc., www.qiagen.com/ingenuity) for canonical pathway analyses through the use of the core analysis feature of *IPA* [52]. IPA calculates *P* values of overlap, using the right-tailed Fisher’s exact test, where we considered a *P* value of < 0.05 as statistically significant.

#### Replication

The same association analysis software (*EPACTS*), models, and transformations were used to perform genome-wide quantitative linear regression analyses in each of the replication cohorts. For HUFS, the first PC was included as a covariate in all models and the first two PCs were included for the other replication cohorts, based on assessment of residual population stratification, which was low after adjusting for the respective PCs. For PAI-1, insulin, and IL-6, subsequent fixed-effects meta-analyses were conducted with the *METAL* software [49] using the classical approach which combines effect size estimates and standard errors to combine the multiple cohorts. Heterogeneity *P* values were also calculated using *METAL*. To harmonize, the first two PCs were included in all models and cohorts that were merged in meta-analyses. Either HUFS linear regression results (adipsin, ghrelin, GIP, GLP-1, glucagon, IL-1RA, IL-10, resistin, visfatin) or combined meta-analyses results (IL-6, insulin, PAI-1) were subsequently used for follow-up analyses. First, we performed exact replication, where we evaluated per trait and per model the genome-wide significant loci in AADM for significance (*P* value < 0.05) in the replication cohorts. Secondly, local replication was performed based on a linkage disequilibrium (LD) block of 500 kb around the AADM significant variants, retaining only those variants with a *r*^*2*^ of > 0.3. To determine statistical significance in the local replication, *P* value thresholds were adjusted for the effective number of variants (the effective degrees of freedom, *N*_eff_) in the LD block as described by Ramos et al. [53]. *N*_eff_ was estimated by spectrally decomposing the covariance matrix and then using the following formula: *N*_eff_ = (∑*k* = 1*Kλk*)^2^/(∑*k* = 1*Kλ*2*k*)^2^, in which *λ*_k_ is the *k*th eigenvalue of the *K* × *K* covariance matrix for the *K* SNPs. The nominal significance threshold *α* = 0.05 was subsequently divided by *N*_eff_ to obtain the adjusted *P* value thresholds. In both exact and local replication, only variants below the *P* value thresholds that had the same direction of effect as in the discovery cohort were considered replications. In addition, in silico replication was performed by lookup in the GWAS catalog (https://www.ebi.ac.uk/gwas/) [54]. A 500 kb region around each detected variant was extracted from the GWAS catalog to determine if loci were novel (> 250 kb from a known locus) or known (≤ 250 kb of a known locus).

## Results

### Characteristics of the study populations

The discovery cohort included 4432 continental Africans (AF) enrolled from Nigeria, Ghana, and Kenya as part of the Africa America Diabetes Mellitus (AADM) study who had at least one obesity and diabetes-related cytokine or hormone measure available (Table 1). Out of the AF samples included, the majority were from Yoruba (27.1%), Ibo (21.5%), or Akan (18.6%) ethnolinguistic groups. Results from PC analyses showed that the first PC separated East from West Africa and the second PC was a gradient across West Africa (Additional File 2: Fig S1a). As expected, these AF samples clustered with African-ancestry populations of the 1000 Genomes Project (Additional File 2: Fig S1b). The replication cohorts combined included 7990 AA with at least one obesity and diabetes-related cytokine or hormone measure available (Additional File 1: Table S1). For the AA samples included, the average proportion of West African ancestry was 80.0% for HUFS, 82.0% for ARIC, 79.2% for CFS, 82.2% for JHS, and 77.7% for MESA as derived using admixture analysis [55]. The mean age was lowest in HUFS (39.4 years) and highest for MESA (62.1 years). Over half of participants were women in all cohorts (Table 1). The mean BMI was substantially lower in AF (26.5 kg/m^2^) compared with AA (ranging from 29.7 kg/m^2^ in ARIC to 34.5 kg/m^2^ in CFS). Half of AF participants had T2D due to the T2D case-control study design of AADM, which was also reflected in the mean BMI being higher than expected for the general population of the countries sampled. The T2D prevalence in the AA cohorts ranged from 11.2% (HUFS) to 28.3% (CFS). Mean circulating levels of GLP-1, PAI-1, visfatin, and IL-1RA were higher in AF than in AA. For leptin, GIP, resistin, insulin, and ghrelin, mean circulating levels were higher in AA compared with AF. Mean circulating levels were similar between AF and AA for adipsin, IL-6, IL-10, and glucagon.

**Table 1:** Characteristics of the study population, by cohort

	Discovery cohort	Replication cohorts				
	AADM (***n*** = 4432)	HUFS (***n*** = 1958)	ARIC (***n*** = 3029)	CFS (***n*** = 304)	JHS (***n*** = 1281)	MESA (***n*** = 1418)
**Covariates**						
Age (years)	51.5 (51.1–51.9)	39.4 (38.6–40.1)	53.3 (53.1–52.6)	45.1 (43.3–46.9)	49.4 (48.7–50.1)	62.1 (61.5–62.6)
Sex (% female)	59.4 (58.0–60.8)	61.2 (59.1–63.4)	62.6 (60.9–64.3)	58.6 (52.9–64.0)	60.5 (57.8–63.1)	53.5 (50.9–56.0)
BMI (kg/m^2^)	26.5 (26.4–26.7)	30.0 (29.6–30.3)	29.7 (29.4–29.9)	34.5 (33.5–35.5)	32.3 (31.9–32.7)	30.2 (29.9–30.5)
T2D (%)^a^	49.9 (48.4–51.4)	11.2 (9.9–12.7)	21.7 (20.3–23.2)	28.3 (23.5–33.6)	12.8 (11.1–14.7)	17.8 (15.9–19.9)
**Cytokines**						
Adipsin (ng/ml)	1205.5 (898.5–2095.5)	1153.4 (920.8–1380.9)	--	--	--	--
Leptin (ng/ml)	3.7 (1.0–10.2)	14.1 (3.9–40.0)	--	--	--	--
GIP (pg/ml)	207.0 (134.9–336.9)	480.8 (346.7–694.3)	--	--	--	--
GLP-1(pg/ml)	224.1 (176.4–289.7)	153.6 (124.8–182.3)	--	--	--	--
PAI-1(ng/ml)	32.0 (22.6–47.4)	*NA*	14.0 (5.6–24.2)	29.1 (13.1–55.9)	--	16.0 (8.0–33.0)
Resistin (ng/ml)	4.8 (3.2–7.5)	5.7 (4.0–9.1)	--	--	--	--
Visfatin (ng/ml)	2.3 (1.5–3.9)	1.33 (1.06–1.62)	--	--	--	--
Il-1RA (pg/ml)	320.7 (237.7–463.4)	270.8 (152.6–427.1)	--	--	--	--
IL-6 (pg/ml)	1.06 (0.72–1.70)	1.23 (0.75–2.1)	--	2.35 (1.4–3.9)	--	1.36 (0.89–2.14)
IL-10 (pg/ml)	9.6 (7.7–12.0)	9.8 (7.8–12.0)	--	--	--	--
**Hormones**						
Insulin (uU/ml)^b^	5.7 (3.1–10.1)	7.9 (4.6–13.4)	11.4 (7.2–17.6)	--	14.0 (10.0–21.0)	8.4 (5.9–12.3)
Glucagon (pg/ml)	280.5 (176.2–449.2)	286.8 (216.6–363.9)	--	--	--	--
Ghrelin (pg/ml)	311.0 (160.1–685.8)	944.7 (754.2–1280.2)	--	--	--	--

Continuous variables are in means and corresponding (95% confidence intervals) for normally distributed variables. Categorical variables are in percentages with corresponding (95% confidence intervals). Non-normally distributed variables are expressed in medians and (25th–75th percentile)

^a^The high T2D prevalence in AADM is due to the T2D case-control study design

^b^T2D cases were excluded for all insulin analyses

*T2D* type 2 diabetes; --, not available

### Heritability of the obesity and diabetes-related cytokines and hormones in African ancestry individuals

We calculated SNP-heritability for each of the adipocytokines and hormones in AF. Heritability was highest for glucagon (70.6%) and lowest for PAI-1 (13.3%) (Table 2). No reliable heritability estimates could be derived for IL-1RA, IL-6, and IL-10 due to the relatively low sample size for these interleukins.

**Table 2:** Heritability estimates with corresponding 95% confidence intervals for the 13 obesity- and diabetes-related cytokines and hormones in continental Africans with comparison of heritability estimates in other populations

	Continental Africans				Other populations				
Trait	Heritability %	SE	95% CI	***n***	Population	Heritability %	Method	***n***	Ref
Adipsin	62.8	11.5	40.3–85.3	2761					
Leptin	32.9	12.5	8.4–57.4	2808	Hispanic	25	Variance component analyses	1030	[56]
GIP	47.6	11.9	24.3–70.9	2810	Swedish	15	GCTA	3344	[24]
GLP-1	21.9	12.2	− 2.0–45.8	2834	Swedish	0	GCTA	3344	[24]
PAI-1	13.3	11.9	− 10.0–36.6	2847	British	20	Variance component analyses	537	[57]
Resistin	24.8	11.0	3.2–46.4	1814	Italian	68	Variance component analyses	264	[58]
					European American	35	Quantitative trait simulation	2531	[59]
Visfatin	53.6	12.2	29.7–77.5	2803					
Il-1RA^a^	--	--	--	1179					
IL-6^a^	--	--	--	1125					
IL-10^a^	--	--	--	881					
Insulin^b^	46.7	11.4	24.4–69.0	2963					
					Swedish	10.6	GCTA	3344	[24]
					Hispanics	19	Variance components decomposition	1030	[56]
Glucagon	70.6	11.7	47.7–93.5	2796	Swedish	20	GCTA	3344	[24]
Ghrelin	58.6	11.1	36.8–80.4	2830	Hispanics	61	Variance components decomposition	1030	[56]

^a^No reliable estimate could be derived due to the low sample size for these interleukins

^b^T2D cases were excluded for all Insulin analyses

*SE* standard error; *CI* confidence interval; *n*, sample size; --, not available; *GCTA* Genome-Wide Complex Trait Analysis; *Ref* reference

The SNP heritability estimates for the adipocytokines and hormones in AF differ for several traits from what has been reported among other populations (Table 2). Heritability in AF was higher for GIP, GLP-1, insulin, and glucagon compared with other populations [24, 56]. For resistin, heritability was lower in AF compared with other populations [58, 59]. Ghrelin, PAI-1, and leptin heritability were comparable with estimates from other populations [56, 57]. No previously reported heritability estimates for adipsin and visfatin were found.

### Genetic loci associated with obesity and diabetes-related cytokines and hormones

**Fig. 1:**
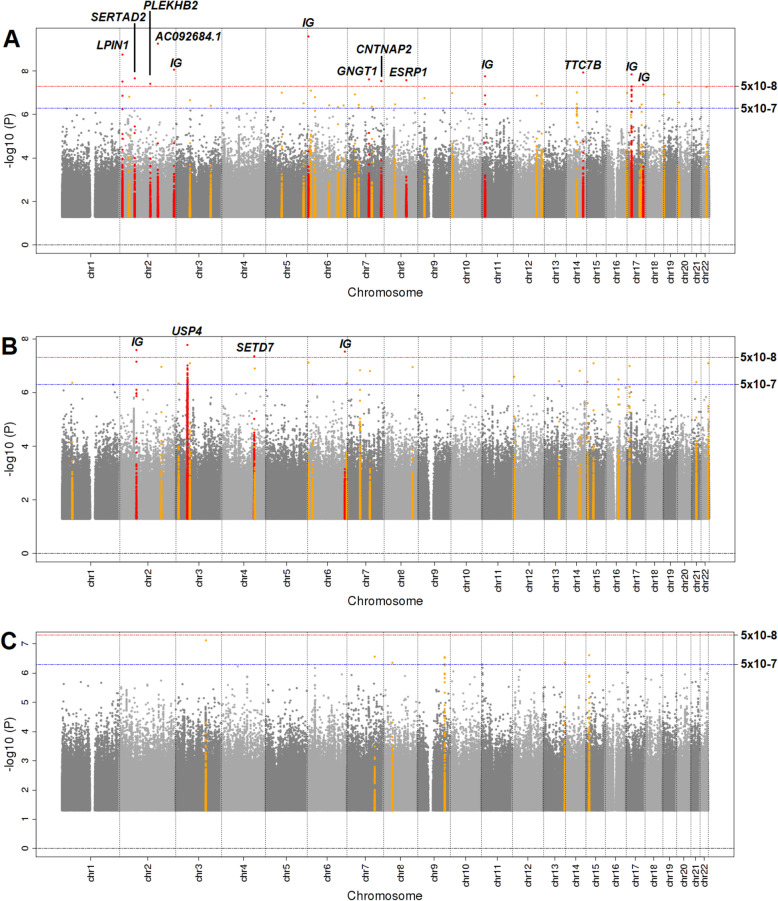
Manhattan plots for genome-wide associations with adipsin (**A**), ghrelin (**B**), and visfatin (**C**) in continental Africans. Loci in red reached genome-wide significance at *P* value <5 × 10^−8^, loci in orange are considered suggestive at *P* value < 5 × 10^−7^. IG, intergenic

We performed the first GWAS for 13 adipocytokines and hormones in African-ancestry populations. An overview of the number of loci discovered per stratum and per trait, as well as the number of replicated loci, can be found in Additional File 1: Table S2. Figure 1 shows the Manhattan plots for adipsin (Fig. 1A), ghrelin (Fig. 1B), and visfatin (Fig. 1C), for which this was the first GWAS in any population. We detected 39 loci across all traits that passed the genome-wide significance threshold of *P* value < 5 × 10^−8^ and four loci that passed a more stringent *P* value threshold adjusted for the number of traits and the stratification (Table 3). Regression analyses of genome-wide significant variants with a low MAF (< 5%) were checked for high leverage points using Cook’s distance, but removal of the few outliers identified did not alter the results (Additional File 1: Table S3). Regional plots created using *LocusZoom* [60] for all 39 loci can be found in Additional File 2: Fig S2. For IL-10 and visfatin, no variants reached the genome-wide significance threshold of *P* value < 5 × 10^−8^. Three of the 39 detected loci were known: a locus in an intron of the *LEP* gene for leptin [26], a locus in an intron of *TRIM56* for PAI-1 [61, 62], and a locus in the promoter of *RETN* for resistin [63–65] (Table 4). Replication in AA could not be attempted for 10 of the 36 novel loci because the variant had been filtered out in the AA dataset either due to low MAF (< 0.01) or during quality control. We replicated one locus in AA for adipsin: locus *AC092684.1* (best represented by variant rs201751833) on chromosome 2, replicated in AA at a *P* value of 0.002 (Table 4 and Additional File 2: Fig S2). This locus did not pass a more stringent threshold adjusted for the number of replication loci evaluated (*P* value < 0.0017). Across adipocytokines and hormones, associations were detected with 14 African-specific variants, i.e., variants that are observed in African populations but are not observed in populations without African ancestry in the Allele Frequency Aggregator (ALFA) database (Table 3). In meta-analysis of discovery and replication cohorts combined, we detected 16 loci across 10 of the 13 traits that passed the threshold for genome-wide significance and six that passed the more stringent threshold (Additional File 1: Table S4, Additional File 2: Fig S3). Six of these 16 genome-wide significant loci were also detected in the discovery analysis at a *P* value of < 5 × 10^−8^.

**Table 3:** Variants at genome-wide significance of *P* value < 5 × 10^−8^ per adipocytokine/hormone for the base model

SNP	Chr	Position GRCh37	Alleles ref/tested	NS	MAF	AFR/EAS/EUR/AMR	INFO	beta	SE	***P*** value	Functional class: gene
**Adipsin**											
rs199890456	6	2914945	C/T	3402	0.2173	0.00/0.00/0.00/0.00	0.61	− 0.230	0.036	**2.59E**−**10**	Intergenic
rs201751833	2	165079139	T/G	3713	0.2579	0.35/0.09/0.15/0.67	0.80	− 0.191	0.031	**5.51E**−**10**	Intron:*AC092684.1*
rs1469952	2	11917519	G/A	3713	0.2983	0.50/0.00/0.56/0.00	0.99	− 0.159	0.026	1.76E−09	Utr5:*LPIN1*
rs115100304	2	234777140	C/G	3713	0.0265	0.01/0.00/0.00/0.00	0.98	− 0.438	0.076	8.76E−09	Intergenic
rs34061423	14	91095586	C/CT,CAT	3713	0.2480	NA	0.92	− 0.172	0.030	1.17E−08	Insertion:*TTC7B*
rs183172404	17	20247428	G/A	3713	0.0392	0.05/0.00/0.00/0.00	0.91	0.371	0.065	1.44E−08	Intergenic
rs539334014	11	13192725	G/A,T	3713	0.0234	0.00/0.00/0.01/0.00	0.60	− 0.549	0.097	1.76E−08	Intergenic
rs79750258	2	64909796	T/G	3713	0.1776	0.05/0.12/0.42/0.11	0.61	− 0.210	0.037	2.19E−08	Intron:*SERTAD2*
rs111651263	7	93239919	T/C	3713	0.0560	0.04/0.00/0.00/0.00	0.99	− 0.310	0.055	2.44E−08	Intron:*GNGT1*
rs79024755	8	95715336	G/T	3713	0.1746	0.25/0.00/0.01/0.00	0.68	− 0.195	0.035	2.64E−08	Intron:*ESRP1*
rs113529034	7	146458922	T/C	3713	0.2346	NA	0.83	− 0.179	0.032	2.90E−08	Intron:*CNTNAP2*
rs180913374	2	132099516	C/T	3713	0.0116	0.00/0.00/0.01/0.00	0.60	0.734	0.133	3.87E−08	Intron:*PLEKHB2*
rs145662005	17	70334651	G/C	3713	0.0122	0.00/0.00/0.00/0.00	0.67	0.698	0.127	4.20E−08	Intergenic
**Leptin**											
rs28954105	7	127891616	G/T	3745	0.1119	0.08/0.00/0.00/0.00	0.99	− 0.219	0.038	6.77E−09	Intron:*LEP*
rs113453972	14	64123318	G/T	3745	0.0538	0.05/0.00/0.00/0.00	0.99	− 0.298	0.053	1.88E−08	Intergenic
rs61258383	1	88355364	A/G	3745	0.0531	0.29/0.13/0.31/0.25	0.95	0.300	0.054	3.55E−08	Intergenic
rs9894577	17	43223292	G/A	3745	0.3203	0.31/0.23/0.32/0.34	0.99	− 0.140	0.026	4.41E−08	Intergenic
**GIP**											
rs12028926	1	8818438	C/G	3751	0.0183	0.08/1.00/0.27/0.00	0.77	− 0.559	0.094	3.00E−09	Intron:*RERE*
rs62266118	3	115124821	A/G	3751	0.0211	0.06/0.22/0.21/0.00	0.75	− 0.500	0.085	5.12E−09	Intergenic
rs17437121	1	23640663	A/G	3751	0.0219	0.03/0.00/0.15/0.10	0.81	0.499	0.090	3.46E−08	Intron:*HNRNPR*
rs17335662	11	11062119	C/T	3751	0.0162	0.06/0.00/0.22/0.00	0.93	− 0.506	0.092	3.86E−08	Intergenic
rs182578321	9	85708499	C/T	3751	0.0120	0.01/0.00/0.00/0.00	0.91	0.611	0.111	4.06E−08	Intergenic
rs988623129	18	44187314	ATACATATATA CATATATG/A	3751	0.0229	NA	0.88	0.460	0.084	4.56E−08	Deletion:*LOXHD1*
**GLP-1**											
rs1355371392	2	135283403	C/A	1918	0.0507	0.00/0.00/0.00/0.00	0.92	− 0.476	0.080	2.47E−09	Intron:*TMEM163*
rs73669122	9	129313248	C/T	3783	0.1365	0.04/0.00/0.00/0.00	0.93	0.202	0.036	1.41E−08	Intergenic
rs1450571579	1	93402186	C/A	3783	0.0515	NA	0.61	− 0.382	0.068	2.15E−08	Intron:*FAM69A*
rs1445210817	14	64071897	T/C	3783	0.0155	0.00/0.00/0.00/0.00	0.48	− 0.704	0.128	3.75E−08	Intron:*WDR89*
rs372701742	8	25463403	T/TA	3783	0.0250	NA	0.61	0.540	0.098	4.23E−08	Intergenic
**PAI-1**											
rs113421429	7	100729247	C/T	3807	0.0978	0.18/0.00/0.00/0.00	1.00	− 0.270	0.041	**5.86E**−**11**	Intron:*TRIM56*
rs61654751	19	3436093	TG/T	3807	0.1056	0.13/0.00/0.00/0.00	0.95	− 0.238	0.041	5.58E−09	Deletion:*NFIC*
rs2496687	6	81340947	C/G	3807	0.7965	0.76/1.00/0.91/0.00	0.99	− 0.169	0.030	2.40E−08	Intergenic
**IL1-RA**											
rs202017265	18	13515503	A/T	1339	0.1543	0.21/0.27/0.29/0.17	0.71	− 0.347	0.063	3.91E−08	Exon:*RP11-53B2.3*
**IL-6**											
rs72911283	18	8243353	T/G	1263	0.0213	0.01/0.00/0.07/0.08	0.93	− 0.793	0.143	3.24E−08	Intron:*PTPRM*
**Glucagon**											
rs10809430	9	11345807	C/G	3730	0.0138	0.00/0.00/0.00/0.00	0.92	0.590	0.106	2.91E−08	Intergenic
**Ghrelin**											
3:49318960	3	49318960	C/T	3777	0.0128	NA	0.84	0.616	0.109	1.67E−08	Intron:*USP4*
rs150921599	2	72385239	C/CA	3777	0.1089	0.04/0.00/0.00/0.00	0.98	0.208	0.037	2.60E−08	Intergenic
rs183921098	6	159981730	G/A	3777	0.0101	0.01/0.00/0.00/0.00	0.88	0.663	0.119	2.97E−08	Intergenic
rs372331930	4	140418751	G/GT	3777	0.0343	0.06/0.01/0.00/0.02	0.89	0.365	0.067	4.42E−08	Insertion:*SETD7*
**Resistin**											
rs3219175	19	7733855	G/A	3754	0.0760	0.14/0.17/0.01/0.01	0.84	0.777	0.034	**5.01E**−**111**	Promotor:*RETN*

Reported allele frequencies are from the Allele Frequency Aggregator (ALFA) (version 20200227123210) and from the 1000 Genomes Project (phase3 release V3+) when ALFA frequencies were not available

*P* values of loci passing the more stringent significance threshold of 1.32 × 10^−9^ have been indicated in bold

*SNP* single nucleotide polymorphism; *Chr* chromosome; *NS* number of samples; *MAF* minor allele frequency; *AFR* African; *EAS* East Asian; *EUR* European; *AMR* American; *INFO* imputation info score; *SE* standard error for the beta; *NA* data not available

**Table 4:** Replication in African Americans and GWAS catalog of genome-wide significant loci per adipocytokine/hormone for the base model

				Replication in AA						Replication in GWAS catalog
SNP	Chr	Position GRCh37	Alleles ref/tested	NS	MAF	***P*** value	beta	SE	Heterogeneity ***P*** value	PMID: population(s)
**Adipsin**										
rs199890456	6	2914945	C/T	--	--	--	--	--	--	*(novel)*
rs201751833	2	165079139	T/G	1846	0.4782	**0.002**	− 0.113	0.037	--	*(novel)*
rs1469952	2	11917519	G/A	1846	0.4951	0.866	0.006	0.035	--	*(novel)*
rs115100304	2	234777140	C/G	1846	0.0411	0.942	− 0.006	0.089	--	*(novel)*
rs34061423	14	91095586	C/CT,CAT	1846	0.3599	0.508	− 0.024	0.037	--	*(novel)*
rs183172404	17	20247428	G/A	1846	0.0389	0.554	− 0.057	0.097	--	*(novel)*
rs539334014	11	13192725	G/A,T	--	--	--	--	--	--	*(novel)*
rs79750258	2	64909796	T/G	--	--	--	--	--	--	*(novel)*
rs111651263	7	93239919	T/C	1846	0.0842	0.766	0.021	0.071	--	*(novel)*
rs79024755	8	95715336	G/T	1846	0.2848	0.747	− 0.016	0.048	--	*(novel)*
rs113529034	7	146458922	T/C	1846	0.0245	0.169	− 0.211	0.153	--	*(novel)*
rs180913374	2	132099516	C/T	--	--	--	--	--	--	*(novel)*
rs145662005	17	70334651	G/C	--	--	--	--	--	--	*(novel)*
**Leptin**										
rs28954105	7	127891616	G/T	1869	0.0822	**0.001**	− 0.214	0.063	--	26833098: European ancestry
rs113453972	14	64123318	G/T	1869	0.0398	0.476	− 0.064	0.090	--	*(novel)*
rs61258383	1	88355364	A/G	1869	0.0469	0.864	− 0.014	0.082	--	*(novel)*
rs9894577	17	43223292	G/A	1869	0.2878	0.897	− 0.005	0.039	--	*(novel)*
**GIP**										
rs12028926	1	8818438	C/G	1859	0.0523	0.797	− 0.021	0.081	--	*(novel)*
rs62266118	3	115124821	A/G	1859	0.0376	0.599	0.052	0.099	--	*(novel)*
rs17437121	1	23640663	A/G	1859	0.0360	0.798	0.026	0.103	--	*(novel)*
rs17335662	11	11062119	C/T	1859	0.0495	0.567	0.048	0.085	--	*(novel)*
rs182578321	9	85708499	C/T	--	--	--	--	--	--	*(novel)*
rs988623129	18	44187314	ATACATATATA CATATATG/A	1859	0.0114	0.578	- 0.101	0.182	--	*(novel)*
**GLP-1**										
rs1355371392	2	135283403	C/A	--	--	--	--	--	--	*(novel)*
rs73669122	9	129313248	C/T	1798	0.1029	0.348	− 0.058	0.062	--	*(novel)*
rs1450571579	1	93402186	C/A	1798	0.0426	0.259	0.129	0.114	--	*(novel)*
rs1445210817	14	64071897	T/C	--	--	--	--	--	--	*(novel)*
rs372701742	8	25463403	T/TA	1798	0.0270	0.682	−0.062	0.152	--	*(novel)*
**PAI-1**										
rs113421429	7	100729247	C/T	594	0.1641	0.103	0.151	0.093	0.869	24578379 & 22990020: European ancestry
rs61654751	19	3436093	TG/T	594	0.1299	0.185	0.179	0.135	0.310	*(novel)*
rs2496687	6	81340947	C/G	594	0.7799	0.122	0.109	0.071	0.488	*(novel)*
**IL1-RA**										
rs202017265	18	13515503	A/T	533	0.3761	0.308	− 0.066	0.064	--	*(novel)*
**IL-6**										
rs72911283	18	8243353	T/G	2235	0.0208	0.094	0.112	0.401	0.166	*(novel)*
**Glucagon**										
rs10809430	9	11345807	C/G	1865	0.0458	0.661	− 0.036	0.082	--	*(novel)*
**Ghrelin**										
3:49318960	3	49318960	C/T	--	--	--	--	--	--	*(novel)*
rs150921599	2	72385239	C/CA	1858	0.0897	0.354	− 0.058	0.062	--	*(novel)*
rs183921098	6	159981730	G/A	--	--	--	--	--	--	*(novel)*
rs372331930	4	140418751	G/GT	1858	0.0340	0.809	− 0.025	0.104	--	*(novel)*
**Resistin**										
rs3219175	19	7733855	G/A	1867	0.1157	**9.52E−38**	0.804	0.061	--	24123702, 22843503 & 27664181: Han Chinese, European ancestry, Japanese

-- could not be replicated in AA because the variant had been filtered out in the AA dataset either due to low MAF (< 0.01) or during the quality control

*AA* African American; *SNP* single nucleotide polymorphism; *Chr* chromosome; *NS* number of samples; *MAF* minor allele frequency; *SE* standard error for the beta

### Evidence for colocalization of GWAS findings and eQTL data

We performed Bayesian colocalization analyses on our association summary statistics and eQTL data on 49 tissues from the GTEx portal to quantify the probability that eQTL signals at genome-wide loci share a single causal variant. Across traits, a total of 18 genome-wide loci showed strong evidence for colocalization in one or more tissues and one locus showed moderate evidence (Additional File 1: Table S5). Subcutaneous and visceral omentum adipose tissues were the predominant tissue types showing convincing evidence for colocalization. No colocalizing genes were identified for the novel replicated locus (rs201751833) nor for the four loci meeting the more stringent GWAS *P* value threshold.

### The effect of sex, BMI status, and T2D status on GWAS findings for the obesity-related cytokines and hormones

We found associations that differed markedly by sex, BMI status and T2D status. All detected loci differed between strata, except for the known PAI-1 and resistin loci (annotated to genes *TRIM56* and *RETN* respectively) and the leptin loci for the lean and overweight models (Additional File 2: Fig S4-S6). Most loci differing between strata (90%) showed significant heterogeneity (*P* values < 0.05) when meta-analyzed together (Additional File 1: Tables S6-S11). We detected 49 loci associated with one or more of the 13 obesity and diabetes-related cytokines and hormones among men (Additional File 1: Table S6), 40 among women (Additional File 1: Table S7), 42 among lean individuals (Additional File 1: Table S8), 50 loci among overweight individuals (Additional File 1: Table S9), 53 among T2D controls (Additional File 1: Table S10), and 34 among T2D cases (Additional File 1: Table S11). Using a more stringent *P* value threshold adjusting for the number of traits and the stratification, we detected five loci among men, seven among women, 10 among lean individuals, seven among overweight individuals, six among T2D controls, and five among T2D cases (Additional File 1: Tables S6-S11).

#### The effect of sex

**Fig. 2:**
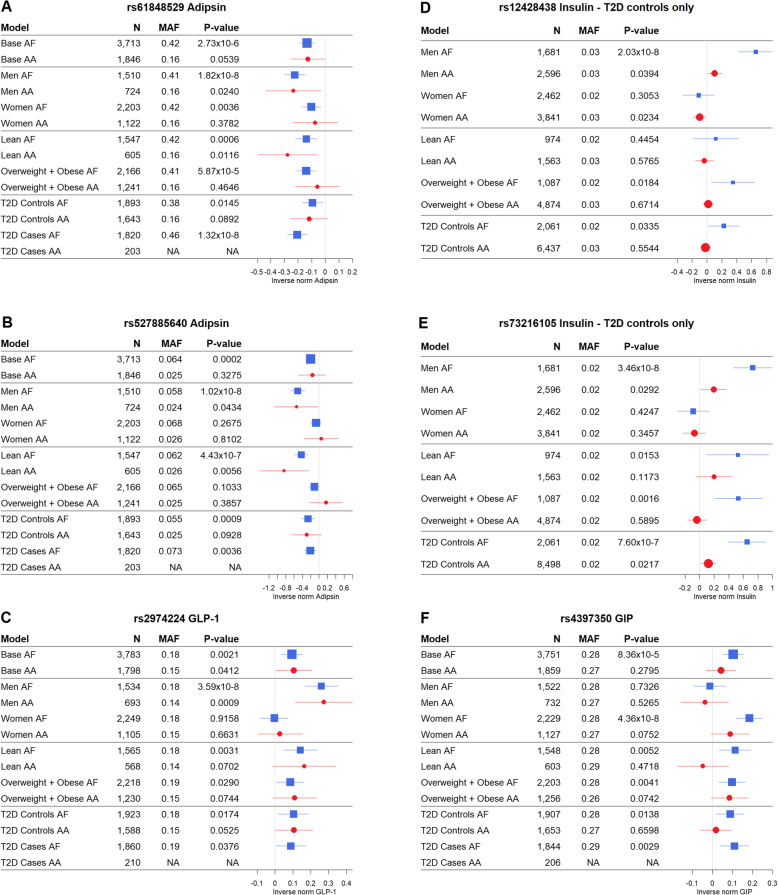
Forest plots illustrating for each model the beta and 95% confidence interval of replicated novel variants that showed sex-specific effects. Where multiple variants were statistically significant in one locus, the replicated variant with the lowest *P* value has been displayed. T2D controls only = insulin analyses were not undertaken in T2D cases because of the potential of fasting insulin levels to be altered as part of the T2D pathology

Sex was most relevant for GIP and PAI-1, for which we respectively detected nine and five genome-wide significant loci in women, but none among men (Additional File 2: Fig S4). Among men, we detected a known locus (*CHL*) for insulin, which has been reported previously in European-ancestry populations [23]. Five of the 47 novel loci detected in men replicated in AA. The associations for each of the five replicated loci per model are displayed in Fig. 2A-2E. Two of the five replicated loci were adipsin loci that replicated in AA: an intergenic locus on chromosome 10 (rs61848529) (*P* value of 0.024) and *KIR3DL1* on chromosome 19 (*P* value 0.044) (Fig. 2A and B, Additional File 1: Table S6, Additional File 2: Fig S2). Interaction tests revealed that the larger effect size of rs61848529 for adipsin in men compared to women was not significant for both the GG genotype (*P* value = 0.054) and the AG genotype (*P* value = 0.745). The variant annotated to *KIR3DL1* did show a significant differential effect on adipsin in men compared with women for both the AA genotype (*P* value of interaction = 0.023) and for the AG genotype (*P* value of interaction = 0.036) compared with the GG genotype. A locus associated with GLP-1 in men (*GLTSCR1*) was replicated in AA (*P* value 0.0009) (Fig. 2C, Additional File 1: Table S6, Additional File 2: Fig S2). This replicated locus additionally passed a more stringent threshold adjusted for number or independent loci attempted for replication (*P* value < 0.0019). The association of this locus with GLP-1 also had a larger effect size in men than in women for both the CC genotype (*P* value of interaction = 0.003) and the AG genotype (*P* value of interaction = 0.001) compared with the AA genotype. For insulin, two men-specific novel loci were replicated in AA: locus *NALCN-AS1* on chromosome 19 (*P* value AA = 0.039) (Fig. 2D, Additional File 1: Table S6, Additional File 2: Fig S2) and an intergenic locus (rs73216105) on chromosome 12 (*P* value AA = 0.029) (Fig. 2E, Additional File 1: Table S6, Additional File 2: Fig S2). The larger effect sizes in men compared with women for both loci were confirmed to be statistically significant in interaction tests (all interaction *P* values < 0.025). One women-specific intergenic locus replicated in AA (Fig. 2F, Additional File 2: Fig S2). In exact replication, the variant best representing this locus for GIP did not reach statistical significance (rs4397350, *P* value 0.075), but in LD-based local replication the variant rs62460948 was replicated at *P* value 0.021 (LD-corrected *P* value threshold = 0.037). This locus showed a larger effect size in women for both the CC genotype (*P* value of interaction = 0.000) and the CT genotype (*P* value of interaction = 0.030) compared with the TT genotype.

#### The effect of BMI status

**Fig. 3:**
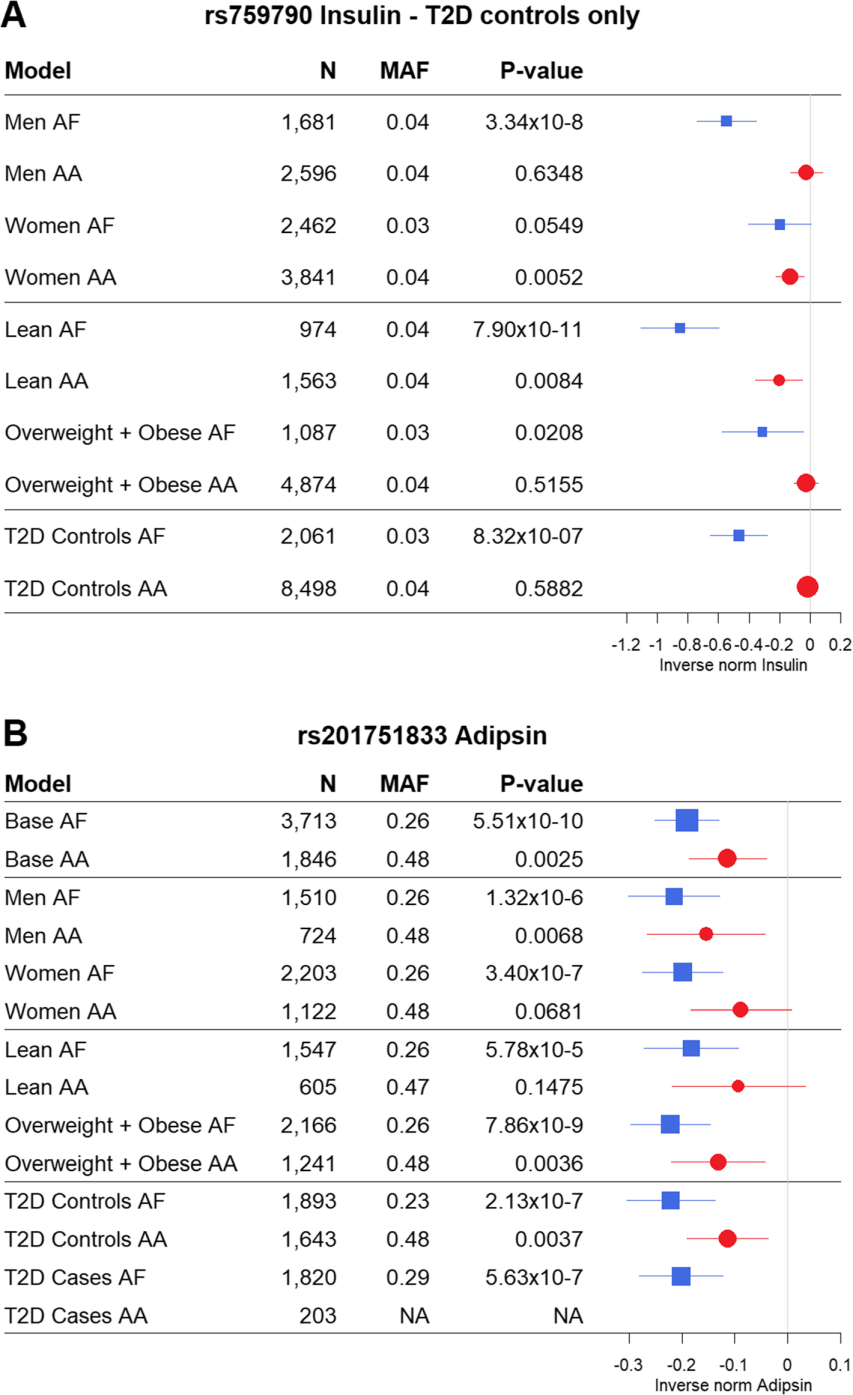
Forest plots illustrating for each model the beta and 95% confidence interval of replicated novel variants that showed BMI specific effects. Where multiple variants were statistically significant in one locus, the replicated variant with the lowest *P* value has been displayed. T2D controls only = insulin analyses were not undertaken in T2D cases because of the potential of fasting insulin levels to be altered as part of the T2D pathology

BMI status had a strong effect on all included adipocytokines and hormones, except leptin (Additional File 1: Tables S8 and S9, Additional File 2: Fig S5). Three loci were found strongly associated with leptin in both lean (BMI < 25 kg/m^2^) and overweight (BMI ≥ 25 kg/m^2^) individuals. We replicated one novel lean-specific locus associated with insulin (rs759790, AA *P* value 0.0084) (Fig. 3A, Additional File 1: Table S8, Additional File 2: Fig S2) and one novel overweight-specific locus. *AC092684.1*, associated with adipsin, replicated in AA at a *P* value of 0.0036 (Fig. 3B, Additional File 1: Table S9, Additional File 2: Fig S2). This is the same variant (rs201751833) as was replicated for adipsin in the base (non-stratified) model. The replicated lean-specific locus for insulin (rs759790) showed a differential association between lean and overweight individuals (Fig. 3A), which was confirmed in statistical interaction tests (*P* value = 0.001) that showed that the AC genotype compared with the CC genotype had a significantly larger effect size for the association with insulin in lean compared with overweight individuals. The effect of the AA genotype compared with CC did not significantly differ between lean and overweight individuals (*P* value 0.907). The replicated overweight-specific locus for adipsin did not show differential associations between lean and overweight individuals (*P* values for interaction > 0.1) due to reduced sample size in the lean stratum (*n* = 1547) compared with the overweight (*n* = 2166) (Fig. 3B).

#### The effect of T2D status

T2D status was particularly relevant for visfatin. While no genome-wide significant loci were detected for visfatin in the base model and only one locus in the T2D controls, seven loci were detected in T2D cases (Additional File 2: Fig S6). Notably, an African-specific locus (rs577401632) annotated to gene *ZRANB3* was detected for glucagon in T2D controls only. *ZRANB3* has recently been identified as an African-specific T2D locus associated with beta-cell mass and insulin response [31]. This variant could not be replicated in AA as the variant had been filtered out in AA because of low imputation quality (INFO = 0.29). None of the other 33 novel loci in T2D controls that could be replicated were replicated in AA (Additional File 1: Table S2 and Table S10). As T2D cases were excluded from all insulin analyses, T2D case specific analyses were not performed for insulin (Additional File 1: Table S11). Replication in AA was not performed for T2D cases as the number of T2D cases in the AA cohorts was insufficient.

### Phenotypic and genetic correlation among obesity and diabetes-related cytokines and hormones

**Fig. 4:**
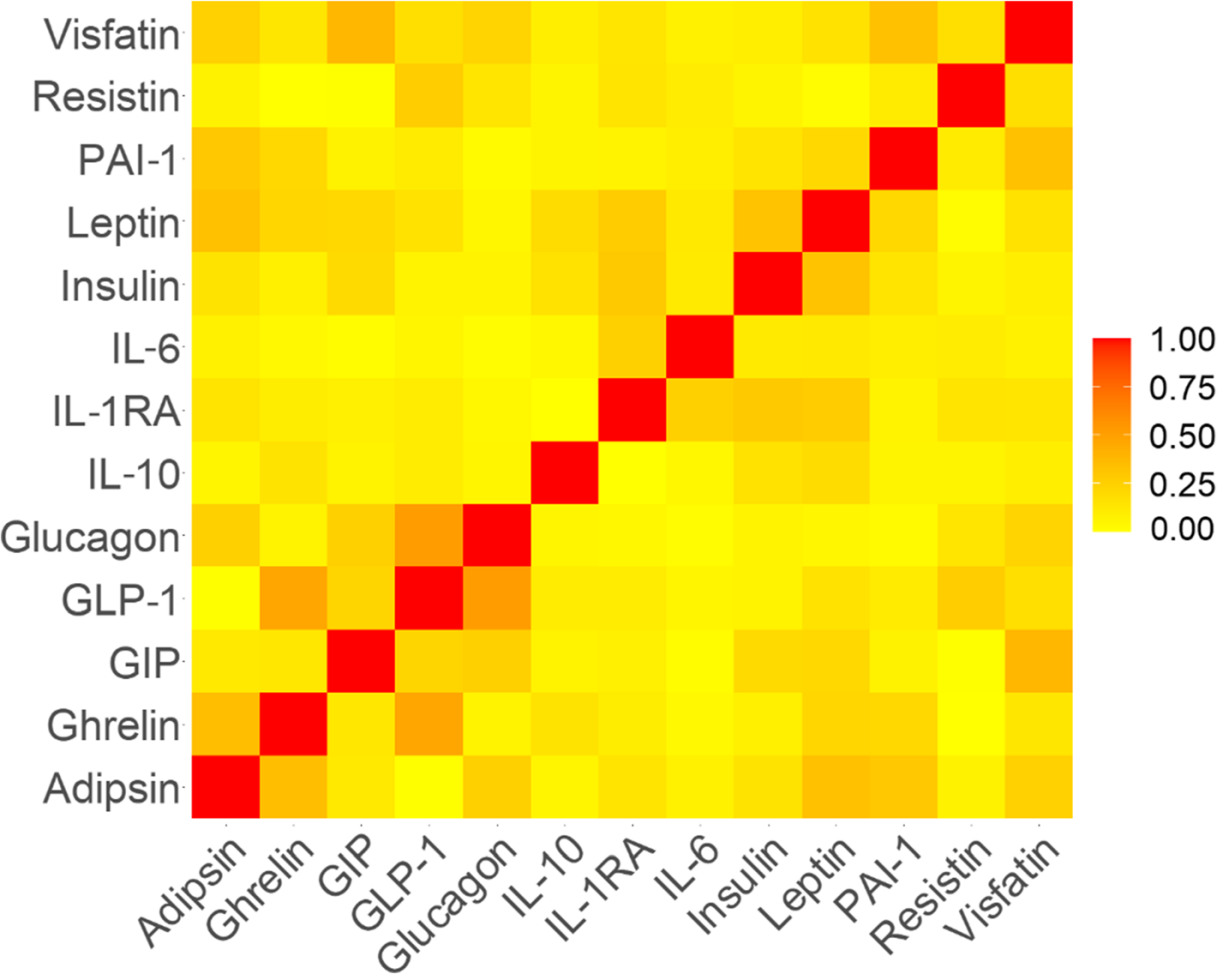
Correlation heatmap of the 13 obesity- and diabetes-related cytokines and hormones studied in continental Africans

Both phenotypic and genetic correlation between the studied cytokines and hormones was low. Most adipocytokines and hormones were very weak (*r*^2^ < 0.20) or weakly (*r*^2^ = 0.20–0.39) correlated with each other (Fig. 4). Adipsin and ghrelin, ghrelin and GLP-1, as well as GLP-1 and glucagon showed moderate correlation (*r*^2^ = 0.40–0.59), whereas none of the adipocytokines and hormones showed high correlation (*r*^2^ ≥ 0.60).

**Fig. 5:**
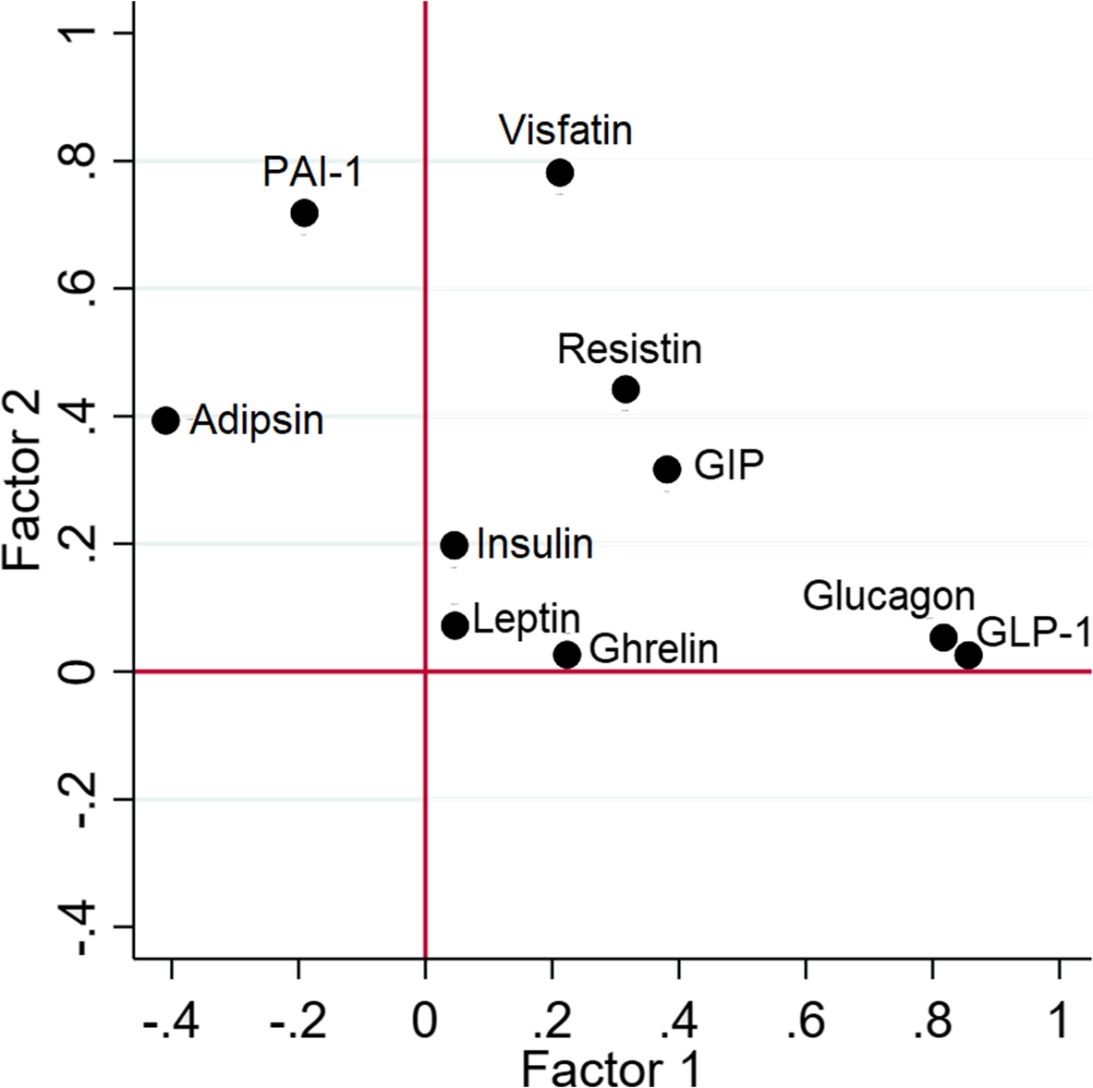
Varimax rotated factor loadings for 10 obesity- and diabetes-related cytokines and hormones in continental Africans

The Bartlett test of sphericity indicated that sufficient correlation existed to perform factor analysis (*P* value < 0.001). The interleukins (IL-1RA, IL-6, and IL-10) were excluded from factor analyses because of low sample size. Factor analysis was performed on the 1538 samples that had complete data on all 10 remaining adipocytokines and hormones and revealed four factors with an eigenvalue of < 1 that combined explained 65% of variance. Traits GLP-1, glucagon, and GIP were related to the first factor, PAI-1 and visfatin to the second factor, ghrelin and adipsin to the third factor, and insulin and leptin to the fourth factor. Resistin did not relate well to any of the factors. Figure 5 shows the rotated factor loadings for each of the traits. Consistent with our expectations that several of these traits are biologically independent, the internal consistency of the 10 cytokines and hormones included in the factor analysis was low with a Cronbach’s alpha of 0.67.

In addition, there was little evidence of pleiotropic loci influencing multiple adipocytokines and hormones. In the base model, only one locus overlapped (within 500 kb region) in the results across traits. GLP-1 variant rs1445210817 is 51.4 kb away from leptin variant rs113453972. These variants were not in LD (*R*^2^ = 0.0004). The GLP-1 variant is annotated as in an intron of *WDR89* and the leptin variant is intergenic. In the stratified models, we found three additional loci that were physically close of which one had an *R*^2^ > 0.3. Variant rs75275574, associated with visfatin in T2D cases, was 12 kb upstream of rs552964654, associated with IL-1RA in T2D controls (*R*^2^ = 0.34).

To further assess the genetic correlation of the traits, we used *Ingenuity Pathway Analysis* (IPA) to perform pathway analysis of genome-wide significant loci in the base model. We found three significant enriched canonical pathways when we evaluated genes annotated to genome-wide significant loci (*P* value < 5 × 10^−8^) (Additional File 1: Table S12). Only the most significant pathway (*P* value 0.007) included more than one gene; the *Adipogenesis Pathway* included the *LEP* gene from the leptin analysis and the *LPIN1* gene from the Adipsin analysis (Additional File 1: Table S12). When performing pathway analyses on all genes from the base model annotated to loci with a *P* value < 5 × 10^−7^, 15 significant pathways were identified. However, none of these pathways included more than three associated genes (Additional File 1: Table S12).

## Discussion

In this work, we report the genetic architecture of thirteen obesity- and diabetes-related cytokines and hormones in continental Africans. The heritability of these adipocytokines and hormones ranged from 13.3% for PAI-1 to 70.6% for glucagon. We report the first GWAS for these adipocytokines and hormones in Africans and for adipsin, ghrelin, and visfatin this is the first GWAS for these three adipocytokines reported in any population. We found 39 associations across traits, of which 36 represent novel loci. For ten of these 36 novel loci, replication could not be attempted because of low MAF in AA and out of the remaining 26 one adipsin locus (*AC092684.1*) was replicated in AA. Fourteen of the total 39 identified loci were African-ancestry specific. In addition, we found that genetic loci associated with the studied adipocytokines and hormones vary greatly between strata of phenotypes: men, women, lean, overweight, T2D controls, and T2D cases. An additional 237 loci were discovered in stratified models and 8 loci from stratified analyses were replicated in AA. Lastly, we observed low phenotypic as well as low genetic correlation between the traits.

The differences in heritability estimates for several of the adipocytokines and hormones in AF compared with other populations were expected as heritability is inherently population specific. Higher heritability in AF may be partly attributed to population differences in BMI as well as other environmental factors. Our African-specific cytokine and hormone heritability estimates contribute to the slowly growing body of population-specific SNP heritability estimates.

Two novel loci in the base model passed the stringent threshold for genome-wide significance (rs199890456 and *AC092684.1*). Both loci were associated with adipsin. Variant rs199890456 is African-ancestry specific and evaluation of the regional plot for this intergenic locus (Additional File 2: Fig S2) showed lack of association in a genotyped variant in high LD (*r*^2^ = 0.93). We, therefore, urge caution in interpreting this finding. Locus *AC092684.1* is one of four replicated loci that have previously been reported in relation to cardiometabolic traits in other populations and their annotation suggests possible mechanisms by which these variants may influence cardiometabolic traits. This locus, which we not only replicated in the base model but also in the overweight stratum for adipsin, has previously been associated with blood pressure among those with European ancestry, Asian ancestry and AA [66–69] as well as with cardiovascular disease [70] and hypertension [71]. Potentially, a simultaneous effect of variants in *AC092684.1* on both adipsin levels and blood pressure could explain variation in adipsin levels in relation to cardiometabolic traits. Using *Haploreg* v4.1 [72], we found that rs201751833 substantially alters a regulatory motif for Mef2 [73]. Mef2, or Myocyte enhancer factor–2, is a core cardiac transcription factor that plays a prominent role in cardiovascular development [74]. This is an example of how identification of loci associated with adipocytokines and hormones can contribute to our understanding of cardiometabolic traits.

The intergenic locus represented by rs61848529, replicated for adipsin in men, has been associated with diastolic blood pressure in a European-ancestry sample [75]. This variant, with a MAF of 0.41 in our sample of AF and 0.15 in AA, is not reported in ALFA nor in the 1000 Genomes Project. Using LD data from the AADM study, we found that the extent of the associated region is limited to the rs61848529 variant (Additional File 2: Fig S2), suggesting that this signal may not be robust.

Five variants in LD with each other (*r*^2^ ≥ 0.4) within the *KIR3DL1* locus reached genome-wide significance in AF men for adipsin and one of these, rs527885640, replicated in AA. rs78852323 and rs527885640, annotated to an intron of *KIR3DL1*, are African-ancestry specific and the alternative alleles were associated with lower adipsin levels. rs527885640 has previously been associated in Finnish-ancestry individuals with IL-7, an interleukin that plays an important role in B and T cell development as part of the immune respons e[76]. Adipsin is secreted by adipocytes and is essential in the activation of the immune system’s alternative complement pathway [77]. rs527885640 alters regulatory motifs for *JDP2* and *NFE2L 1*[78]. Silencing of *NFE2L1* was found to disrupt glucose metabolism and impair insulin secretion [79]. More work is needed to understand the interplay between adipsin and the immune system in relation to obesity and abnormal glucose metabolism.

Intergenic insulin locus rs73216105, which was detected and replicated in men, has been reported in relation to peak insulin response and acute insulin response in Hispanic, Europeans, and Pima Indians, but it did not reach genome-wide significance in these analyses (*P* values of 1.07 × 10^−6^ for peak insulin response and 3.15 × 10^−6^ for acute insulin response, populations combined) [80]. Multiple other studies reported this locus to be associated with T2D in Asian- and European-ancestry individuals [70, 81–84].

Thirty-three novel loci across stratified models passed a more stringent *P* value threshold adjusted for the number of traits and the stratification. For 17 of these 33 loci, data were available through the GTEx Portal V8 (accessed on December 23, 2020) [48] of which six had significant eQTL reported. Locus *TBCD* (rs139302892), associated with GIP in women, is an eQTL for *TBCD* in adipose tissue, whole blood, and muscle tissue, and for *FN3KRP* in multiple tissues including colon, adipose, heart, whole blood, and pancreas. The *FN3KRP* gene has been tentatively associated with glucose metabolism and T2D [85]. Intergenic locus rs112549844, associated with insulin in women, is an eQTL for *FAHD2CP* in muscle and heart tissue, which is a pseudogene. The insulin locus *RP11-31F15.2* in lean individuals is an eQTL for *SLC16A1* in adipose tissue. Overexpression of *SLC16A1* has in mouse pancreatic β cells been found to induce hyperinsulinism during exercise [86]. Locus rs746586 associated with GIP in T2D controls is an eQTL for *SLC24A4* in skin. An intergenic locus for adipsin in T2D cases (rs2853934) has been reported as an eQTL for 15 genes in multiple tissues including 4 *HLA* genes (*HLA-B*, *HLA-C*, *HLA-S*, *HLA-E*).

An intergenic insulin locus (rs759790) that passed the stringent *P* value threshold and that was replicated in lean individuals in our analyses is an eQTL for 18 different genes across multiple tissues. Most notable is the eQTL *GPAT2* in muscle, heart, and adipose tissue. *GPAT2* codes for the enzyme glycerol-3-phosphate acyltransferase (GPAT), which a rate-limiting enzyme in glycerolipid biosynthesis. GPATs have been reported to play a critical role in the development of obesity and insulin resistance [87]. This same locus has previously been associated with eosinophil counts [70]. An increase in eosinophils has been shown to be associated with a decreased risk of elevated fasting insulin and T2D [88], suggesting potential co-regulation.

Differences in environmental and genetic background between AF and AA may have limited our replication efforts. The AF participants of our discovery cohort were recruited in Ghana, Nigeria, and Kenya, which greatly differ in physical environment and related health behaviors such as physical activity, dietary intake, and smoking from the AA participants in the replication cohorts who reside in the USA. These environmental and genetic background differences between AF and AA highlight the need for more studies in AF in order to be able to replicate findings within this population.

Detection of loci linking adipocytokines and hormones to cardiometabolic traits was increased in the sex-stratified and BMI-stratified models. In addition, replication ability was higher in the sex-stratified model (13.3% replicated) compared with the non-stratified base model (3.8%) and the BMI-stratified models (3.6%). By stratifying we reduced the variability in the environmental context, which may have limited replication in the non-stratified model. These strata also show statistically significant heterogeneity according to an *I*-square value. The higher number of detected loci in the stratified models may, therefore, be an indication of the importance of sex, BMI, and T2D on these phenotypes: ignoring these factors by considering them in unstratified models may hamper the ability to detect and replicate association signals. Furthermore, the striking differences in detected loci between sub-groups of phenotypes (men, women, lean, overweight, T2D controls, and T2D cases) suggest that genetic variants for obesity and diabetes-related cytokines and hormones are specific to sub-groups. Previous studies have reported sex-specific SNP-trait associations for coronary artery disease and Crohn’s disease [89], longevity [90], anthropometrics [91], and lipid traits [92]. It has been suggested that blood-based biomarkers are of particular interest for discovery of novel loci by sex-stratified analyses that have previously been undetected in sex-combined analyses [28]. In this work, we confirmed that circulating adipocytokines and hormones show heterogeneity of SNP effects between sexes. Previous GWAS that stratified on T2D status detected and replicated loci associated with estimated glomerular filtration rate (eGFR) in T2D controls only, but found similar effect sizes in T2D cases and controls [93, 94]. In contrast, meta-analysis of the T2D strata in the present analyses revealed significant heterogeneity between the loci detected for each stratum. To our knowledge, we are the first to report GWAS stratified by BMI status for any trait.

Despite efforts to increase diversity in genomics studies, African-ancestry populations are still underrepresented in GWAS [95, 96]. Our findings reveal that some genetic loci involved in the regulation of obesity- and diabetes-related cytokines and hormones seem to be population-specific. Fourteen out of the 39 loci detected in the base models for the 13 cytokines and hormones were best represented by variants that are African-specific. These loci may play a role in the previously reported ethnic differences in circulating levels of these adipocytokines and hormones [11, 12, 14, 15]. Furthermore, we observed a preponderance of low allele frequency variants among our findings. This preponderance has been reported previously in analyses of African ancestry individuals [97–99], highlighting the need for more studies in African-ancestry populations to be able to study the phenomenon. The underrepresentation of African individuals in databases such as GTEx [48] further impairs insight into the functional relevance of detected variants. Further studies that include multi-omics data are needed to unravel whether the detected loci are involved in the high burden of obesity and related cardiometabolic disorders in African-ancestry populations.

A recent study reporting a GWAS of a cytokines network found shared causal variants between cytokines [100]. In contrast, we found little evidence for either phenotypic or genetic correlation between our panel of adipocytokines and hormones in Africans. This difference may be attributed to the cytokines studied, which differed from the study by Nath et al. with IL-6 and IL-10 being the only cytokines in common. While all 13 adipocytokines we studied are secreted into the circulation and contribute to the pathophysiology of obesity and diabetes, they operate physiologically through different pathways (such as the gut-brain axis, adipogenesis, inflammation, and insulinotropic pathways) and on different organ systems that are important in metabolic traits [101–103]. This difference in physiological pathways may explain their lack of phenotypic and genetic correlation. Alternatively, the difference in population included between the study by Nath et al. [100] and our study—European compared with African in our study—may play a role. Nevertheless, the high complexity of the biology of circulating cytokines and hormones warrants consideration of potential networks across cytokines and populations.

## Conclusions

In conclusion, our findings contribute to the growing body of evidence on the genetic basis of obesity- and diabetes-related cytokines and hormones. The loci we identified and replicated for several cytokines and hormones provide insight into how these cytokines and hormones may influence cardiometabolic traits. The high number of African-specific loci detected emphasizes the need for GWAS in African-ancestry populations, as these loci could not have been detected in other populations. The strong effect of sex, BMI, and T2D status on GWAS findings highlights that stratified analyses facilitate the discovery of novel loci that have previously been undetected in combined analyses.

## Supplementary Information

**Additional file 1: Table S1**: Number of samples per cohort and per obesity-related cytokine and hormone. **Table S2**: Total number of genome-wide significant (*P*-value < 5×10-8) loci detected in the discovery cohort, number of novel loci, and number of loci replicated in African Americans per trait and per model. **Table S3**: Variants at genome-wide significance of *P*-value < 5×10-8 with low MAF (<5%) before and after removal of high leverage points based on Cook's distance. **Table S4**: Variants at genome-wide significance of *P*-value < 5×10-8 per adipocytokine/hormone for the meta-analysis of discovery and replication cohorts combined. **Table S5**: Genome-wide significant loci from base model analyses with moderate or strong evidence for colocalization with eQTL data from GTEx. **Table S6**: Variants at genome-wide significance of *P*-value < 5×10^-8^ per adipocytokine/hormone for men only. **Table S7**: Variants at genome-wide significance of *P*-value < 5×10^-8^ per adipocytokine/hormone for women only. **Table S8**: Variants at genome-wide significance of *P*-value < 5×10^-8^ per adipocytokine/hormone for lean (BMI < 25.0 kg/m^2^) individuals only. **Table S9**: Variants at genome-wide significance of *P*-value < 5×10^-8^ per adipocytokine/hormone for overweight (BMI ≥ 25.0 kg/m^2^) individuals only. **Table S10**: Variants at genome-wide significance of *P*-value < 5×10^-8^ per adipocytokine/hormone for T2D controls only. **Table S11**: Variants at genome-wide significance of *P*-value < 5×10^-8^ per adipocytokine/hormone for T2D cases only. **Table S12**: Canonical pathways for genes annotated to genome-wide significant and suggestive loci in the base model

**Additional file 2. Fig S1: **Principal Components 1 and 2 in continental Africans from the AADM study (A) and for the AADM study combined with 1000 Genomes Project populations (B), colored by ethnic group.** Fig S2: **Regional plots of all loci detected in the base model (P-value < 5x10-8) and all replicated loci from stratified models.** Fig S3: **Manhattan plots for meta-analysis of discovery and replication cohorts combined for all 13 obesity- and diabetes-related cytokines and hormones. **Fig S4: **Miami plots for sex-stratified analyses for all 13 obesity- and diabetes-related cytokines and hormones.** Fig S5: **Miami plots for analyses stratified on BMI status for all 13 obesity- and diabetes-related cytokines and hormones.** Fig S6: **Miami plots for T2D status-stratified analyses for all 13 obesity- and diabetes-related cytokines and hormones.

## Data Availability

The AADM and HUFS datasets used and/or analyzed in the current study are available from the corresponding author upon request as permitted by the IRB approval and signed informed consent. These data are not available through a repository due to the consent obtained which does not grant permission for deposition. The data for the other four cohorts were accessed through dbGaP: ARIC [36, 37, 104], CFS [34, 35, 105], JHS [38, 106], and MESA [33, 107].
