# Supplementary material for: Genome-wide analyses of multiple obesity-related cytokines and hormones informs biology of cardiometabolic traits
Source: Genome Med. 2021 Oct 7;13:156. doi: 10.1186/s13073-021-00971-2 (PMC8499470; doi:10.1186/s13073-021-00971-2)
Supplement: Supplementary file 2 — Additional file 2. Fig S1: Principal Components 1 and 2 in continental Africans from the AADM study (A) and for the AADM study combined with 1000 Genomes Project populations (B), colored by ethnic group. Fig S2: Regional plots of all loci detected in the base model (P-value < 5x10^-8^) and all replicated loci from stratified models. Fig S3: Manhattan plots for meta-analysis of discovery and replication cohorts combined for all 13 obesity- and diabetes-related cytokines and hormones. Fig S4: Miami plots for sex-stratified analyses for all 13 obesity- and diabetes-related cytokines and hormones. Fig S5: Miami plots for analyses stratified on BMI status for all 13 obesity- and diabetes-related cytokines and hormones. Fig S6: Miami plots for T2D status-stratified analyses for all 13 obesity- and diabetes-related cytokines and hormones. [file 13073_2021_971_MOESM2_ESM.pdf]

Additional File 2

Fig S1: Principal Components 1 and 2 in continental Africans from the AADM study (A) and for the AADM study combined with 1000 Genomes Project populations (B), colored by ethnic group

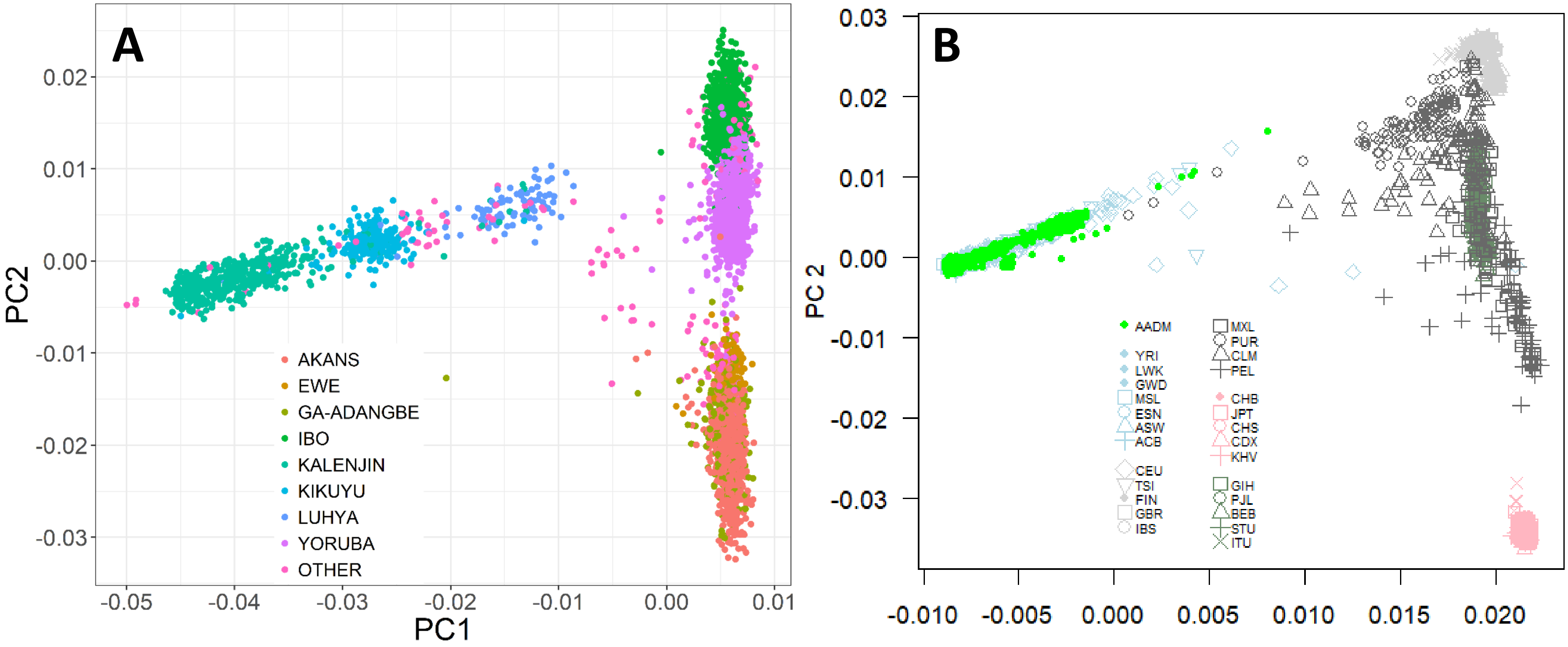

YRI - Yoruba in Ibadan, Nigeria; LWK - Luhya in Webuye, Kenya; GWD - Gambian in Western Divisions in the Gambia; MSL - Mende in Sierra Leone; ESN - Esan in Nigeria; ASW - Americans of African Ancestry in SW USA; ACB - African Caribbeans in Barbados; CEU - Utah Residents (CEPH) with Northern and Western European Ancestry; TSI - Toscani in Italia; FIN - Finnish in Finland; GBR - British in England and Scotland; IBS - Iberian Population in Spain; MXL - Mexican Ancestry from Los Angeles USA; PUR - Puerto Ricans from Puerto Rico; CLM - Colombians from Medellin, Colombia; PEL - Peruvians from Lima, Peru; CHB - Han Chinese in Beijing, China; JPT - Japanese in Tokyo, Japan; CHS - Southern Han Chinese; CDX - Chinese Dai in Xishuangbanna, China; KHV - Kinh in Ho Chi Minh City, Vietnam; GIH - Gujarati Indian from Houston, Texas; PJL - Punjabi from Lahore, Pakistan; BEB - Bengali from Bangladesh; STU - Sri Lankan Tamil from the UK; ITU - Indian Telugu from the UK.

Fig S2: Regional plots of all loci detected in the base model (P-value < $5x10^{-8}$) and all replicated loci from stratified models.

*Linkage Disequilibrium (LD) information shown was derived from the 1000 Genomes Project AFR reference population. For loci that did not have 1000 Genomes Project AFR reference LD data available (indicated with *), LD data were derived from the AADM study.*

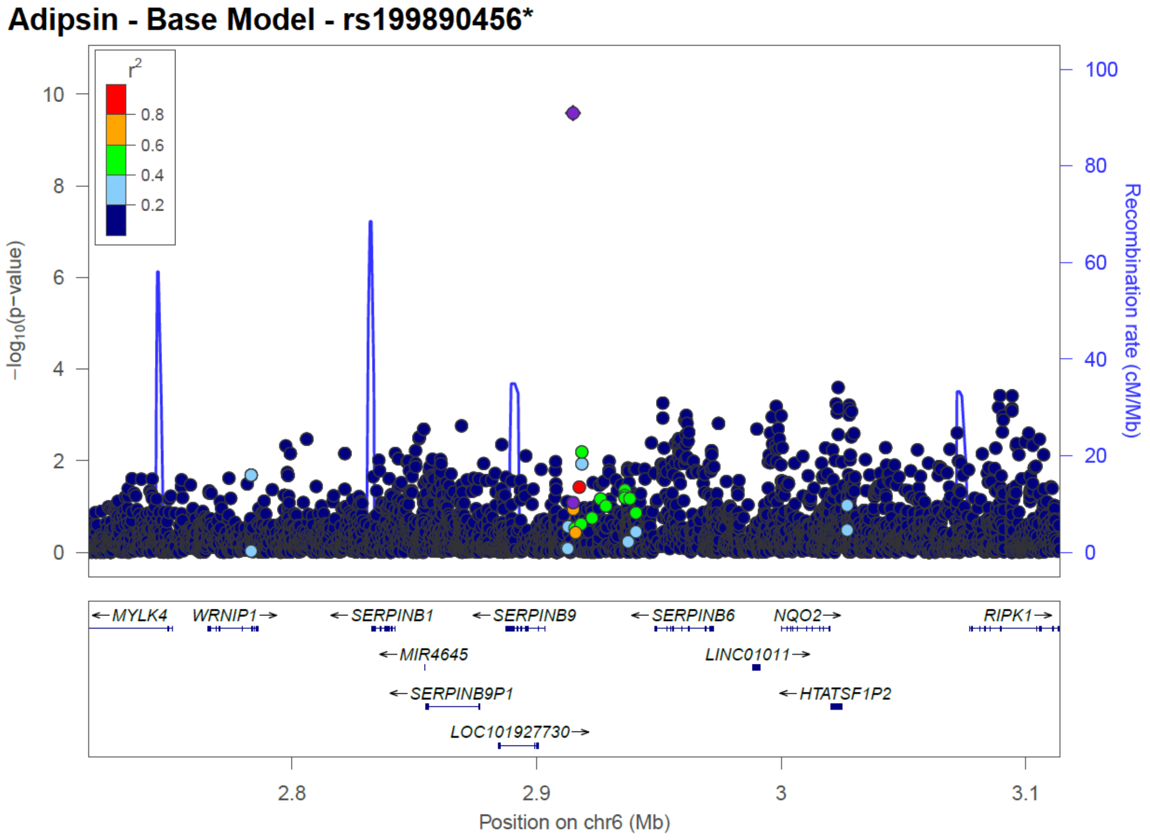

Adipsin - Base Model - rs201751833

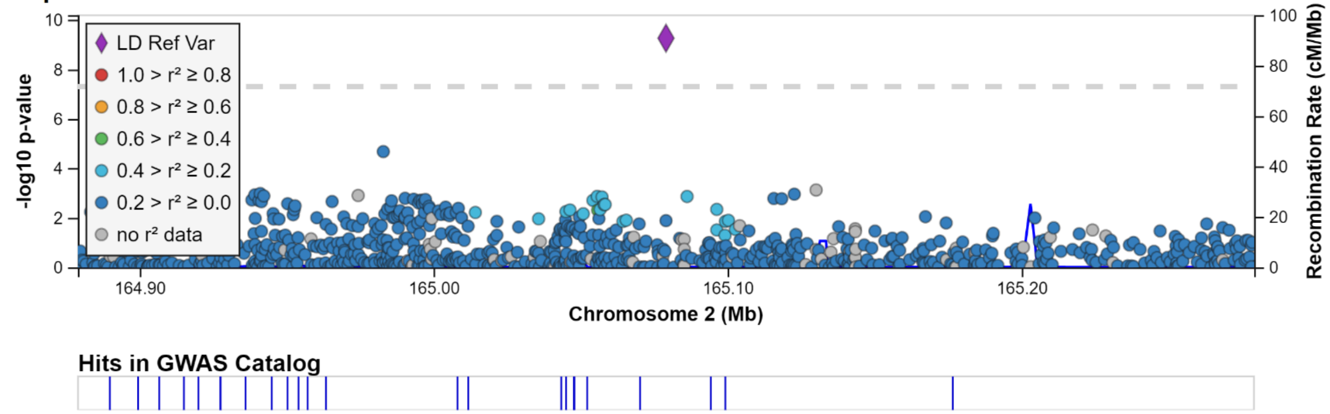

Adipsin - Base Model - rs1469952

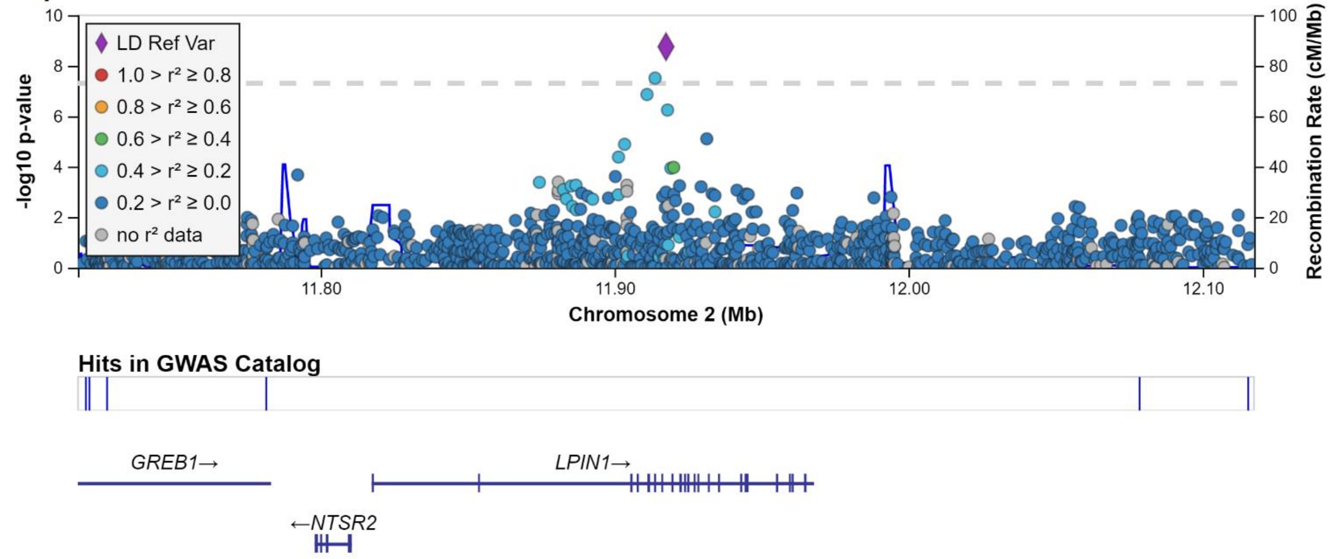

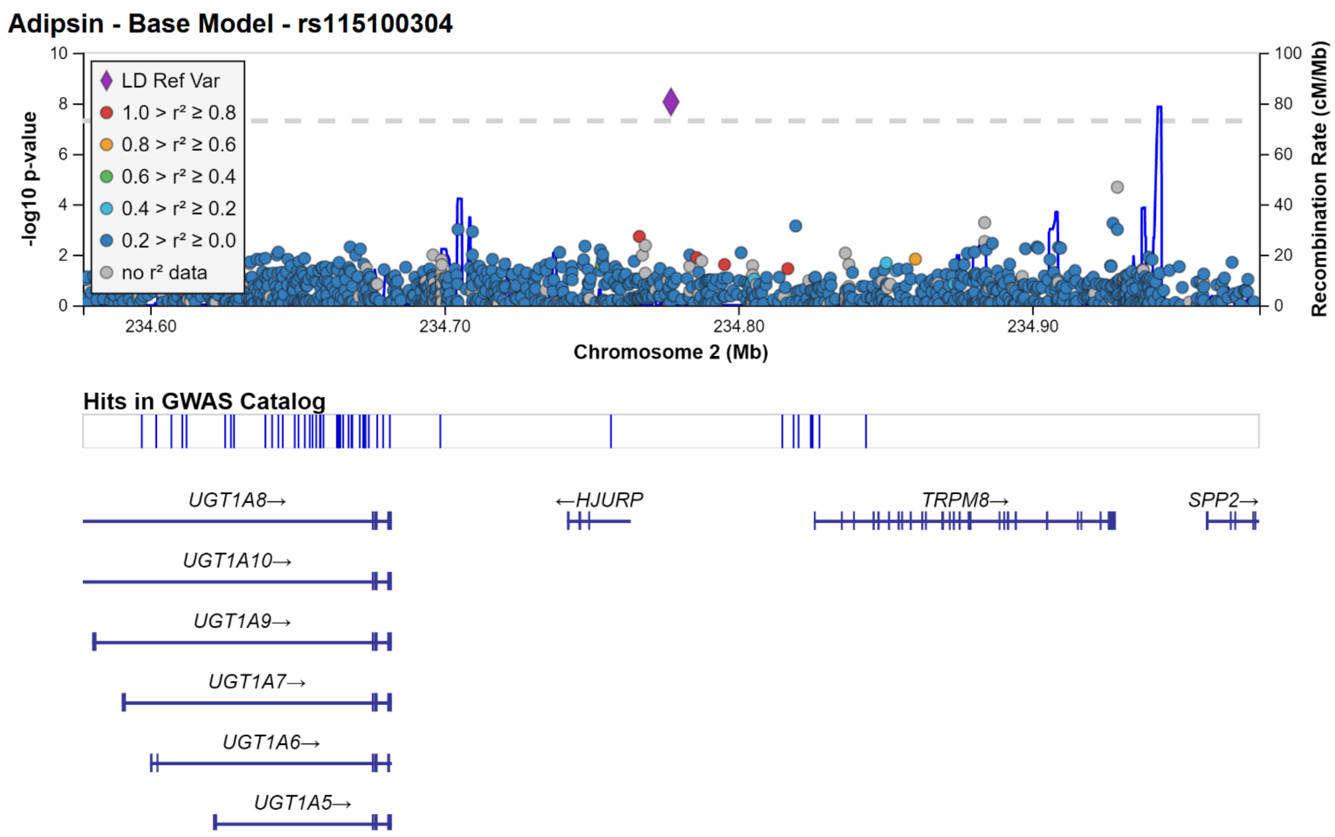
Adipsin - Base Model - rs115100304
LD Ref Var
1.0 > r² ≥ 0.8
0.8 > r² ≥ 0.6
0.6 > r² ≥ 0.4
0.4 > r² ≥ 0.2
0.2 > r² ≥ 0.0
no r² data
-log10 p-value
Recombination Rate (cM/Mb)
234.60
234.70
234.80
234.90
Chromosome 2 (Mb)
Hits in GWAS Catalog
UGT1A8→
UGT1A10→
UGT1A9→
UGT1A7→
UGT1A6→
UGT1A5→
←HJURP
TRPM8→
SPP2→

Adipsin - Base Model - rs34061523*

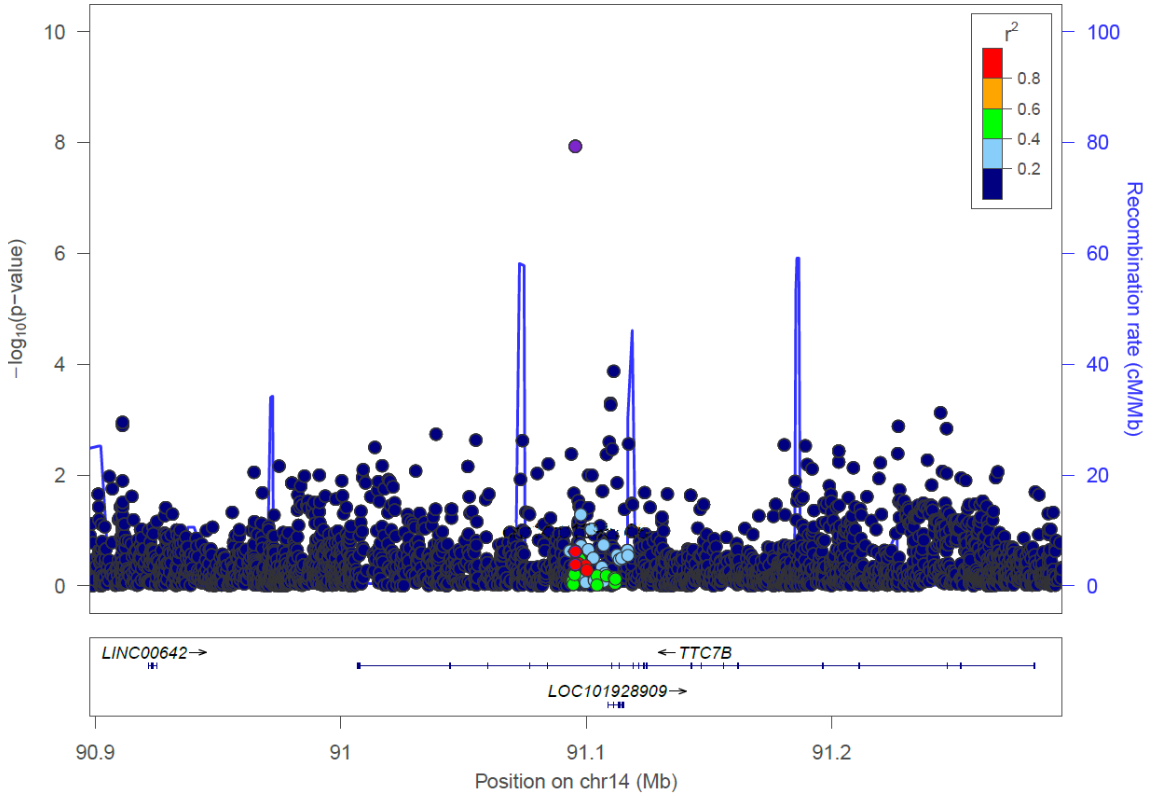

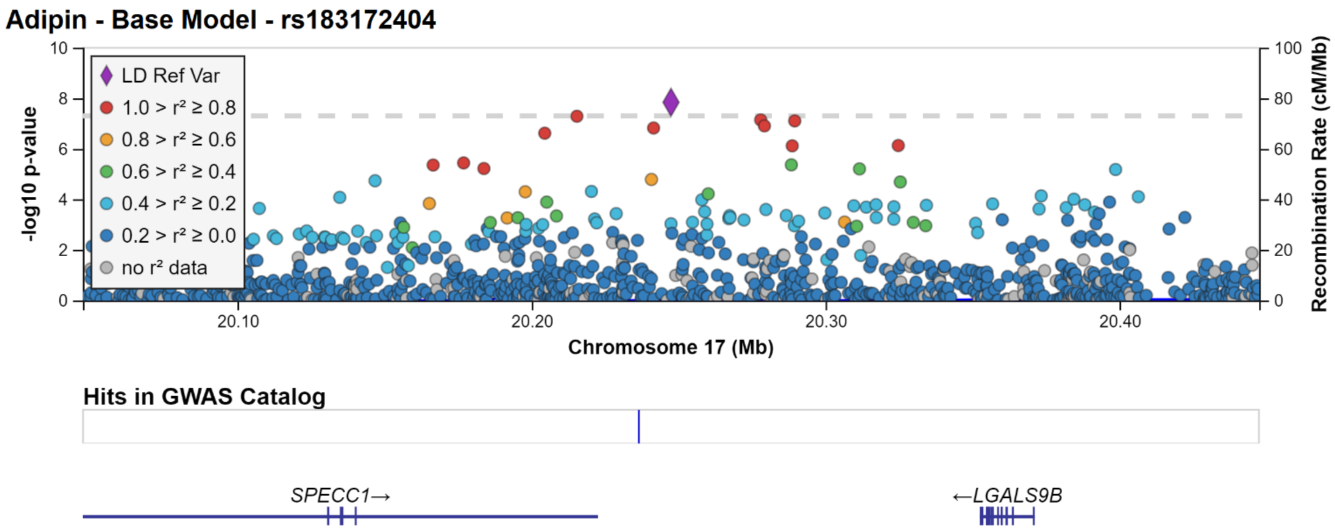

Adipin - Base Model - rs183172404
LD Ref Var
1.0 > r² ≥ 0.8
0.8 > r² ≥ 0.6
0.6 > r² ≥ 0.4
0.4 > r² ≥ 0.2
0.2 > r² ≥ 0.0
no r² data
-log10 p-value
Recombination Rate (cM/Mb)
Chromosome 17 (Mb)
20.10
20.20
20.30
20.40
Hits in GWAS Catalog
SPECC1→
←LGALS9B

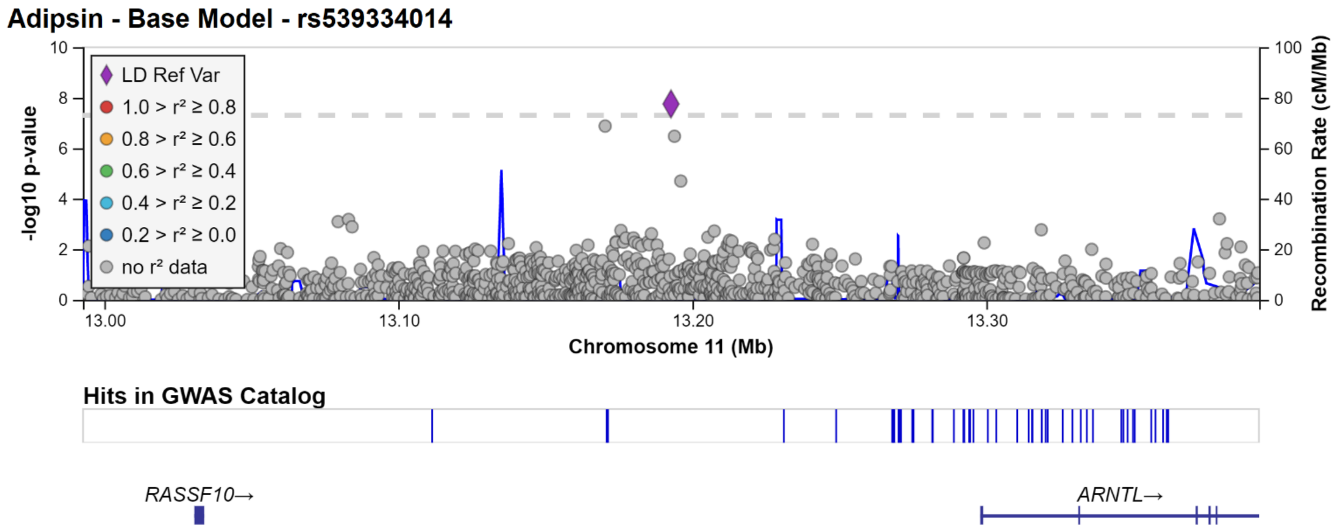

Adipsin - Base Model - rs539334014
LD Ref Var
1.0 > r² ≥ 0.8
0.8 > r² ≥ 0.6
0.6 > r² ≥ 0.4
0.4 > r² ≥ 0.2
0.2 > r² ≥ 0.0
no r² data
-log10 p-value
Recombination Rate (cM/Mb)
Chromosome 11 (Mb)
13.00
13.10
13.20
13.30
Hits in GWAS Catalog
RASSF10→
ARNTL→

Adipsin - Base Model - rs79750258*

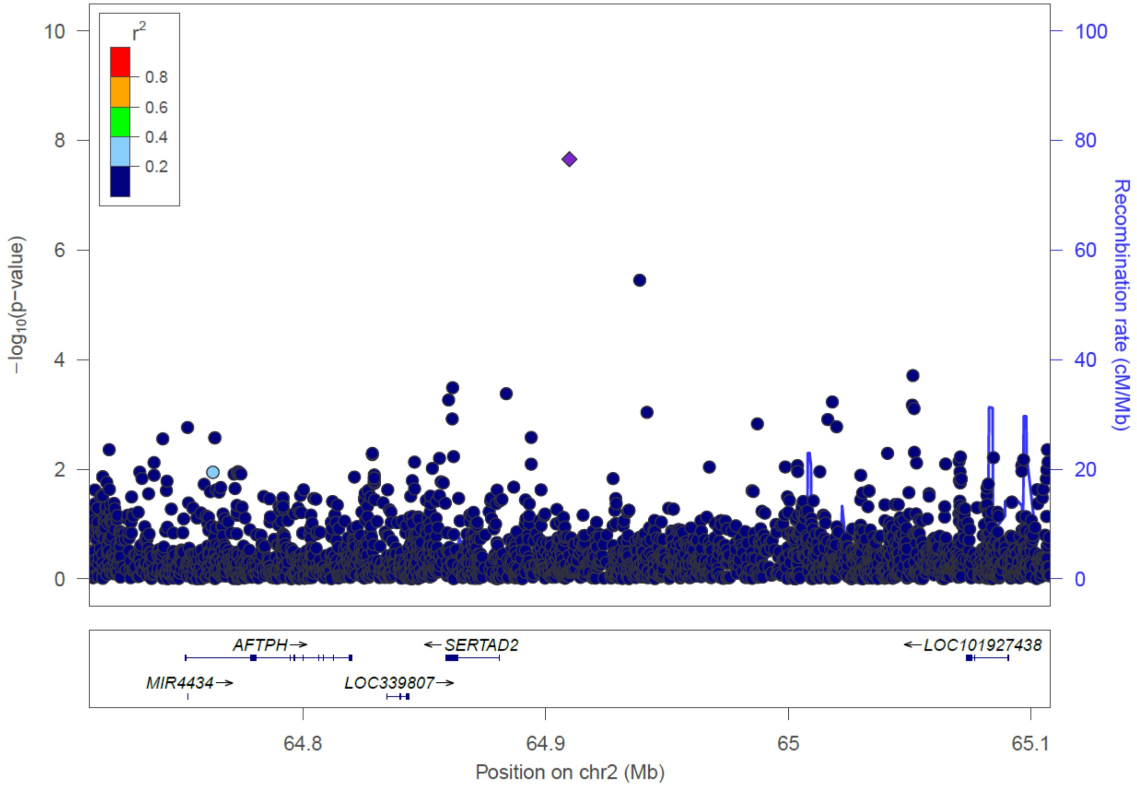

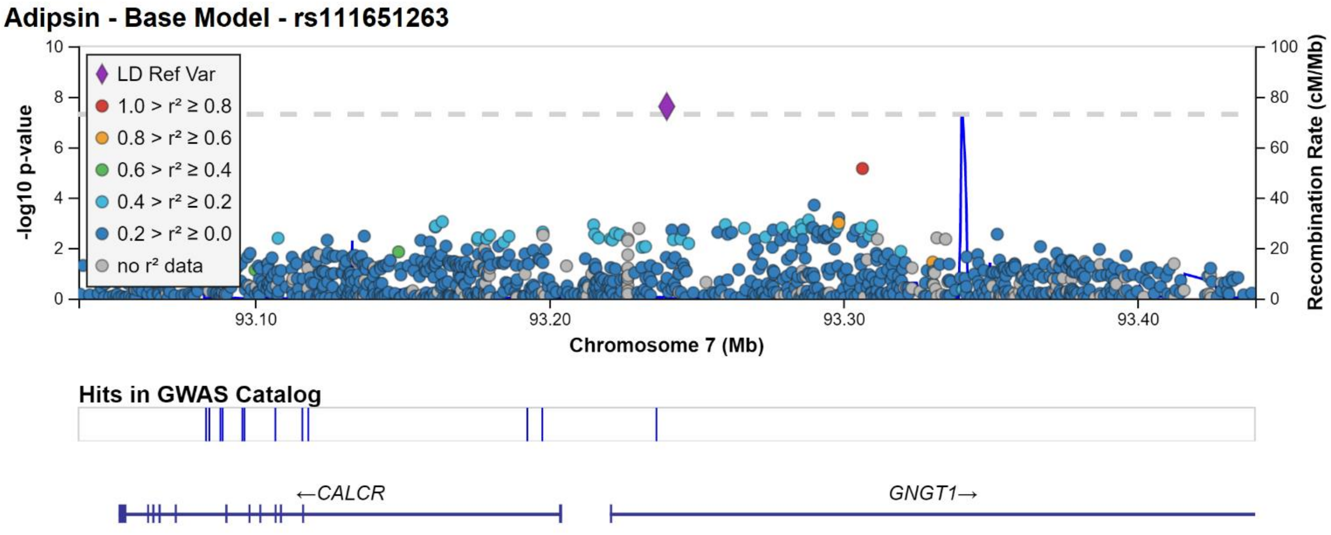

Adipsin - Base Model - rs111651263
LD Ref Var
1.0 > r² ≥ 0.8
0.8 > r² ≥ 0.6
0.6 > r² ≥ 0.4
0.4 > r² ≥ 0.2
0.2 > r² ≥ 0.0
no r² data
-log10 p-value
Recombination Rate (cM/Mb)
Chromosome 7 (Mb)
93.10
93.20
93.30
93.40
Hits in GWAS Catalog
←CALCR
GNGT1→

Adipsin - Base Model - rs79024755*

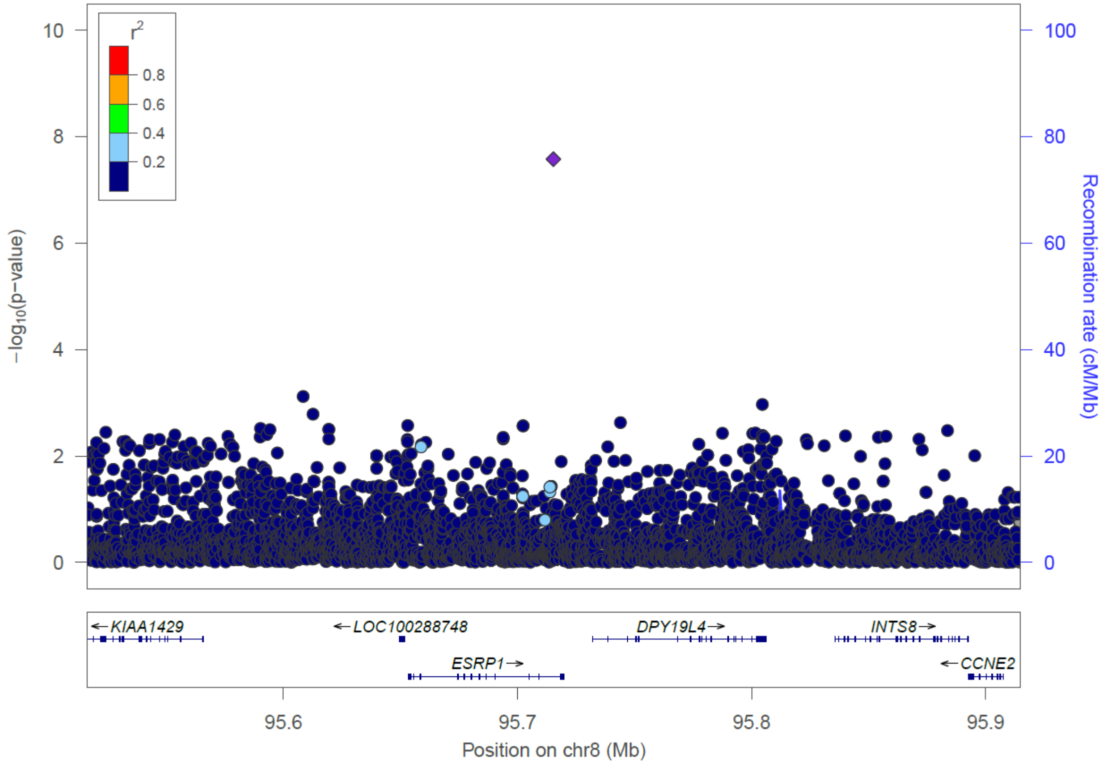

Adipsin - Base Model - rs113529034*

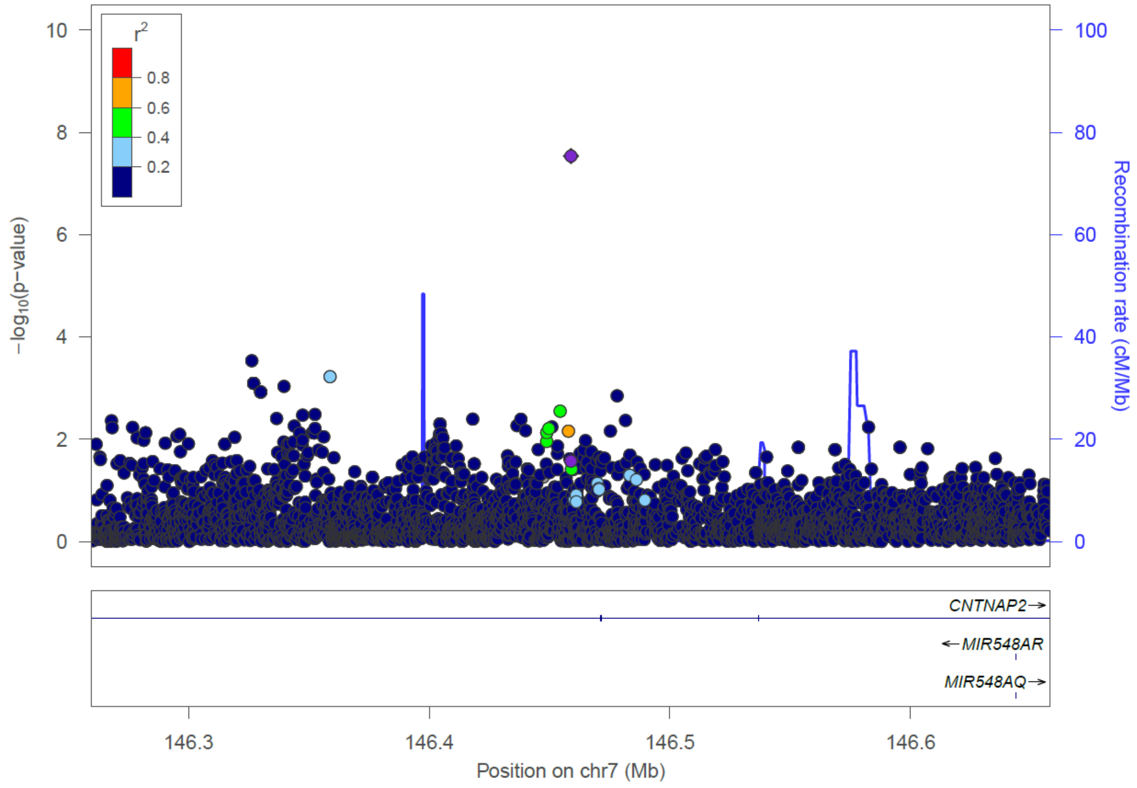

Adipsin - Base Model - rs180913374*

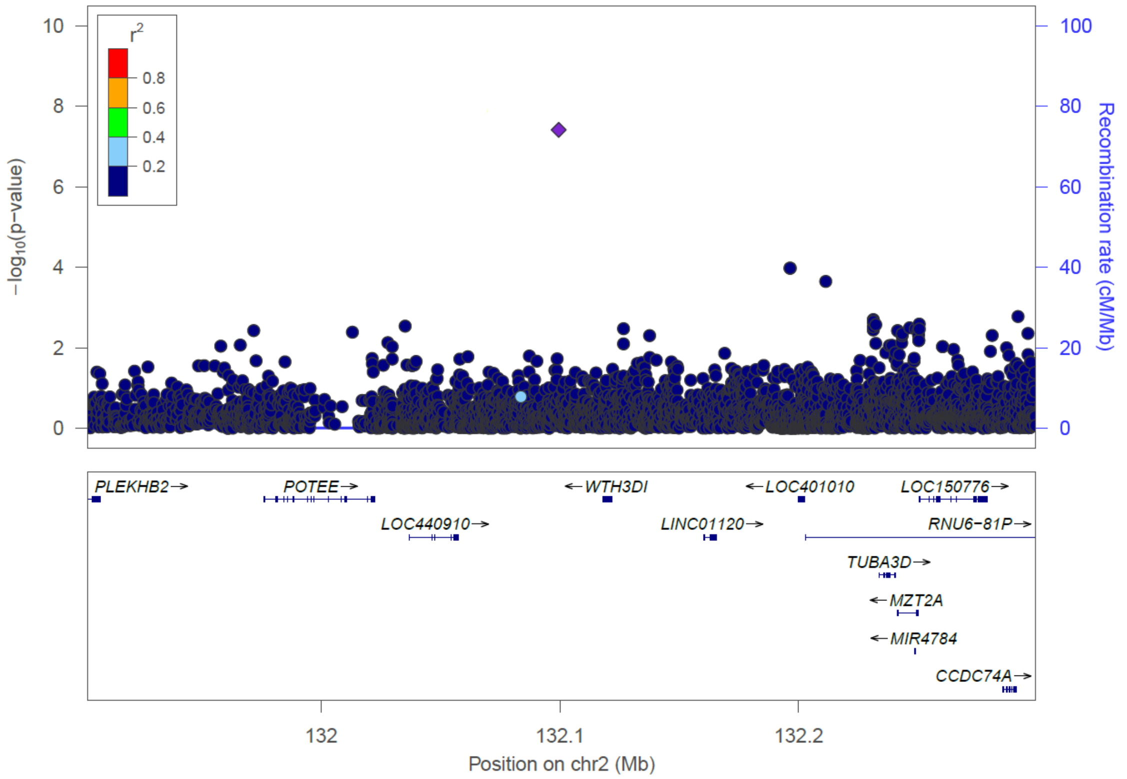

Adipsin - Base Model - rs145662005

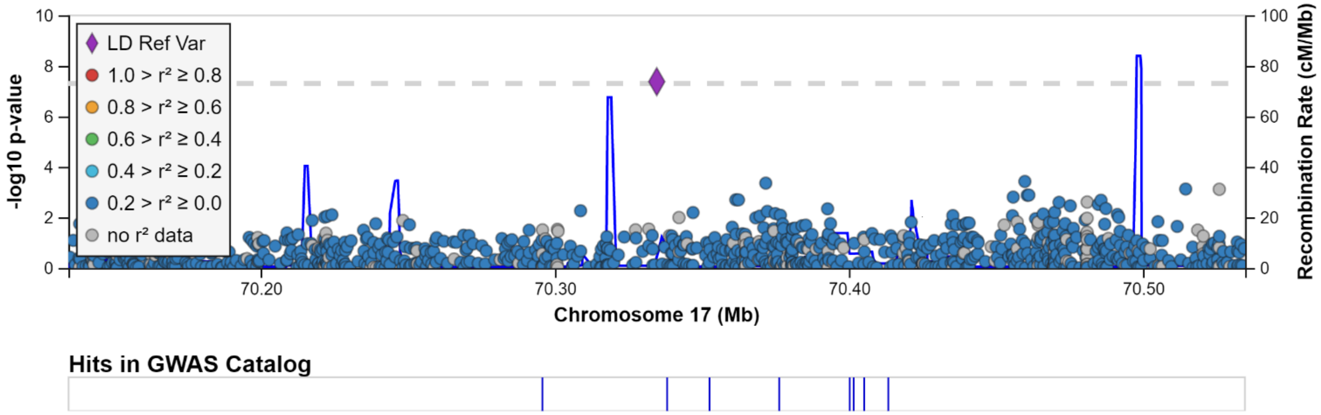

Leptin - Base Model - rs28954105

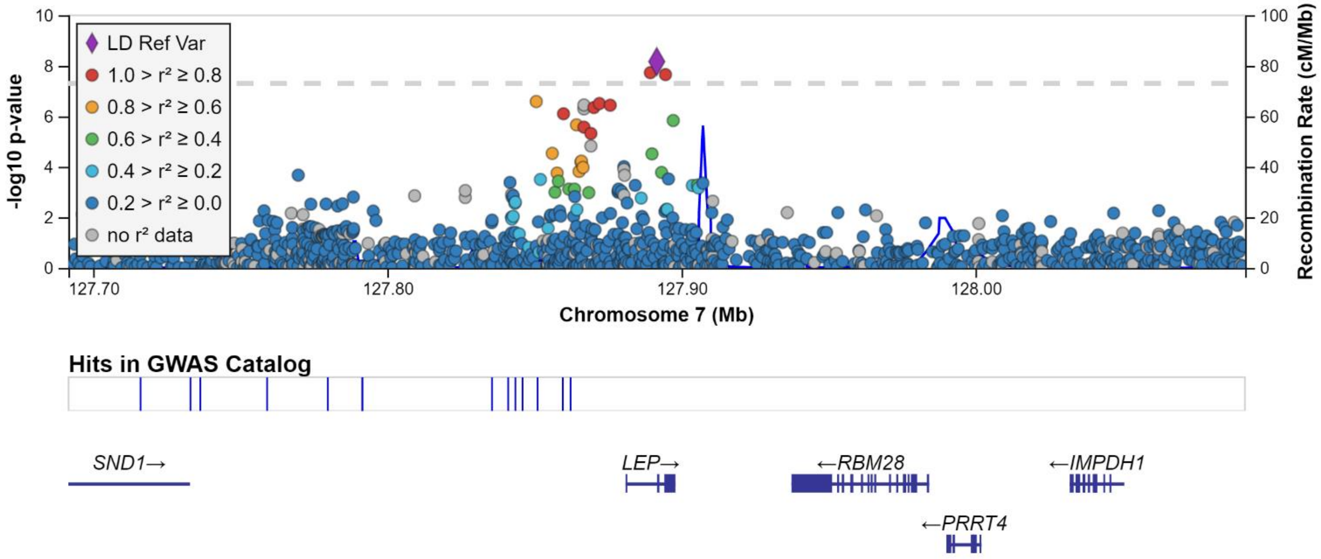

Leptin - Base Model - rs113453972

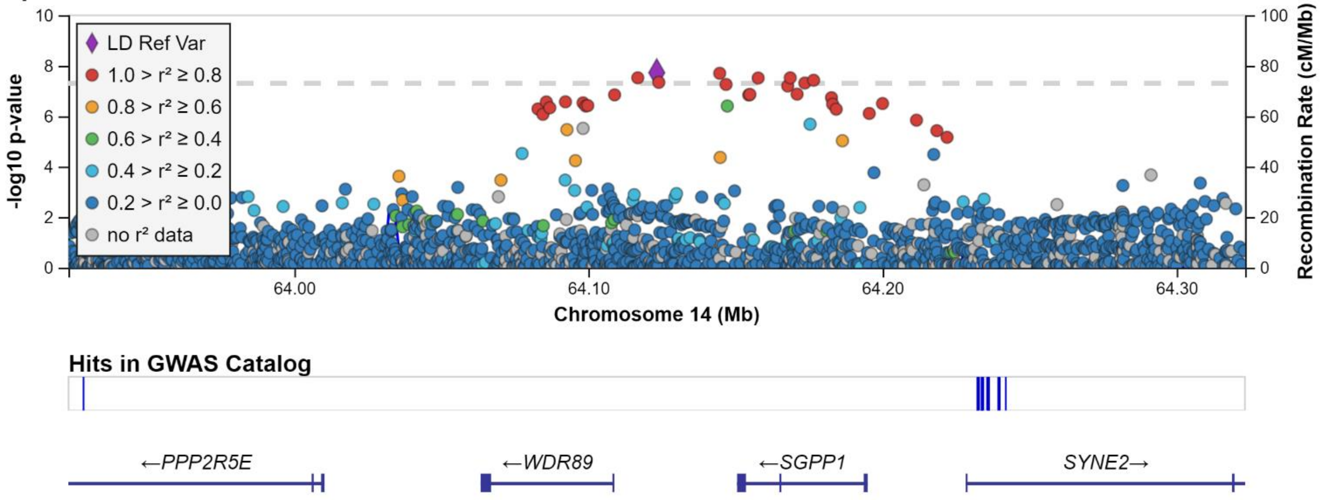

Leptin - Base Model - rs61258383

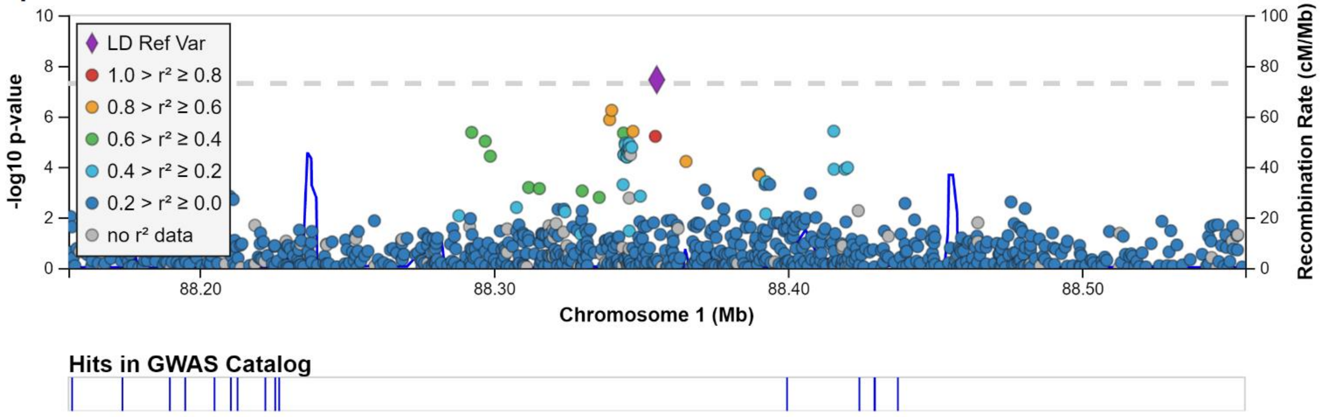

Leptin - Base Model - rs9894577

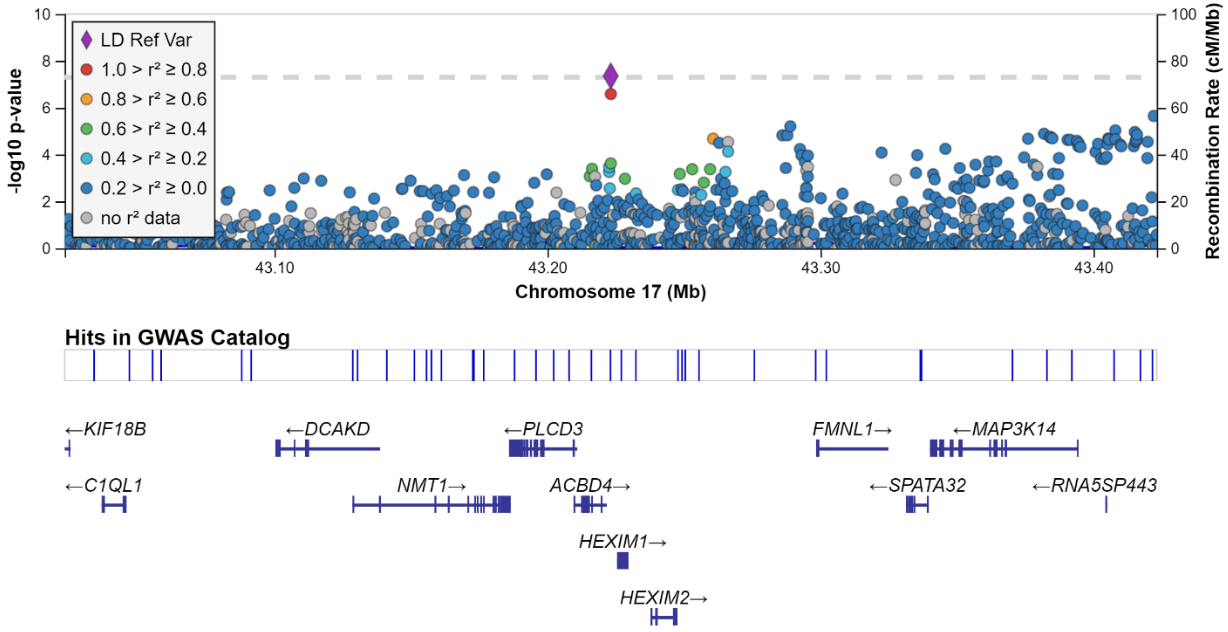

GIP - Base Model - rs12028926

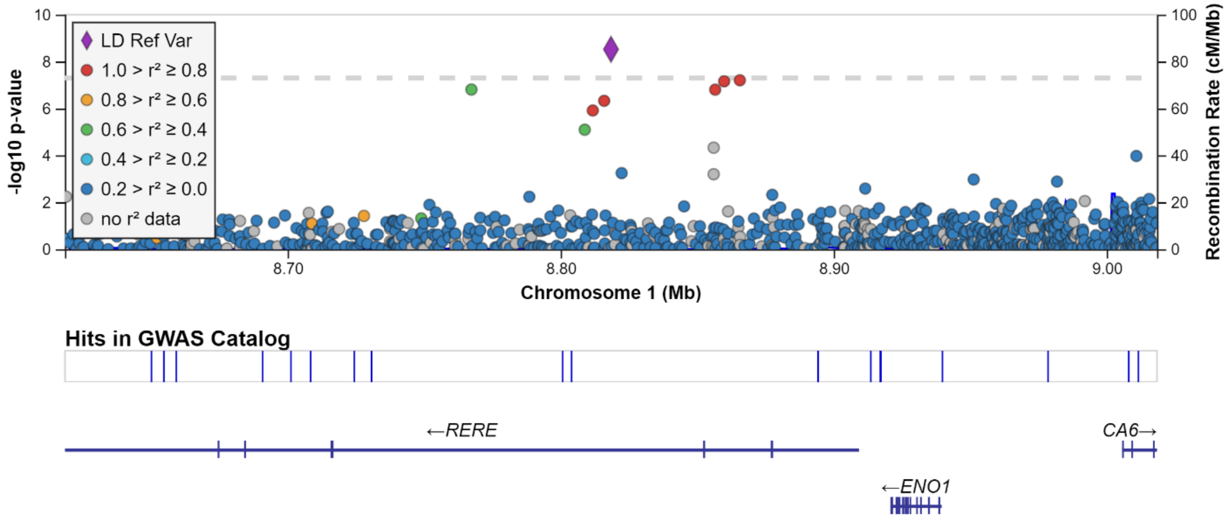

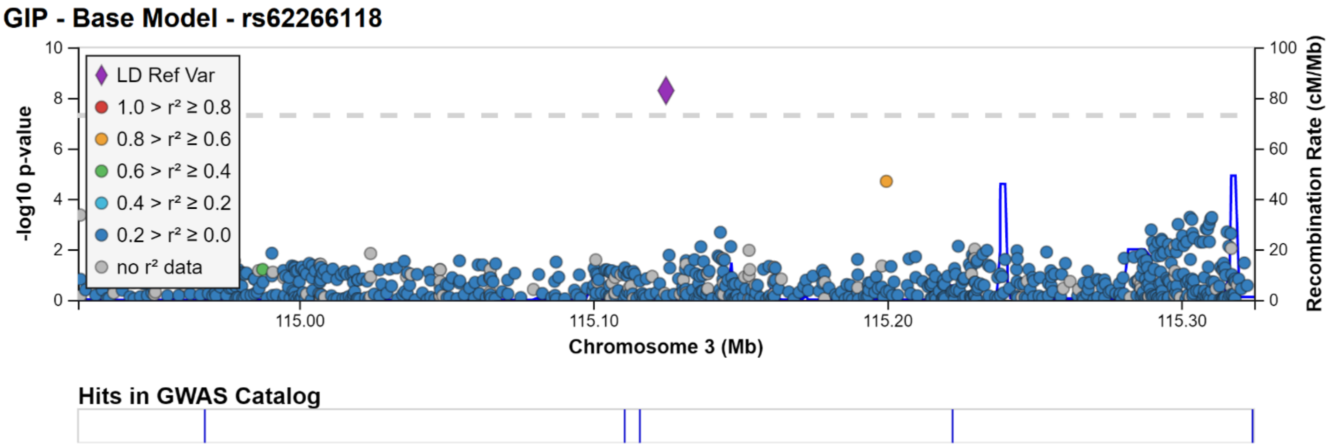
GIP - Base Model - rs62266118
LD Ref Var
1.0 > r² ≥ 0.8
0.8 > r² ≥ 0.6
0.6 > r² ≥ 0.4
0.4 > r² ≥ 0.2
0.2 > r² ≥ 0.0
no r² data
-log10 p-value
Recombination Rate (cM/Mb)
115.00
115.10
115.20
115.30
Chromosome 3 (Mb)
Hits in GWAS Catalog

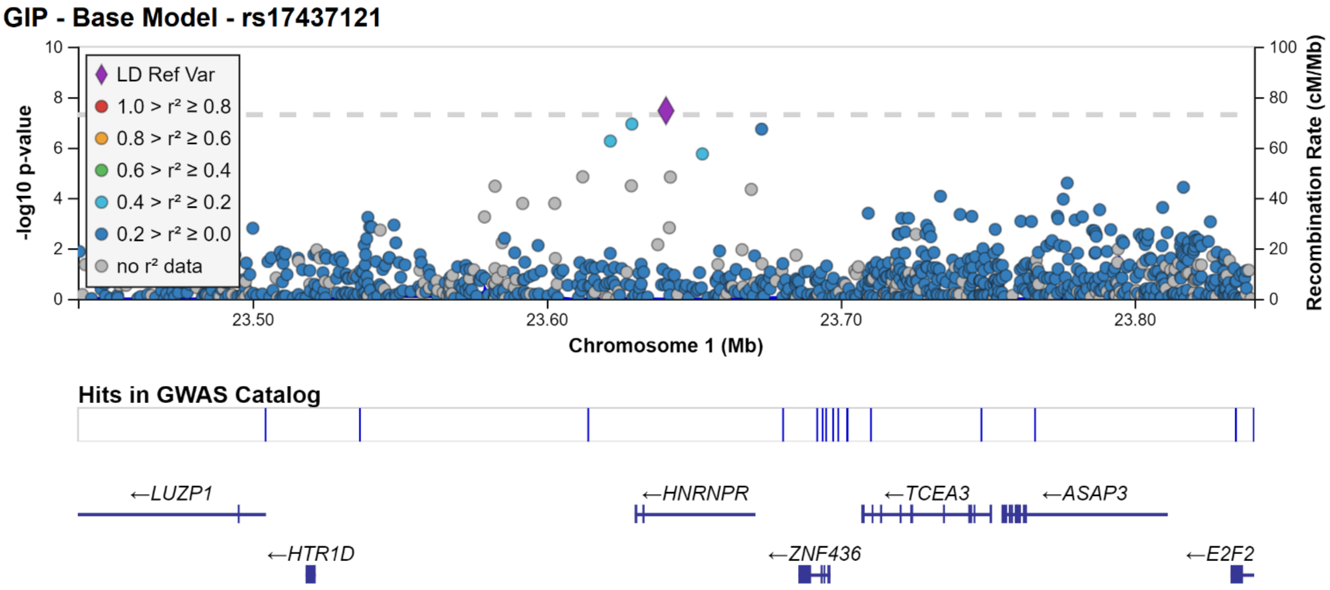
GIP - Base Model - rs17437121
LD Ref Var
1.0 > r² ≥ 0.8
0.8 > r² ≥ 0.6
0.6 > r² ≥ 0.4
0.4 > r² ≥ 0.2
0.2 > r² ≥ 0.0
no r² data
-log10 p-value
Recombination Rate (cM/Mb)
23.50
23.60
23.70
23.80
Chromosome 1 (Mb)
Hits in GWAS Catalog
←LUZP1
←HNRNPR
←TCEA3
←ASAP3
←HTR1D
←ZNF436
←E2F2

GIP - Base Model - rs17335662

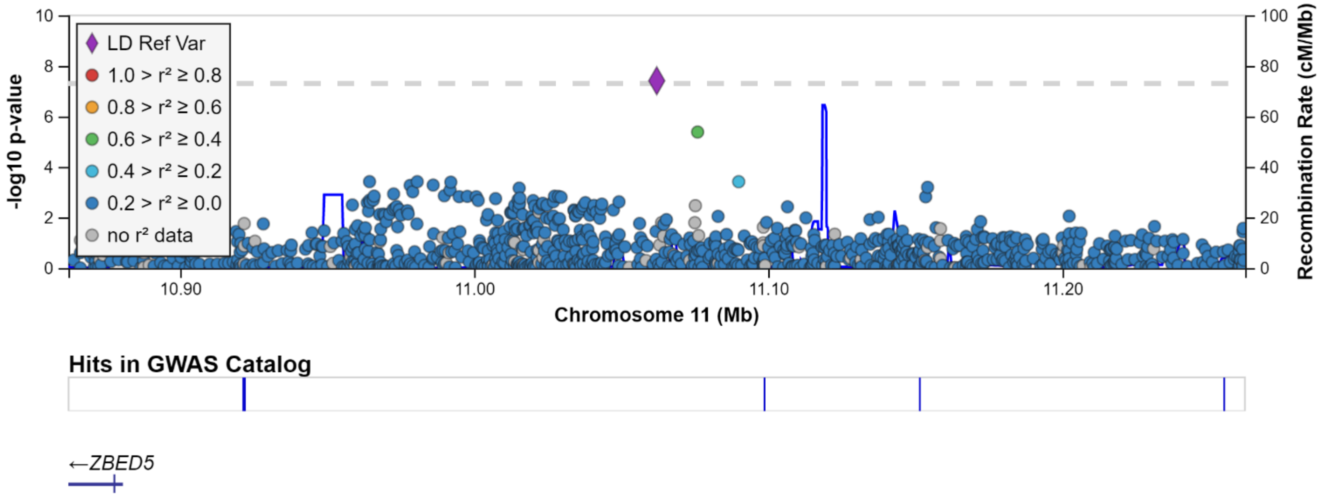

GIP - Base Model - rs182578321

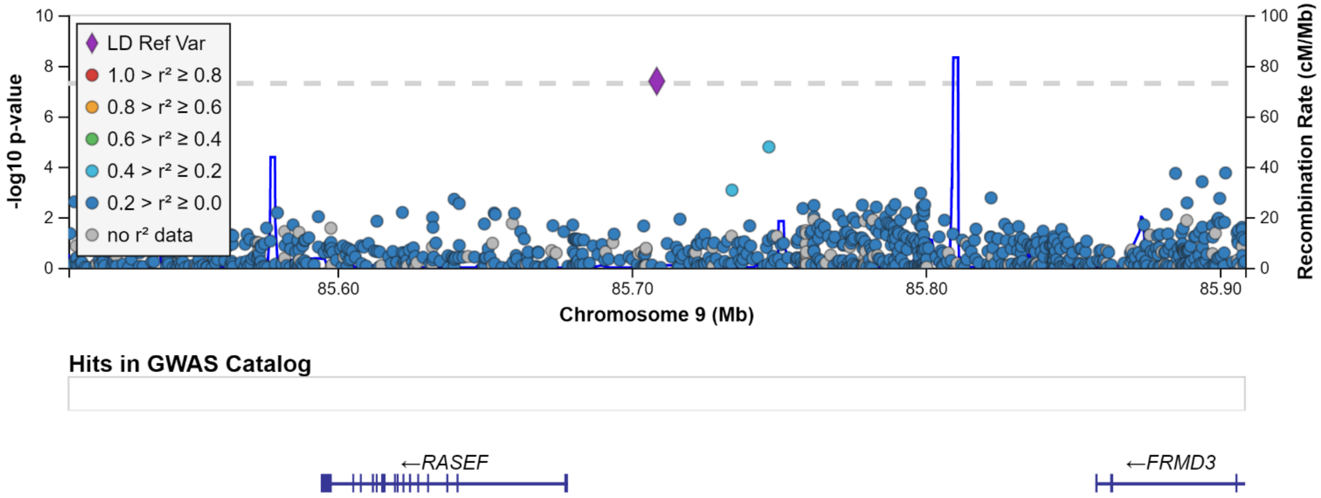

GIP - Base Model - rs988623129*

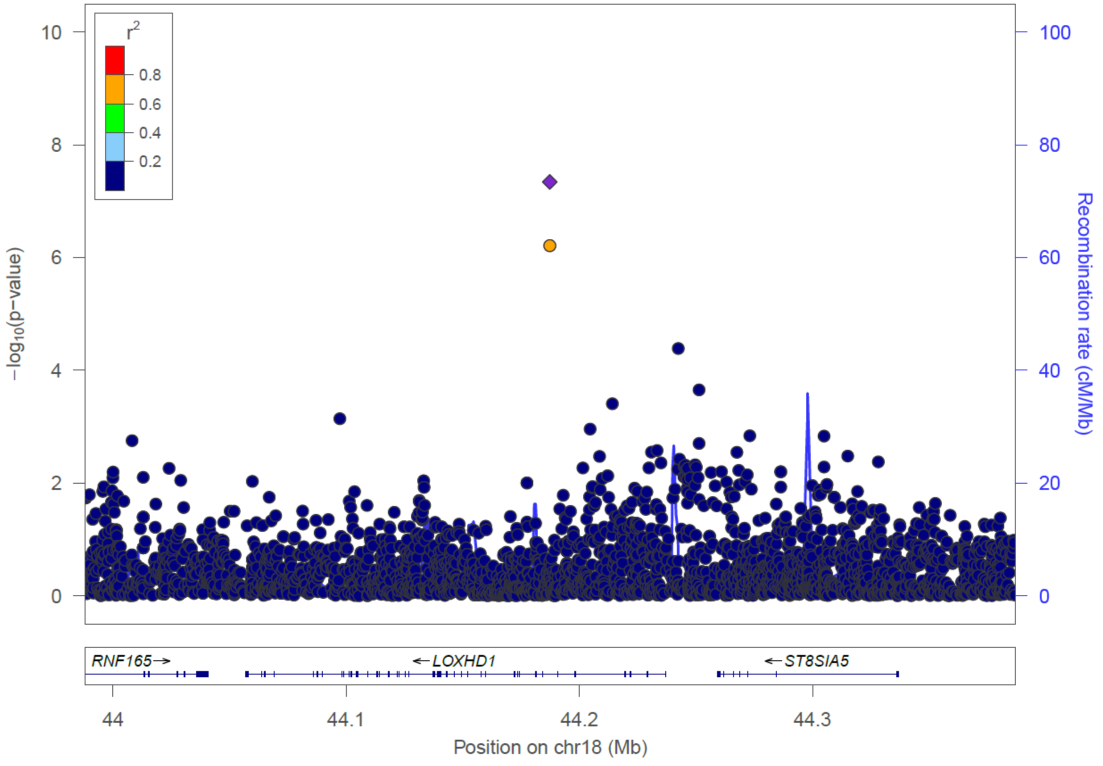

GLP-1 - Base Model - rs1355371392*

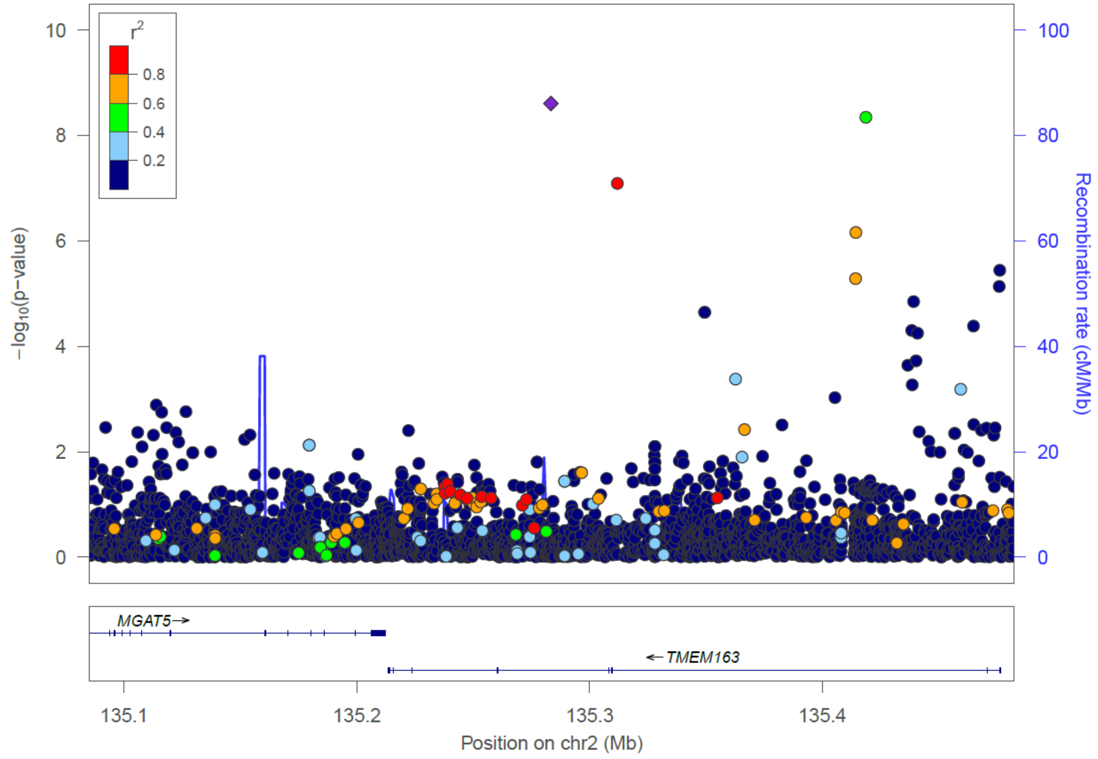

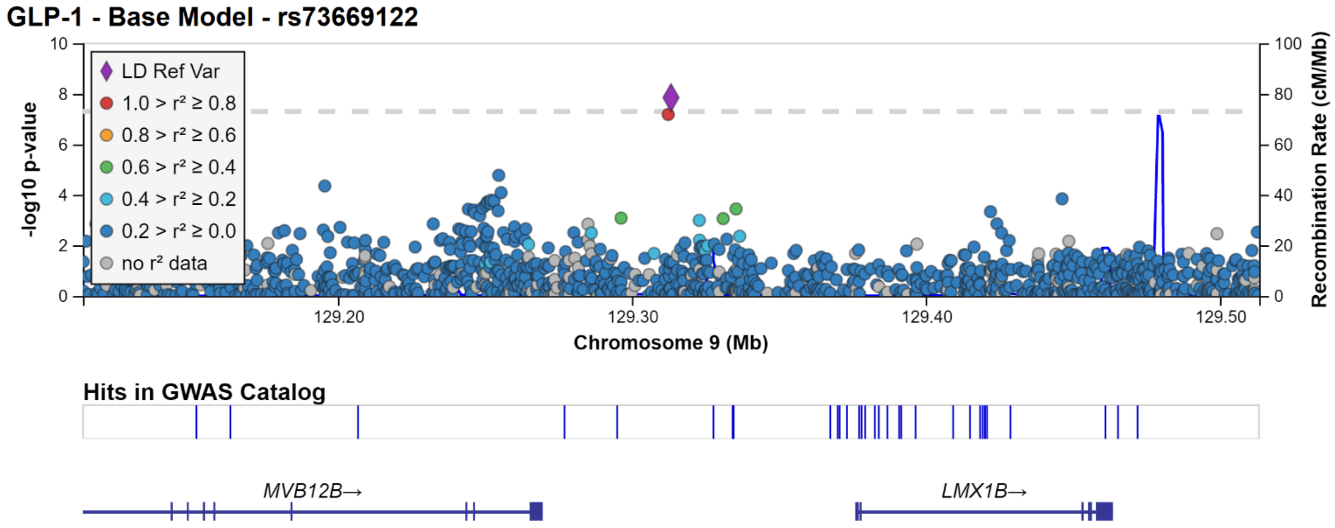
GLP-1 - Base Model - rs73669122
LD Ref Var
1.0 > r² ≥ 0.8
0.8 > r² ≥ 0.6
0.6 > r² ≥ 0.4
0.4 > r² ≥ 0.2
0.2 > r² ≥ 0.0
no r² data
-log10 p-value
Recombination Rate (cM/Mb)
Chromosome 9 (Mb)
129.20
129.30
129.40
129.50
Hits in GWAS Catalog
MVB12B→
LMX1B→

GLP-1 - Base Model - rs1450571579*

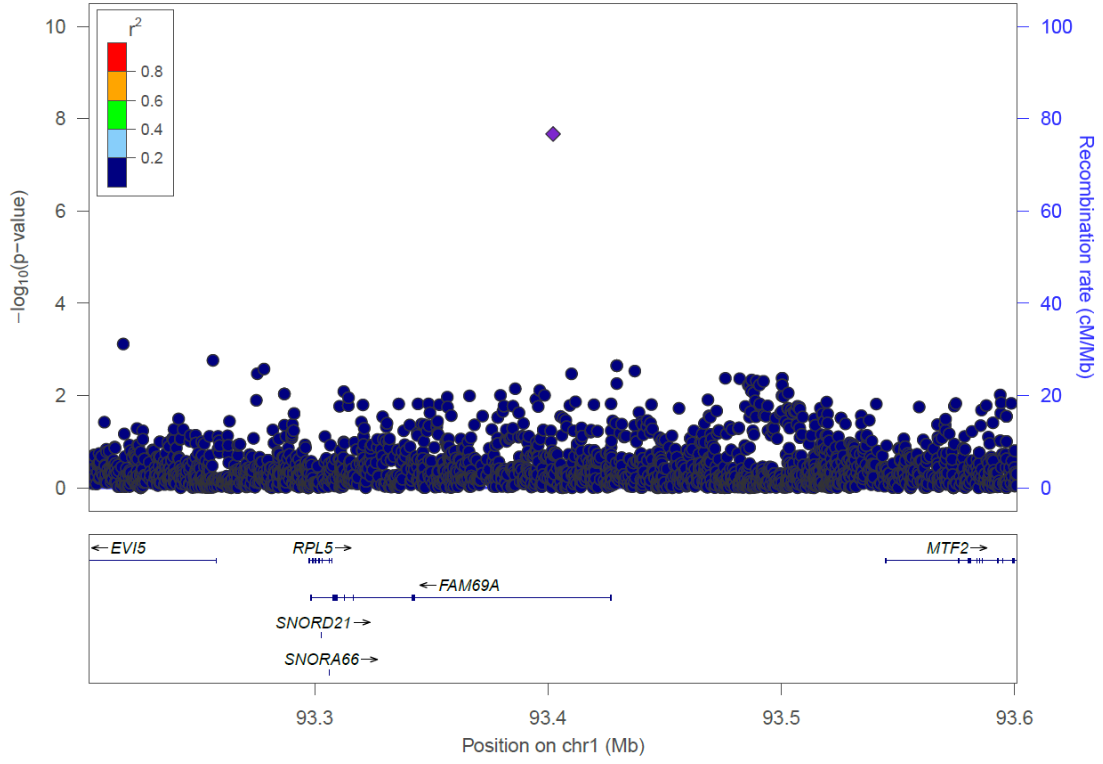

GLP-1 - Base Model - rs1445210817*

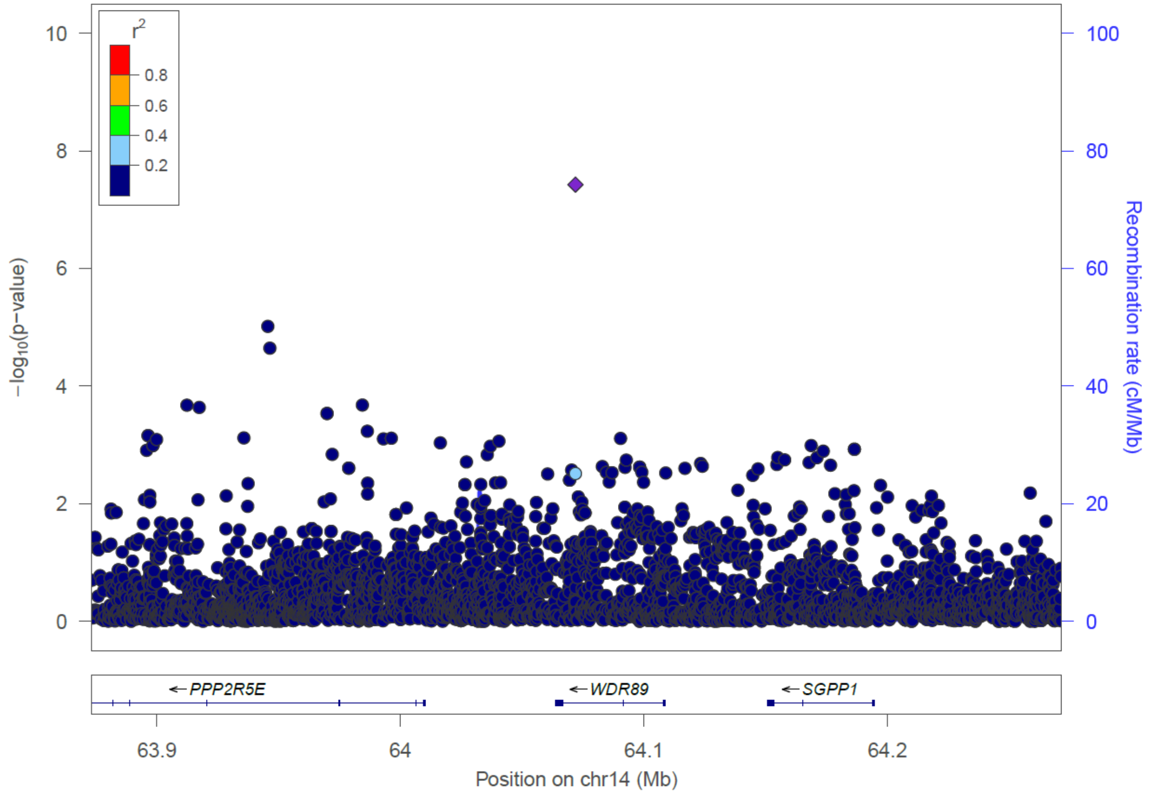

GLP-1 - Base Model - rs372701742*

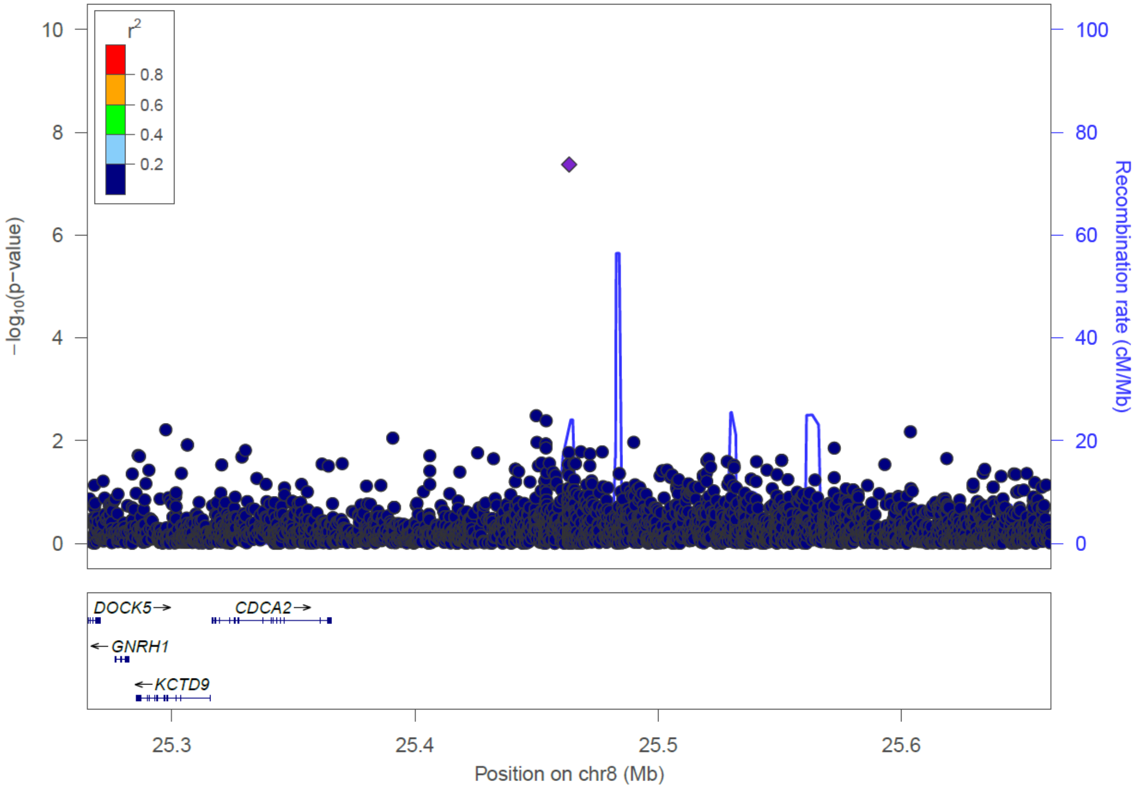

PAI-1 - Base Model - rs113421429

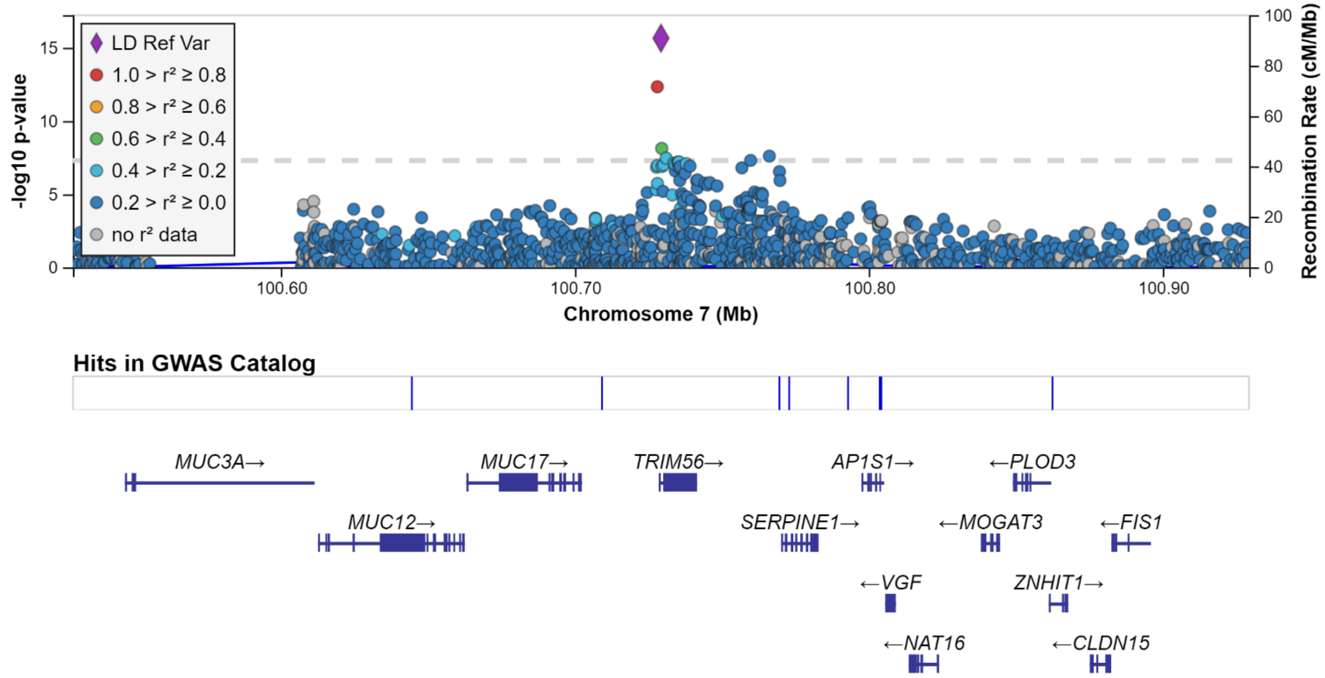

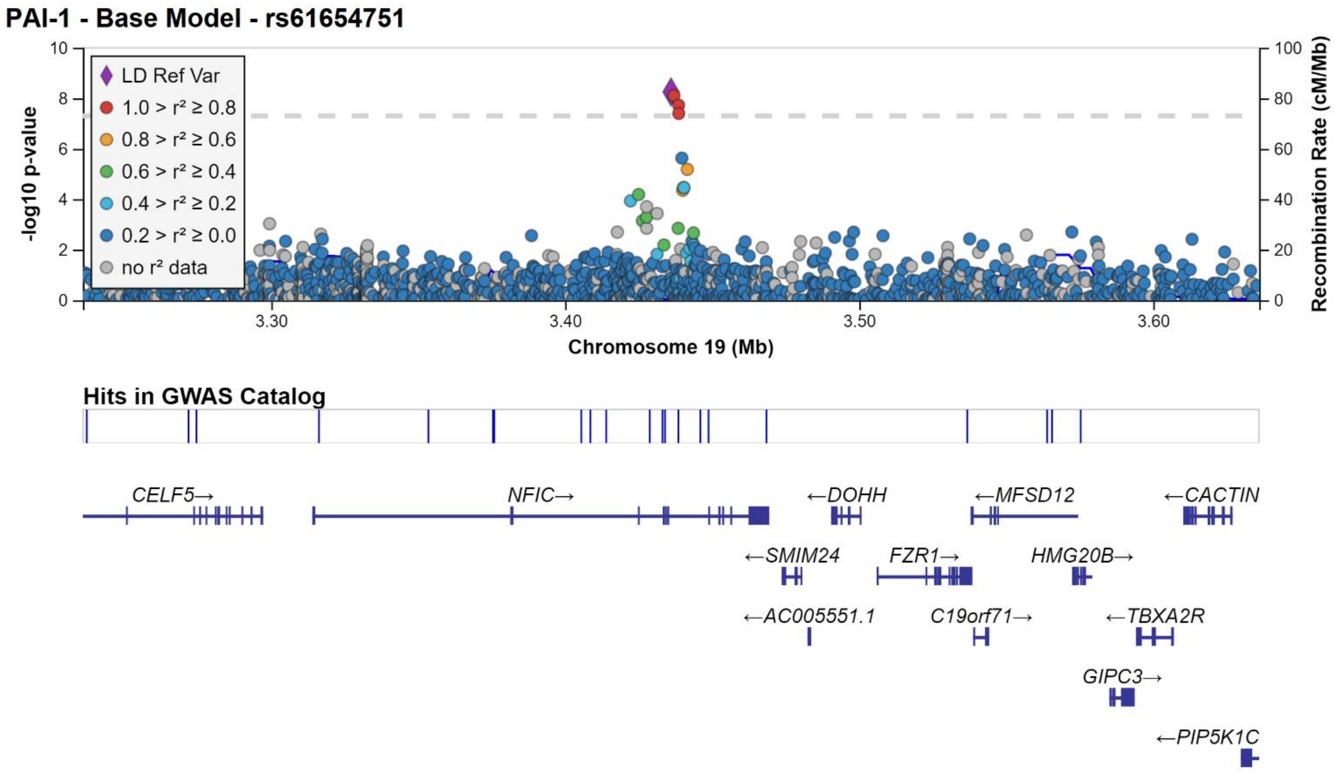
PAI-1 - Base Model - rs61654751
LD Ref Var
1.0 > r² ≥ 0.8
0.8 > r² ≥ 0.6
0.6 > r² ≥ 0.4
0.4 > r² ≥ 0.2
0.2 > r² ≥ 0.0
no r² data
-log10 p-value
Recombination Rate (cM/Mb)
Chromosome 19 (Mb)
Hits in GWAS Catalog
CELF5→
NFIC→
←DOHH
←MFSD12
←CACTIN
←SMIM24
FZR1→
HMG20B→
←AC005551.1
C19orf71→
←TBXA2R
GIPC3→
←PIP5K1C

PAI-1 - Base Model - rs2496687

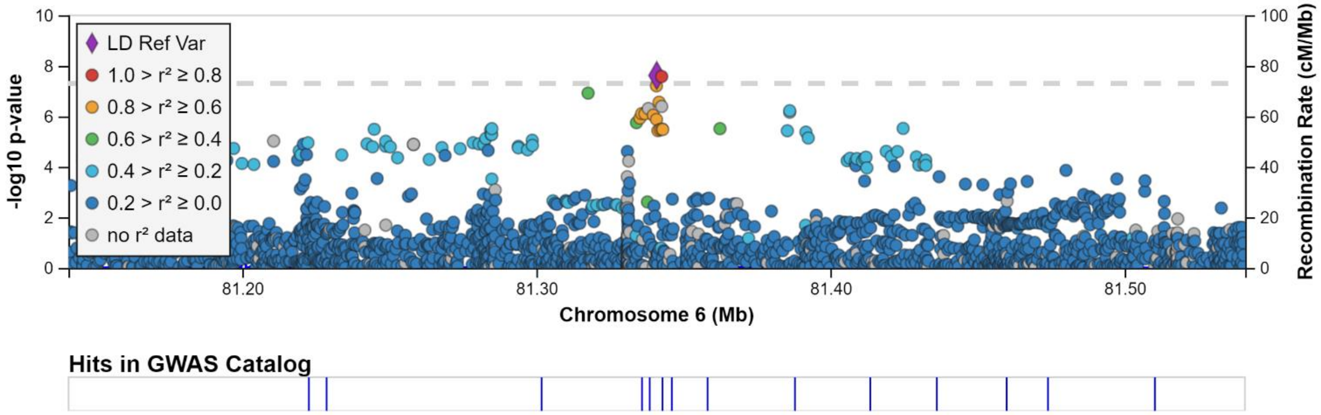

IL1-RA - Base Model - rs202017265

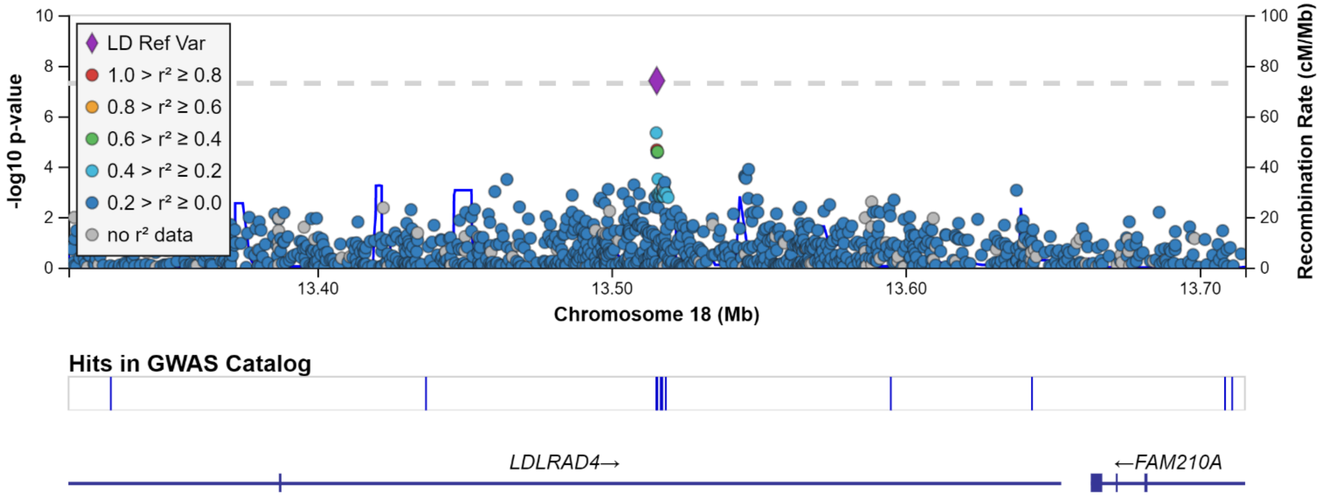

IL-6 - Base Model - rs72911283

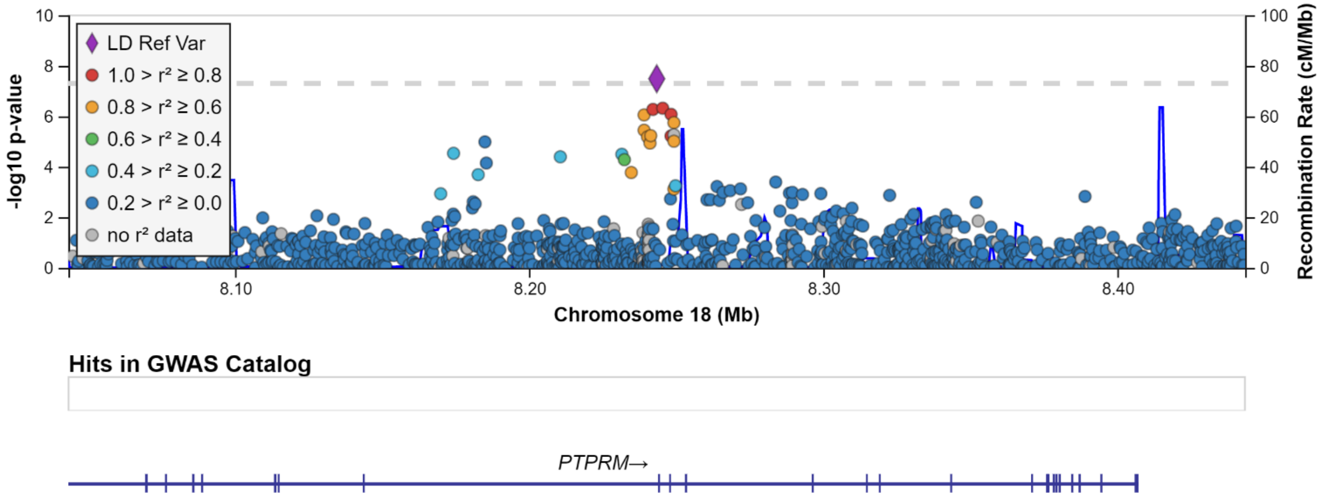

Glucagon - Base Model - rs10809430

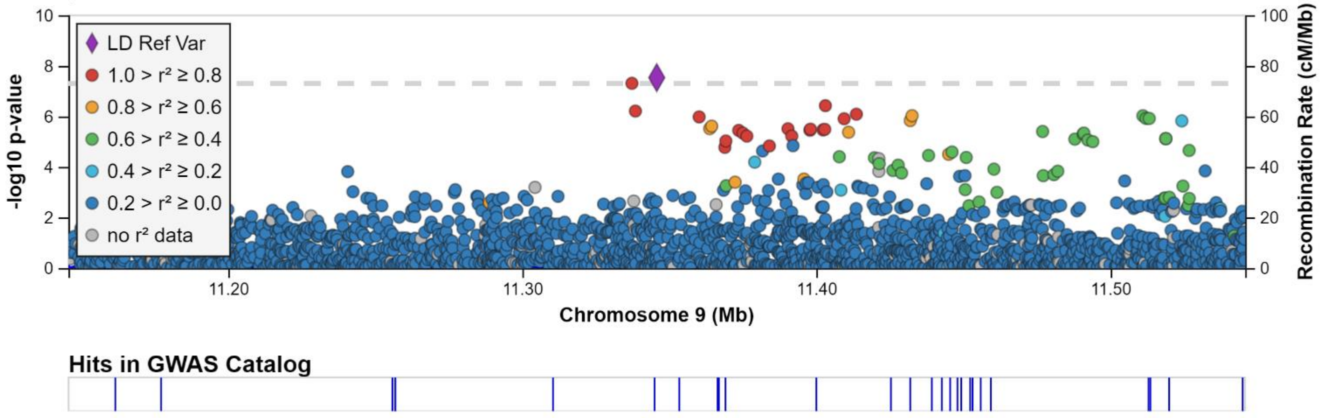

Ghrelin - Base Model - 3:49318960*

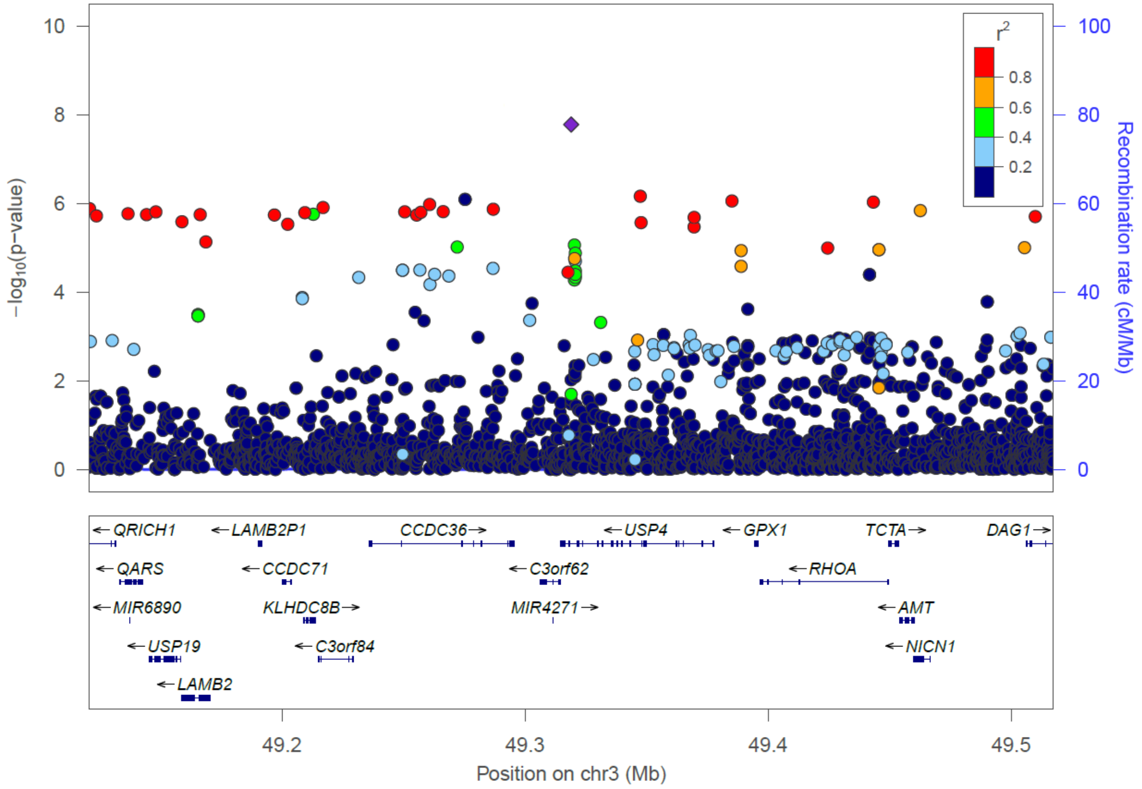

Ghrelin - Base Model - rs150921599

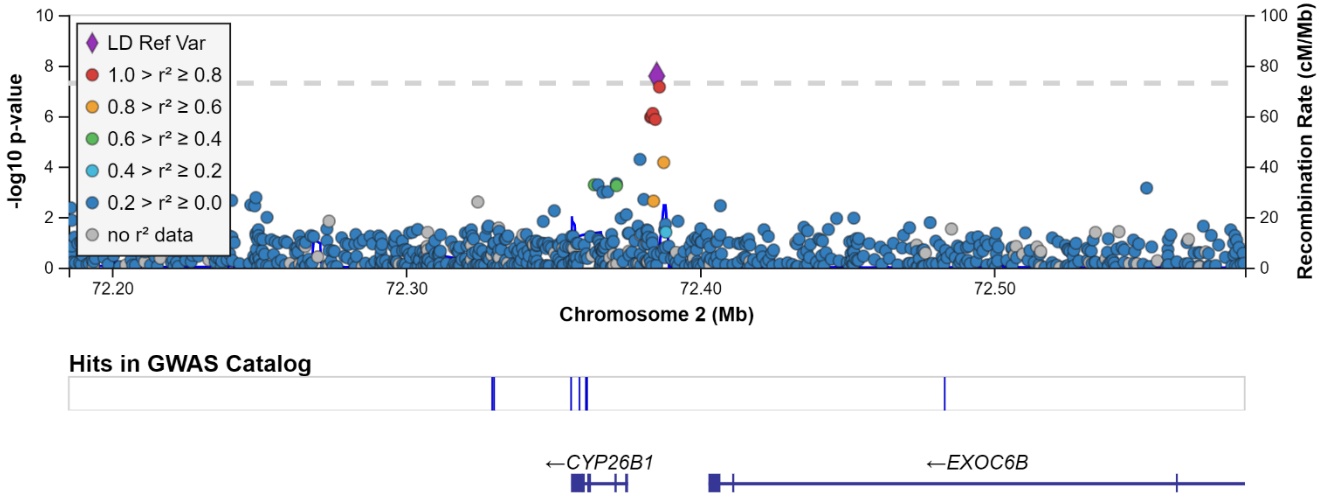

Ghrelin - Base Model - rs183921098

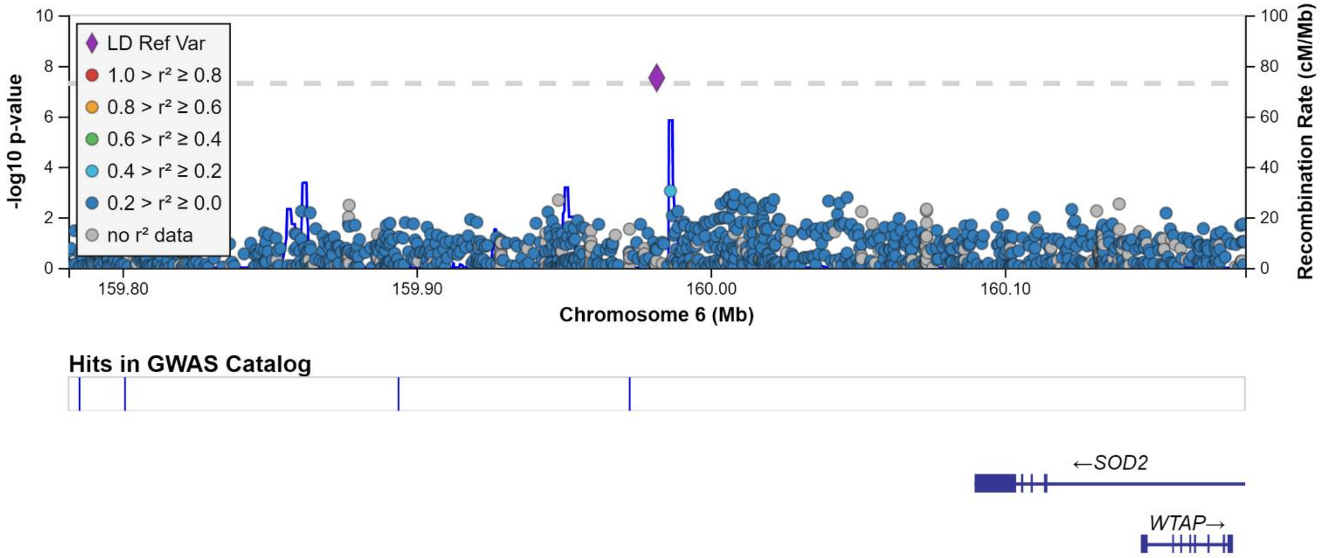

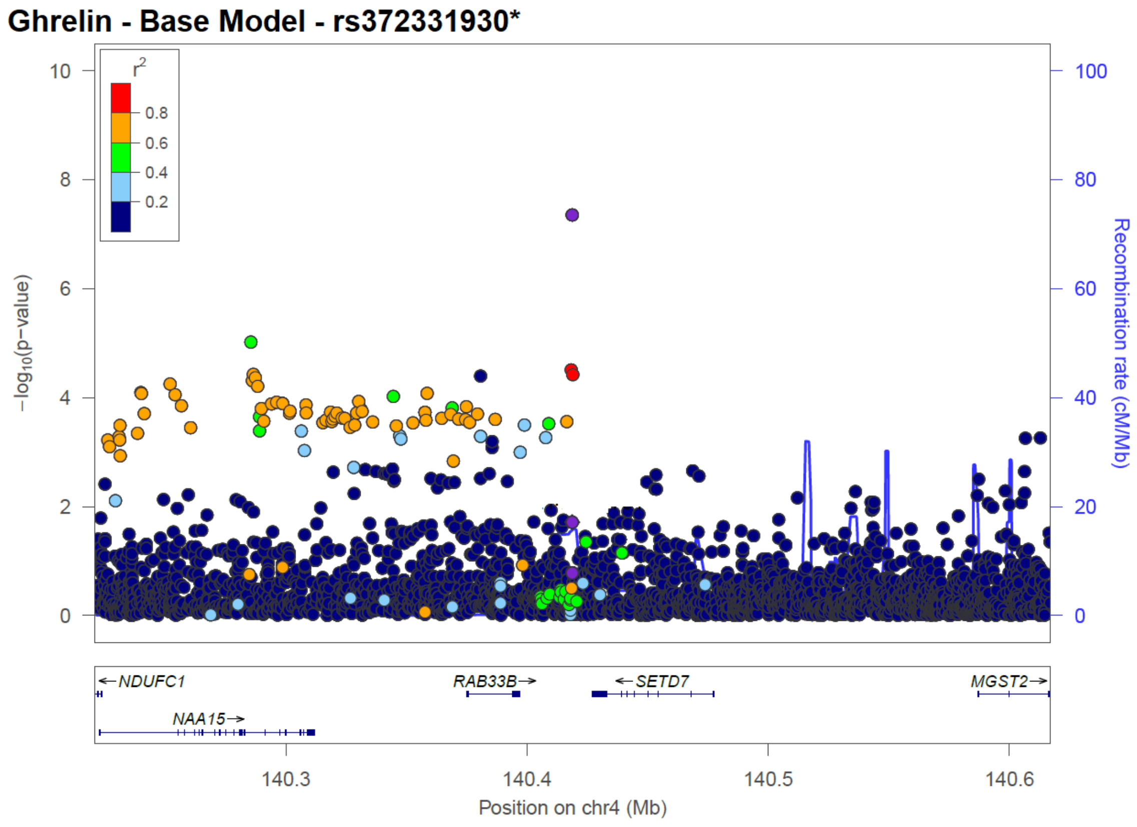

Ghrelin - Base Model - rs372331930*
r2
0.8
0.6
0.4
0.2
10
8
6
4
2
0
100
80
60
40
20
0
−log10(p−value)
Recombination rate (cM/Mb)
NDUFC1
NAA15
RAB33B
SETD7
MGST2
140.3
140.4
140.5
140.6
Position on chr4 (Mb)

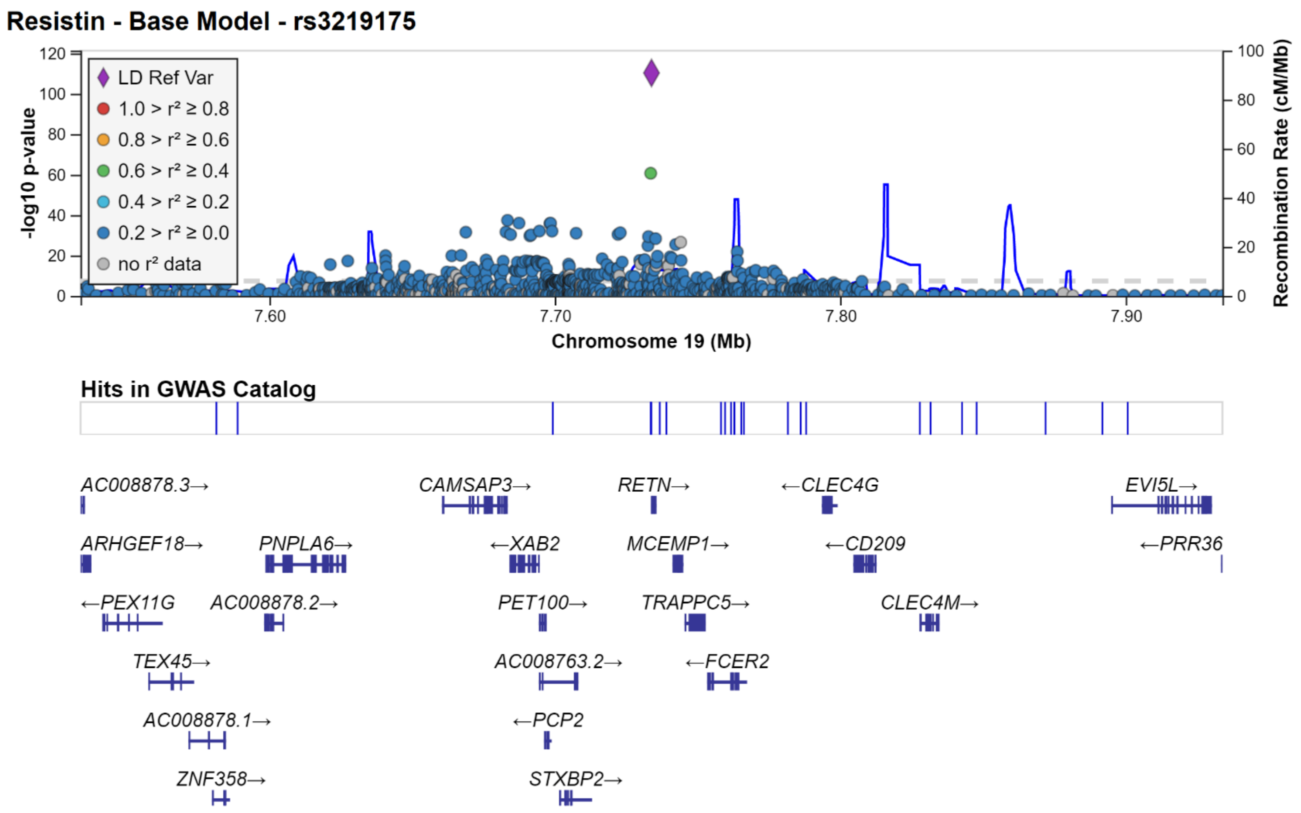

Resistin - Base Model - rs3219175
LD Ref Var
1.0 > r² ≥ 0.8
0.8 > r² ≥ 0.6
0.6 > r² ≥ 0.4
0.4 > r² ≥ 0.2
0.2 > r² ≥ 0.0
no r² data
-log10 p-value
Recombination Rate (cM/Mb)
Chromosome 19 (Mb)
7.60
7.70
7.80
7.90
Hits in GWAS Catalog
AC008878.3→
ARHGEF18→
←PEX11G
TEX45→
AC008878.1→
ZNF358→
PNPLA6→
AC008878.2→
CAMSAP3→
←XAB2
PET100→
AC008763.2→
←PCP2
STXBP2→
RETN→
MCEMP1→
TRAPPC5→
←FCER2
←CLEC4G
←CD209
CLEC4M→
EVI5L→
←PRR36

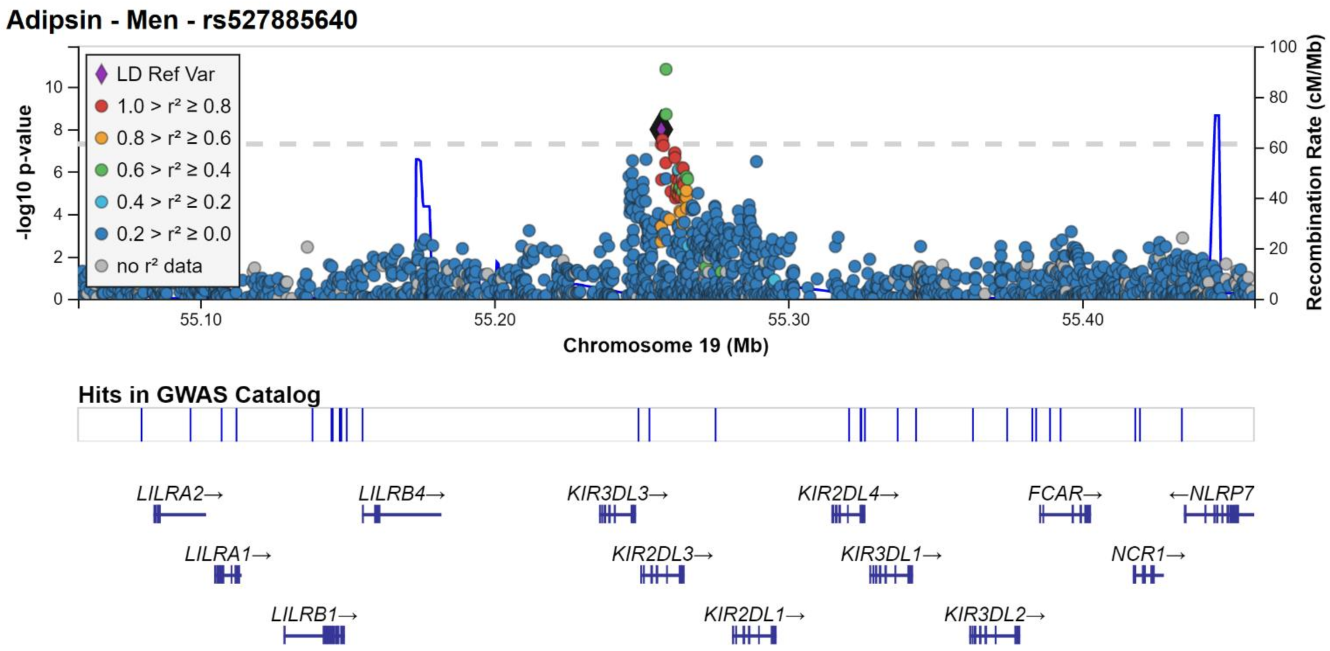
Adipsin - Men - rs527885640
LD Ref Var
1.0 > r² ≥ 0.8
0.8 > r² ≥ 0.6
0.6 > r² ≥ 0.4
0.4 > r² ≥ 0.2
0.2 > r² ≥ 0.0
no r² data
-log10 p-value
Recombination Rate (cM/Mb)
Chromosome 19 (Mb)
55.10
55.20
55.30
55.40
Hits in GWAS Catalog
LILRA2→
LILRA1→
LILRB1→
LILRB4→
KIR3DL3→
KIR2DL3→
KIR2DL1→
KIR2DL4→
KIR3DL1→
KIR3DL2→
FCAR→
NCR1→
←NLRP7

Adipsin - Men - rs61848529*

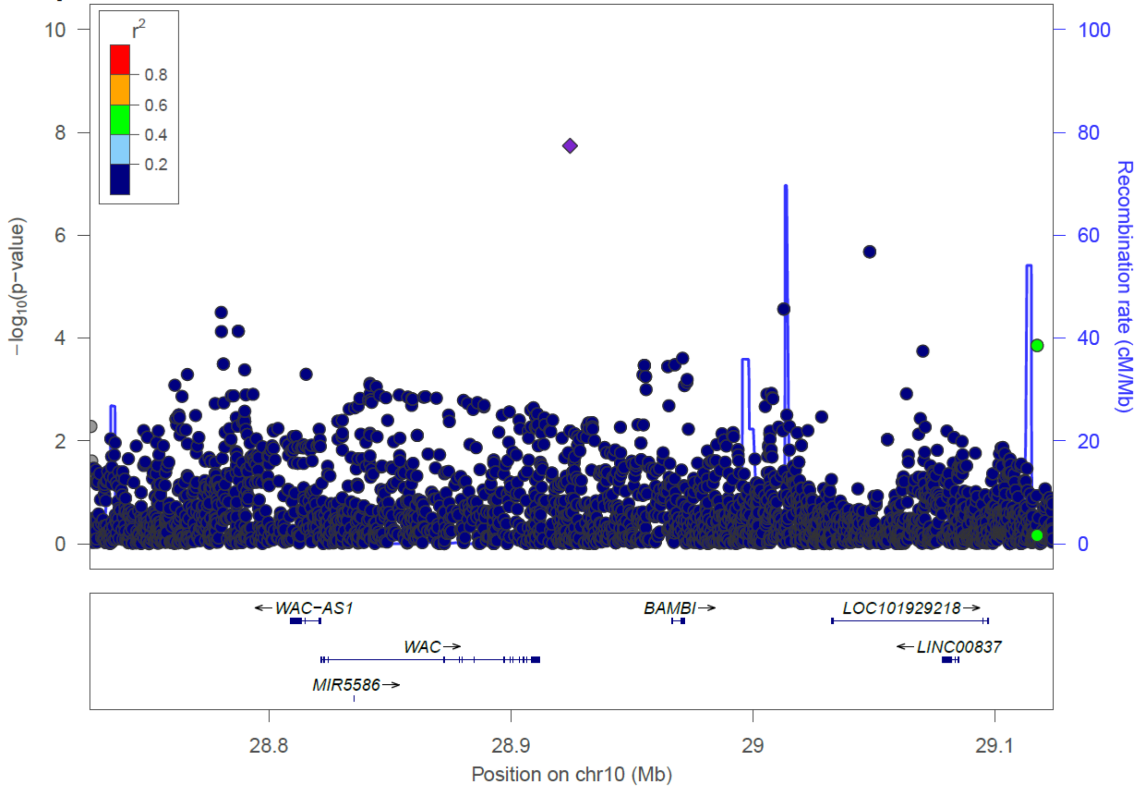

GLP-1 - Men - rs2974244

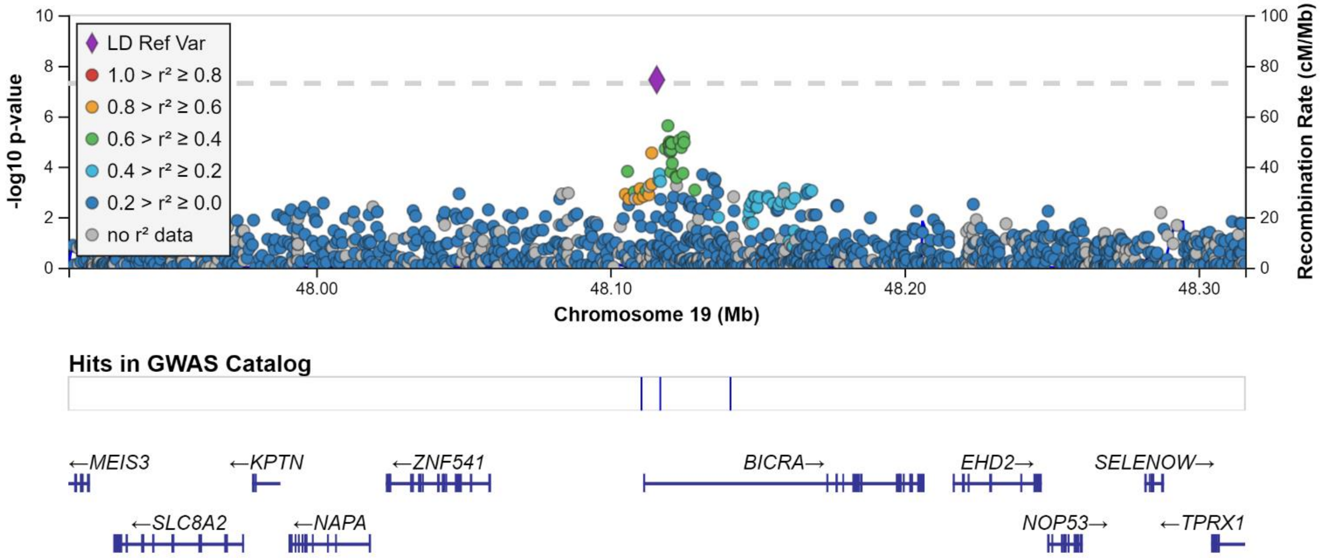

Insulin - Men - rs1248438

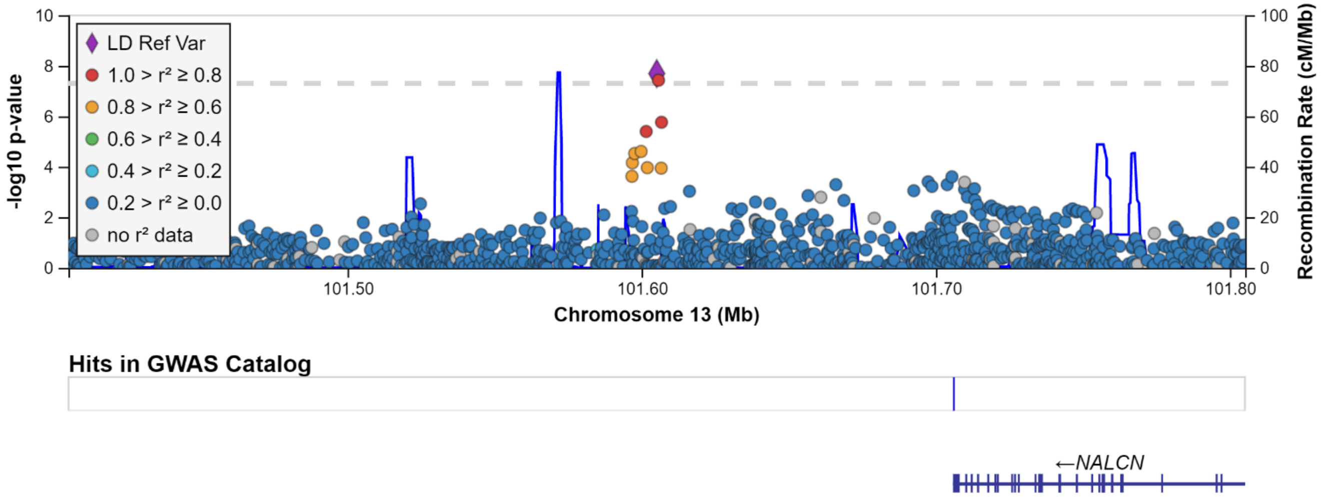

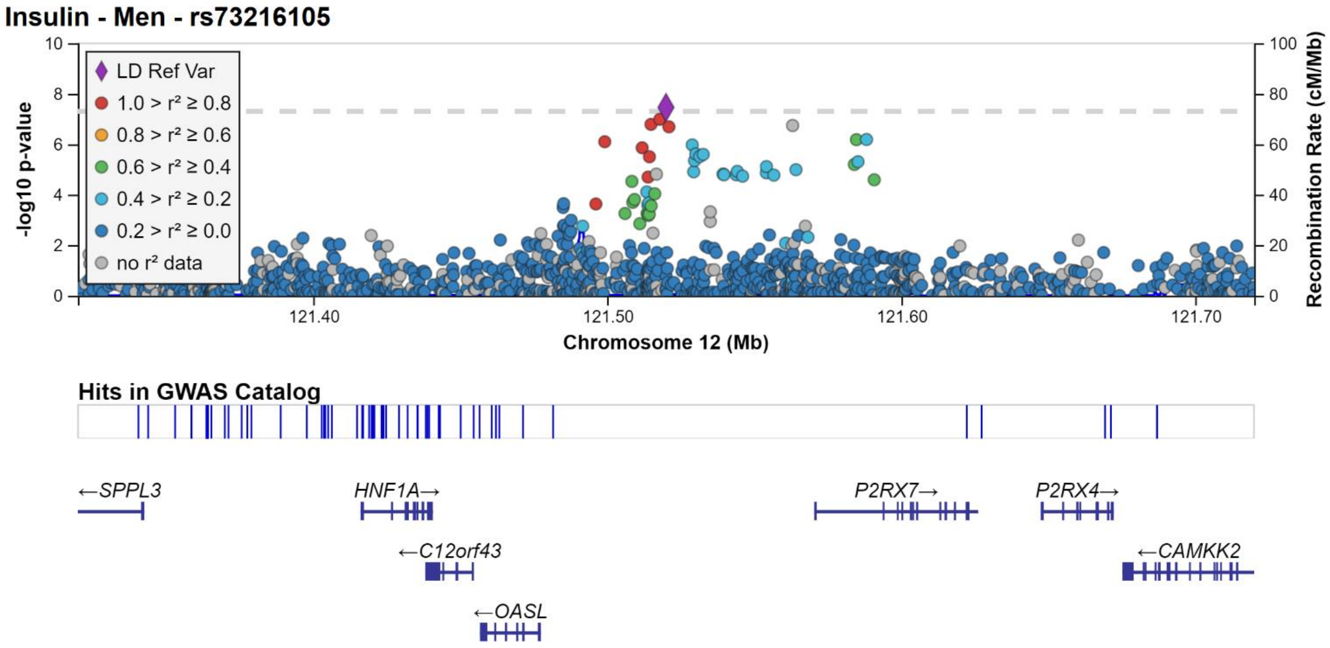
Insulin - Men - rs73216105
LD Ref Var
1.0 > r² ≥ 0.8
0.8 > r² ≥ 0.6
0.6 > r² ≥ 0.4
0.4 > r² ≥ 0.2
0.2 > r² ≥ 0.0
no r² data
-log10 p-value
Recombination Rate (cM/Mb)
Chromosome 12 (Mb)
121.40
121.50
121.60
121.70
Hits in GWAS Catalog
←SPPL3
HNF1A→
P2RX7→
P2RX4→
←C12orf43
←CAMKK2
←OASL

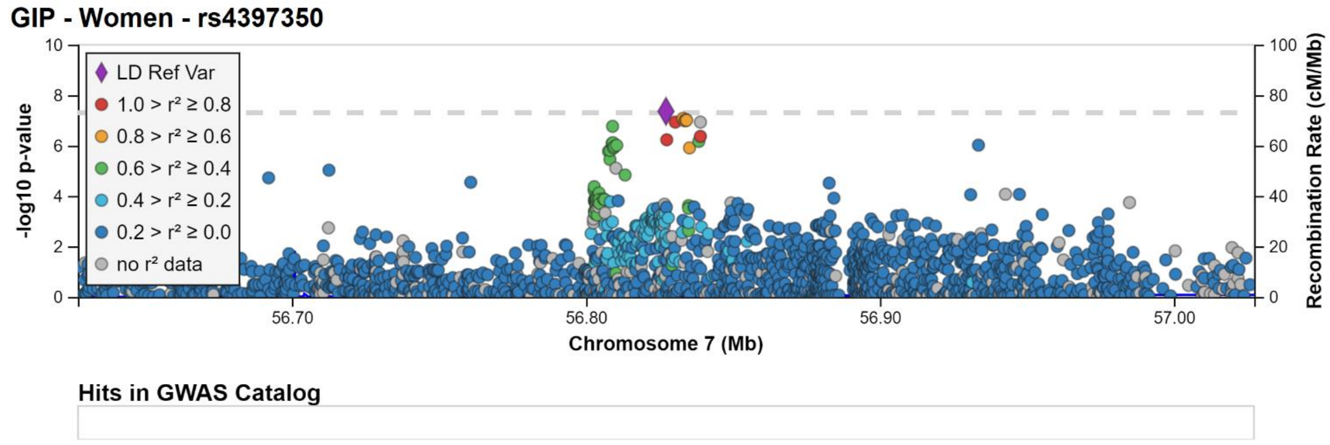
GIP - Women - rs4397350
LD Ref Var
1.0 > r² ≥ 0.8
0.8 > r² ≥ 0.6
0.6 > r² ≥ 0.4
0.4 > r² ≥ 0.2
0.2 > r² ≥ 0.0
no r² data
-log10 p-value
Recombination Rate (cM/Mb)
Chromosome 7 (Mb)
56.70
56.80
56.90
57.00
Hits in GWAS Catalog

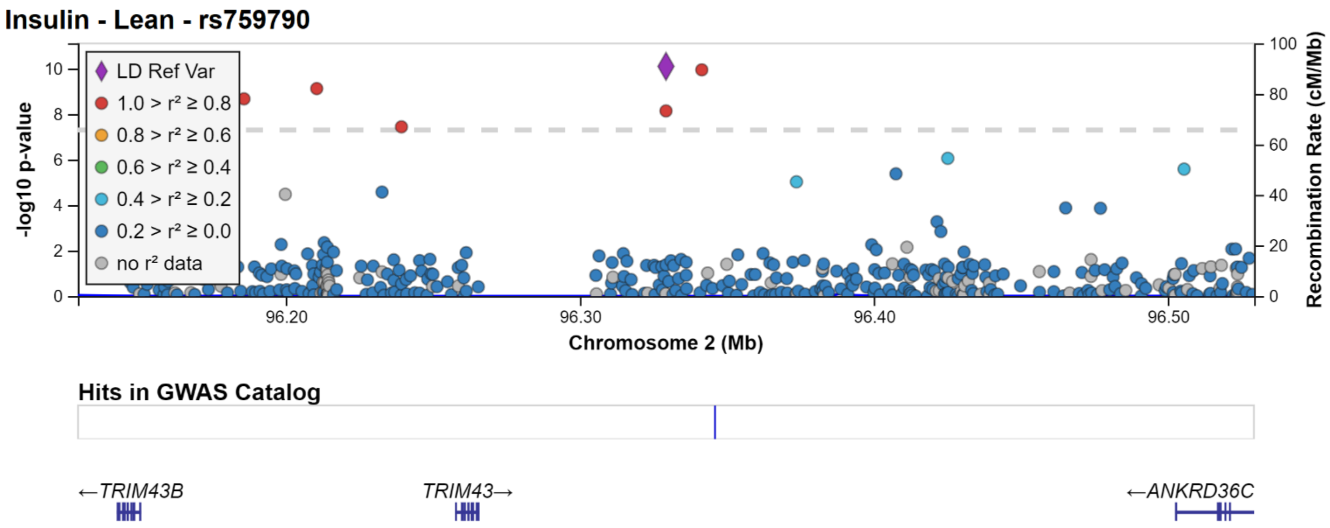
Insulin - Lean - rs759790
LD Ref Var
1.0 > r² ≥ 0.8
0.8 > r² ≥ 0.6
0.6 > r² ≥ 0.4
0.4 > r² ≥ 0.2
0.2 > r² ≥ 0.0
no r² data
-log10 p-value
Recombination Rate (cM/Mb)
96.20
96.30
96.40
96.50
Chromosome 2 (Mb)
Hits in GWAS Catalog
←TRIM43B
TRIM43→
←ANKRD36C

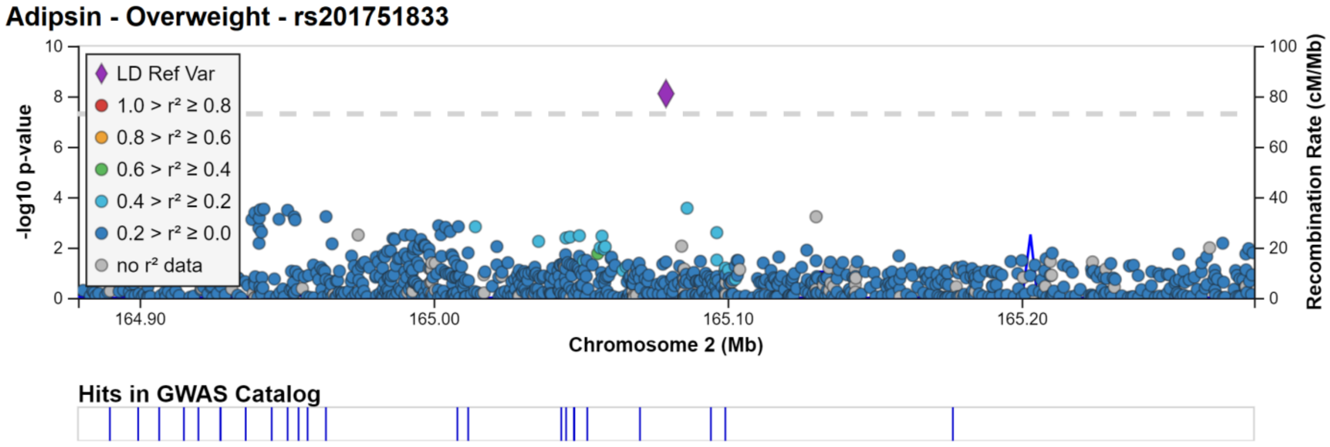
Adipsin - Overweight - rs201751833
LD Ref Var
1.0 > r² ≥ 0.8
0.8 > r² ≥ 0.6
0.6 > r² ≥ 0.4
0.4 > r² ≥ 0.2
0.2 > r² ≥ 0.0
no r² data
-log10 p-value
Recombination Rate (cM/Mb)
164.90
165.00
165.10
165.20
Chromosome 2 (Mb)
Hits in GWAS Catalog

Fig S3: Manhattan plots for meta-analysis of discovery and replication cohorts combined for all 13 obesity- and diabetes-related cytokines and hormones.

IG = Intergenic. Loci in red had a P-value of <5×10^{-8} and loci in orange a P-value of <5×10^{-7}.

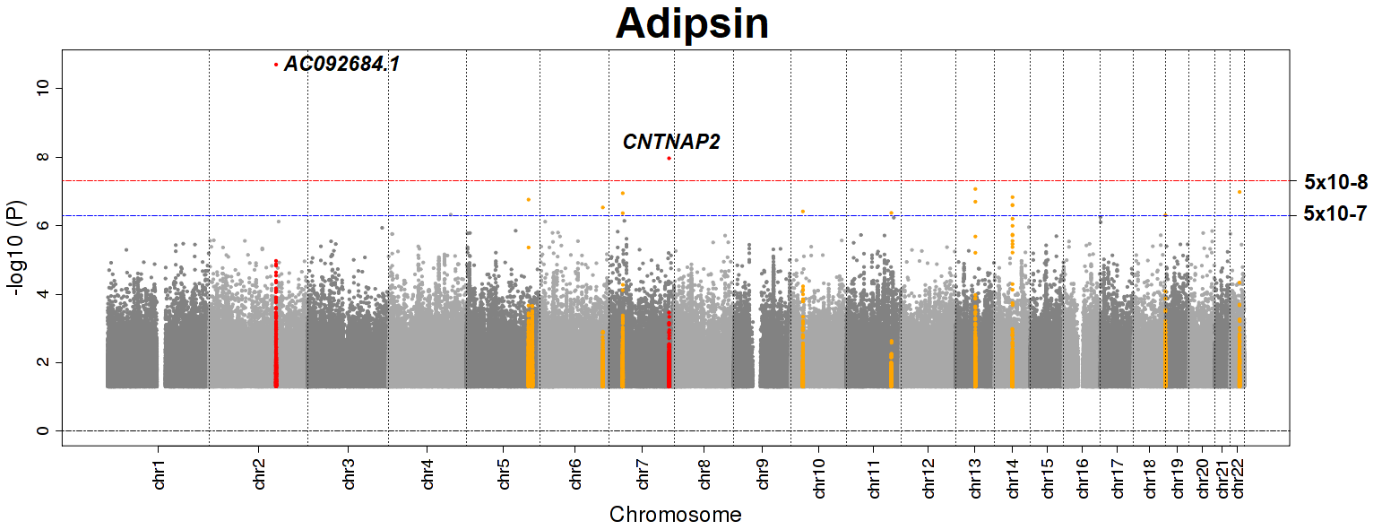

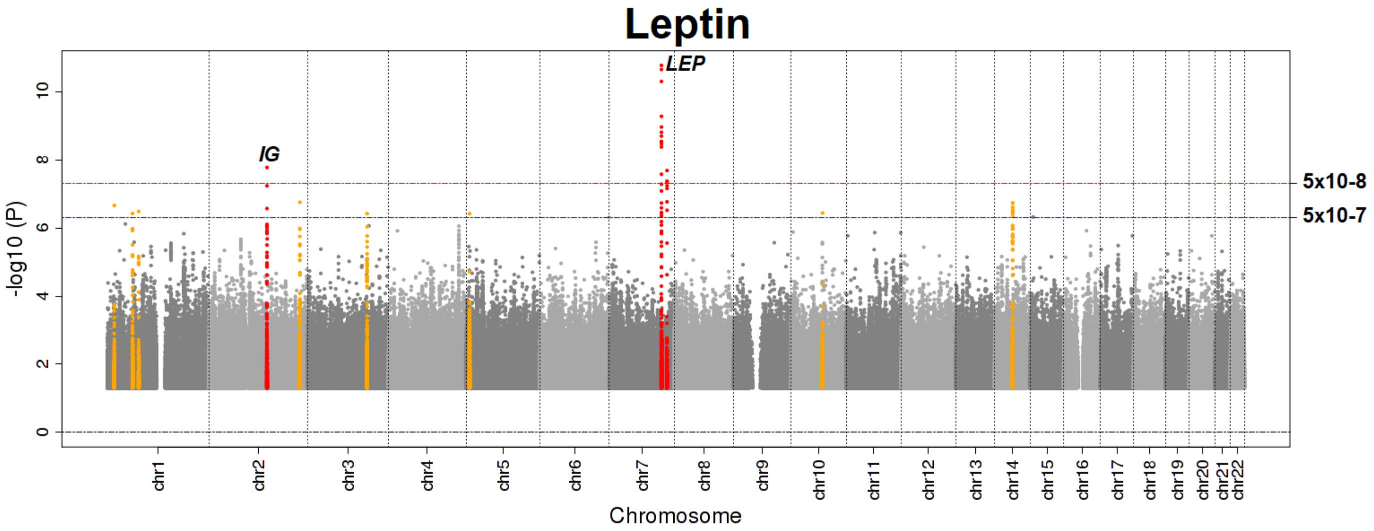

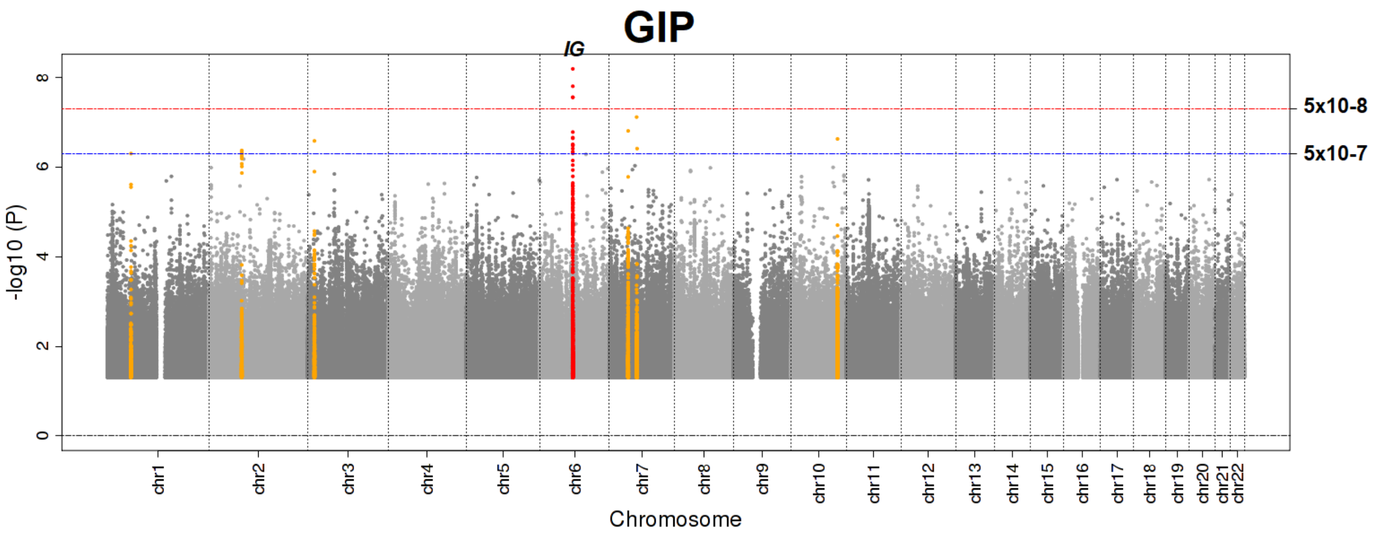
GIP
IG
5x10-8
5x10-7
-log10 (P)
0
2
4
6
8
chr1
chr2
chr3
chr4
chr5
chr6
chr7
chr8
chr9
chr10
chr11
chr12
chr13
chr14
chr15
chr16
chr17
chr18
chr19
chr20
chr21
chr22
Chromosome

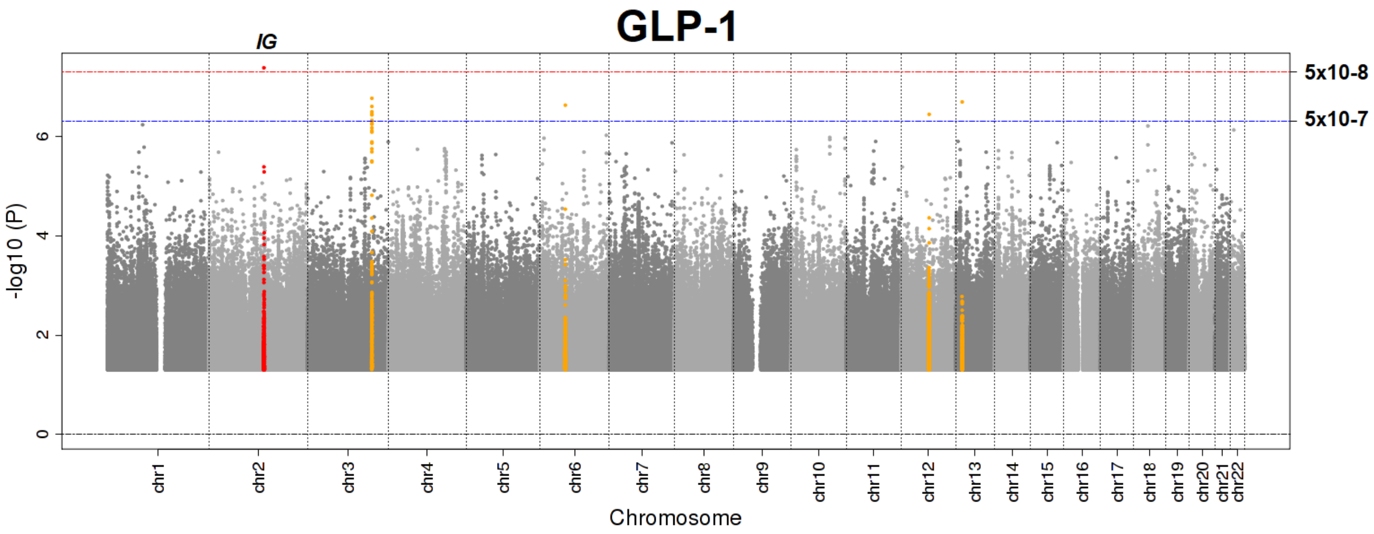
GLP-1
IG
5x10-8
5x10-7
-log10 (P)
0
2
4
6
chr1
chr2
chr3
chr4
chr5
chr6
chr7
chr8
chr9
chr10
chr11
chr12
chr13
chr14
chr15
chr16
chr17
chr18
chr19
chr20
chr21
chr22
Chromosome

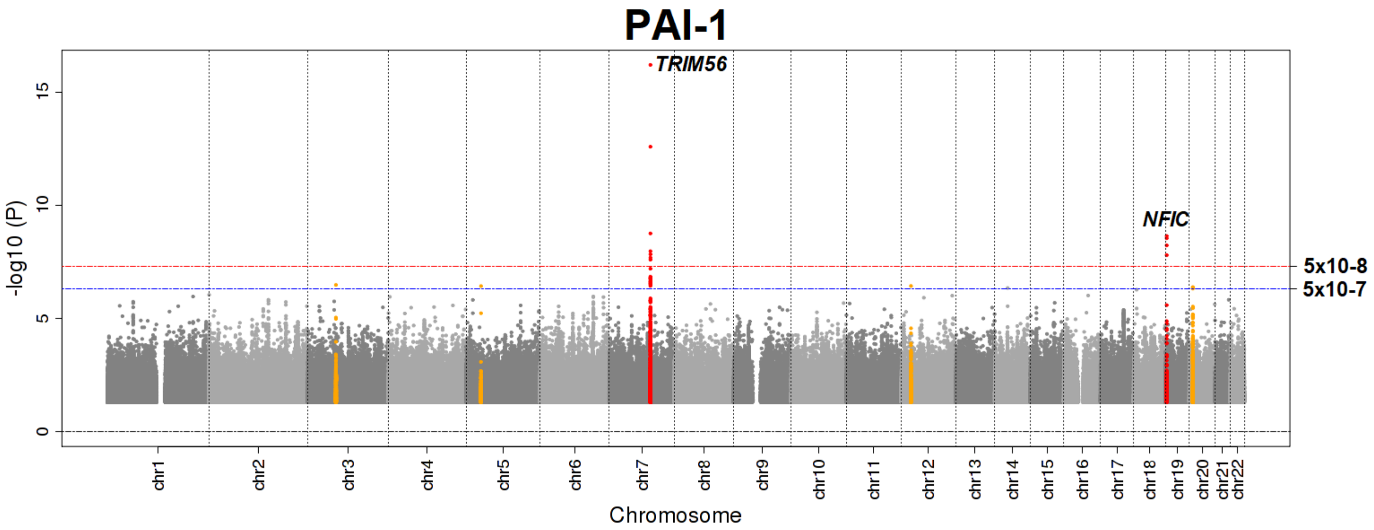
PAI-1
TRIM56
NFIC
5x10-8
5x10-7
-log10 (P)
Chromosome

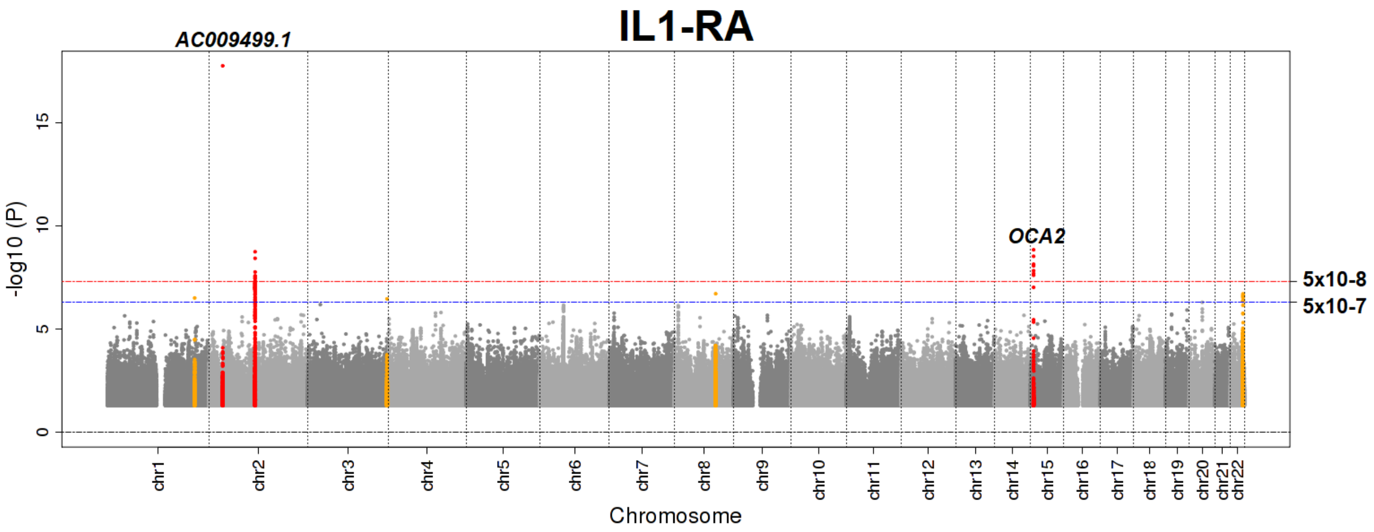
IL1-RA
AC009499.1
OCA2
5x10-8
5x10-7
-log10 (P)
Chromosome

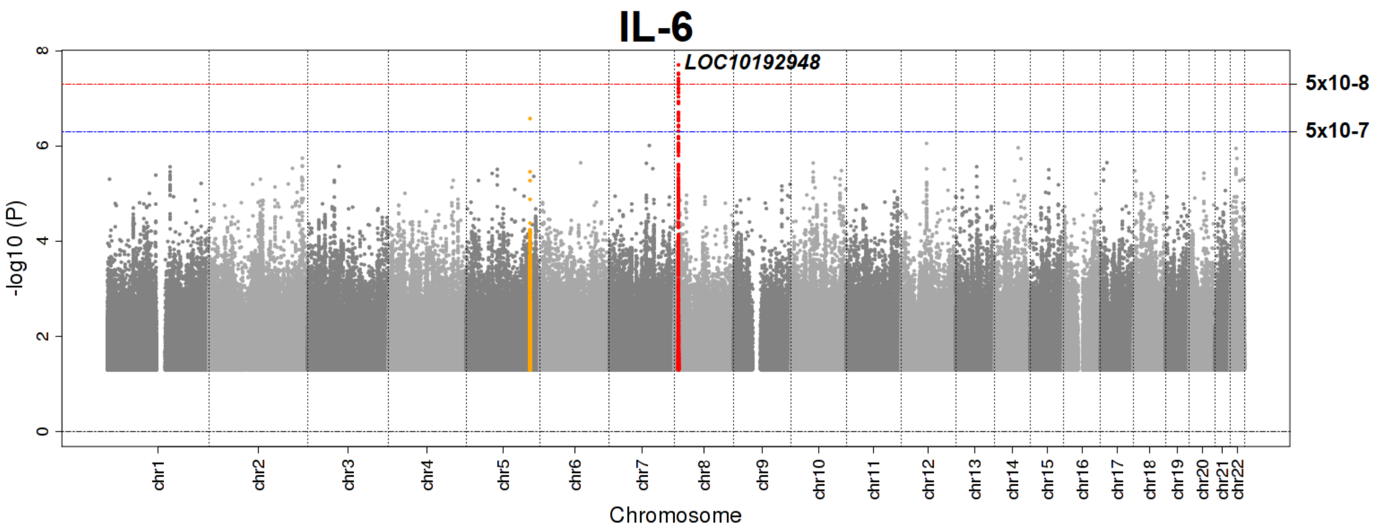
IL-6
LOC10192948
5x10-8
5x10-7
-log10 (P)
0
2
4
6
8
chr1 chr2 chr3 chr4 chr5 chr6 chr7 chr8 chr9 chr10 chr11 chr12 chr13 chr14 chr15 chr16 chr17 chr18 chr19 chr20 chr21 chr22
Chromosome

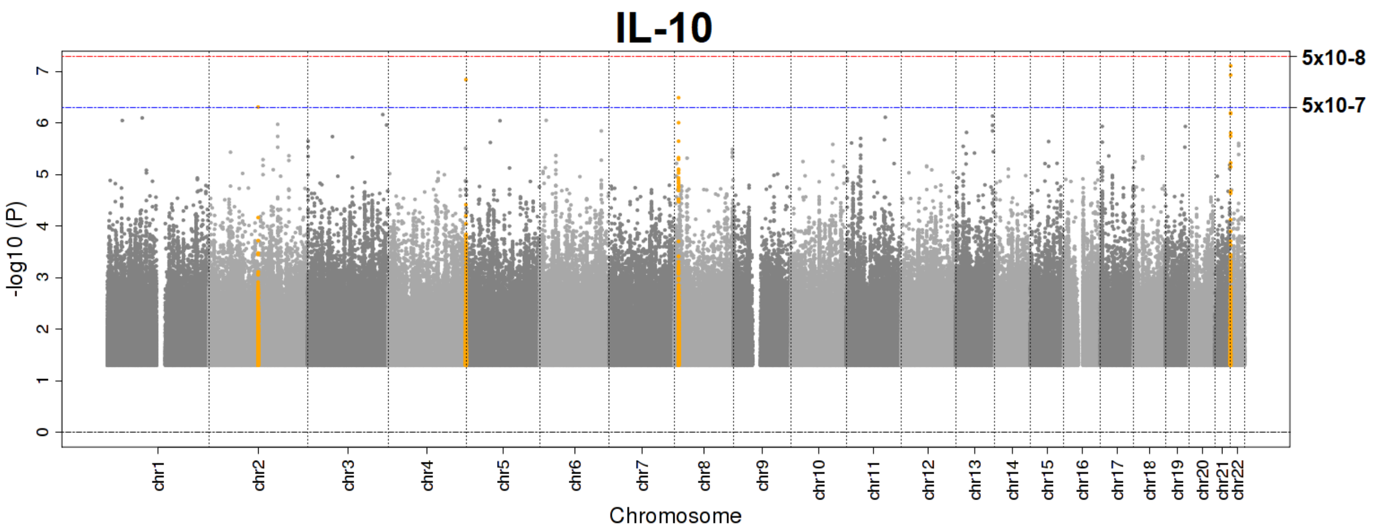
IL-10
5x10-8
5x10-7
-log10 (P)
0
1
2
3
4
5
6
7
chr1 chr2 chr3 chr4 chr5 chr6 chr7 chr8 chr9 chr10 chr11 chr12 chr13 chr14 chr15 chr16 chr17 chr18 chr19 chr20 chr21 chr22
Chromosome

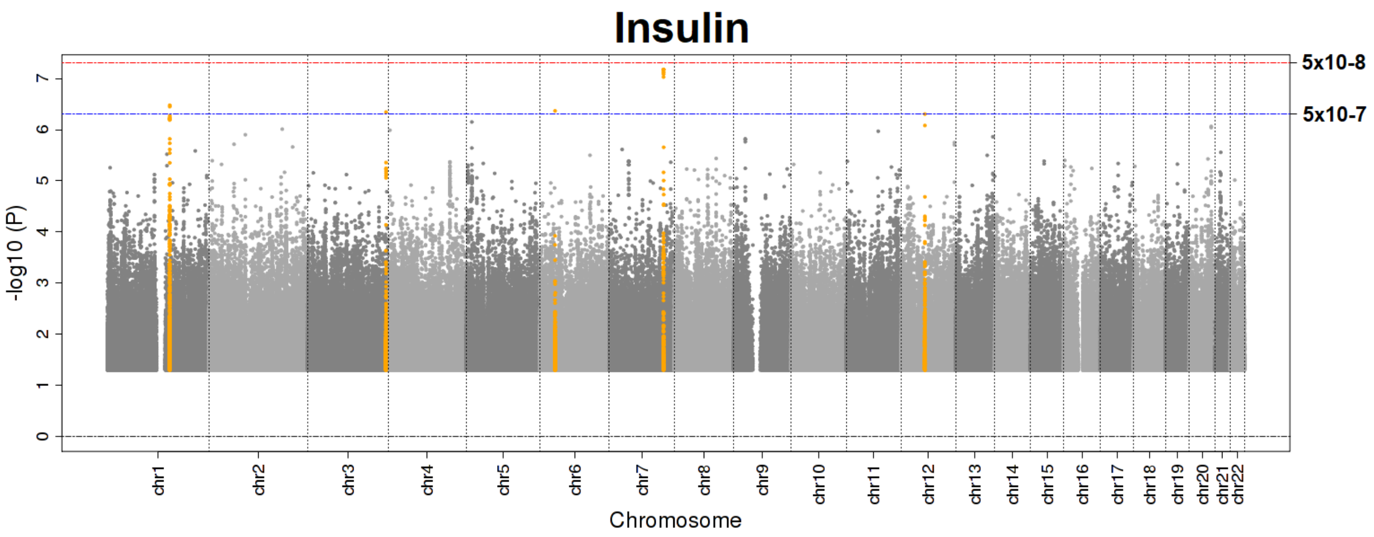

Insulin
5x10-8
5x10-7
-log10 (P)
chr1
chr2
chr3
chr4
chr5
chr6
chr7
chr8
chr9
chr10
chr11
chr12
chr13
chr14
chr15
chr16
chr17
chr18
chr19
chr20
chr21
chr22
Chromosome

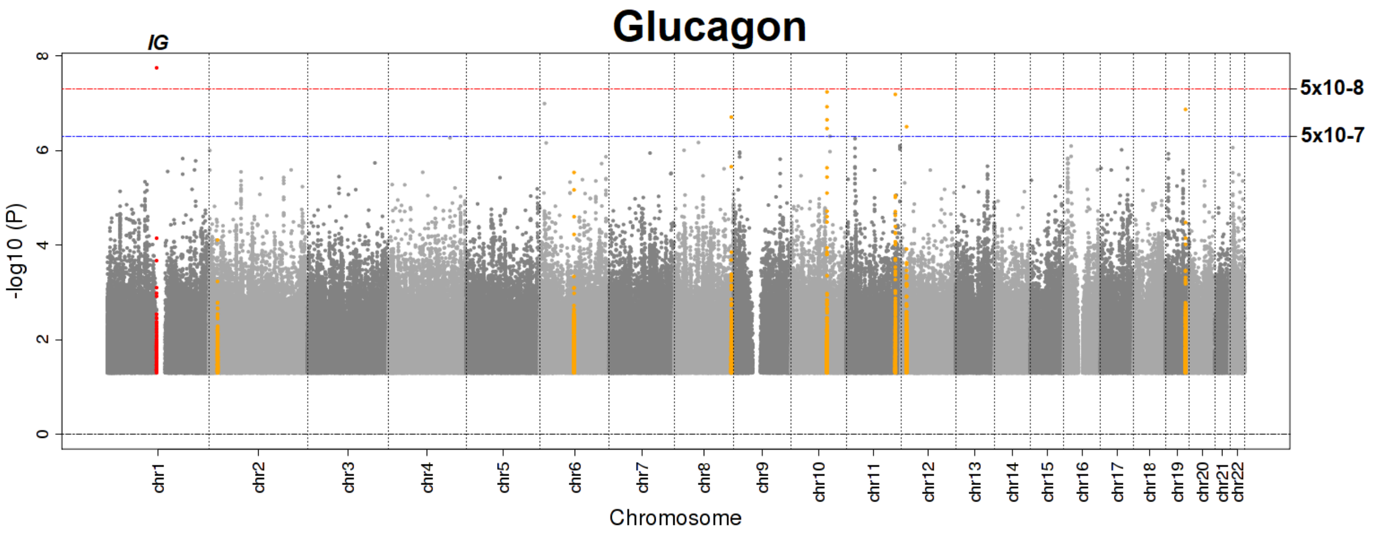

Glucagon
IG
5x10-8
5x10-7
-log10 (P)
chr1
chr2
chr3
chr4
chr5
chr6
chr7
chr8
chr9
chr10
chr11
chr12
chr13
chr14
chr15
chr16
chr17
chr18
chr19
chr20
chr21
chr22
Chromosome

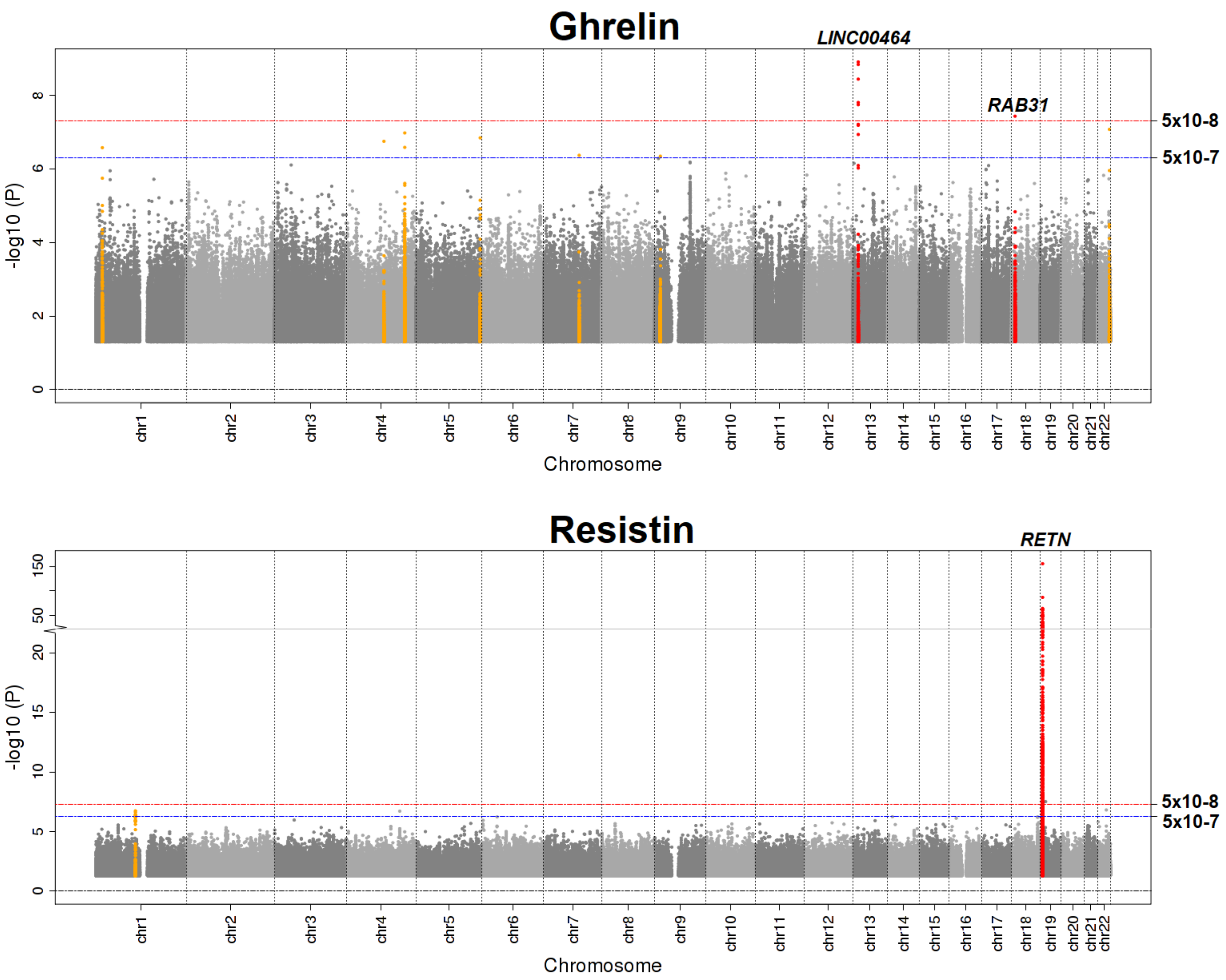
Ghrelin
LINC00464
RAB31
5x10-8
5x10-7
-log10 (P)
0
2
4
6
8
chr1
chr2
chr3
chr4
chr5
chr6
chr7
chr8
chr9
chr10
chr11
chr12
chr13
chr14
chr15
chr16
chr17
chr18
chr19
chr20
chr21
chr22
Chromosome
Resistin
RETN
5x10-8
5x10-7
-log10 (P)
0
5
10
15
20
50
150
chr1
chr2
chr3
chr4
chr5
chr6
chr7
chr8
chr9
chr10
chr11
chr12
chr13
chr14
chr15
chr16
chr17
chr18
chr19
chr20
chr21
chr22
Chromosome

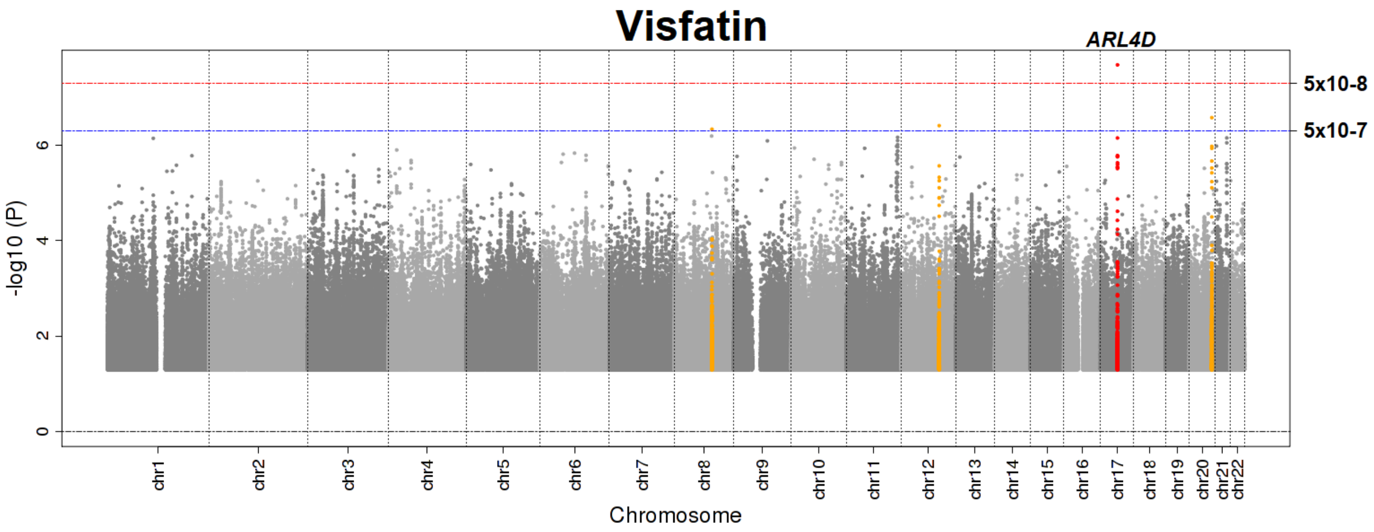

Visfatin
ARL4D
5x10-8
5x10-7
-log10 (P)
0
2
4
6
chr1
chr2
chr3
chr4
chr5
chr6
chr7
chr8
chr9
chr10
chr11
chr12
chr13
chr14
chr15
chr16
chr17
chr18
chr19
chr20
chr21
chr22
Chromosome

Fig S4: Miami plots for sex-stratified analyses for all 13 obesity- and diabetes-related cytokines and hormones.

IG = Intergenic. Loci in blue had a P-value of <5×10^{-8} in men and loci in pink had a P-value of <5×10^{-8} in women.

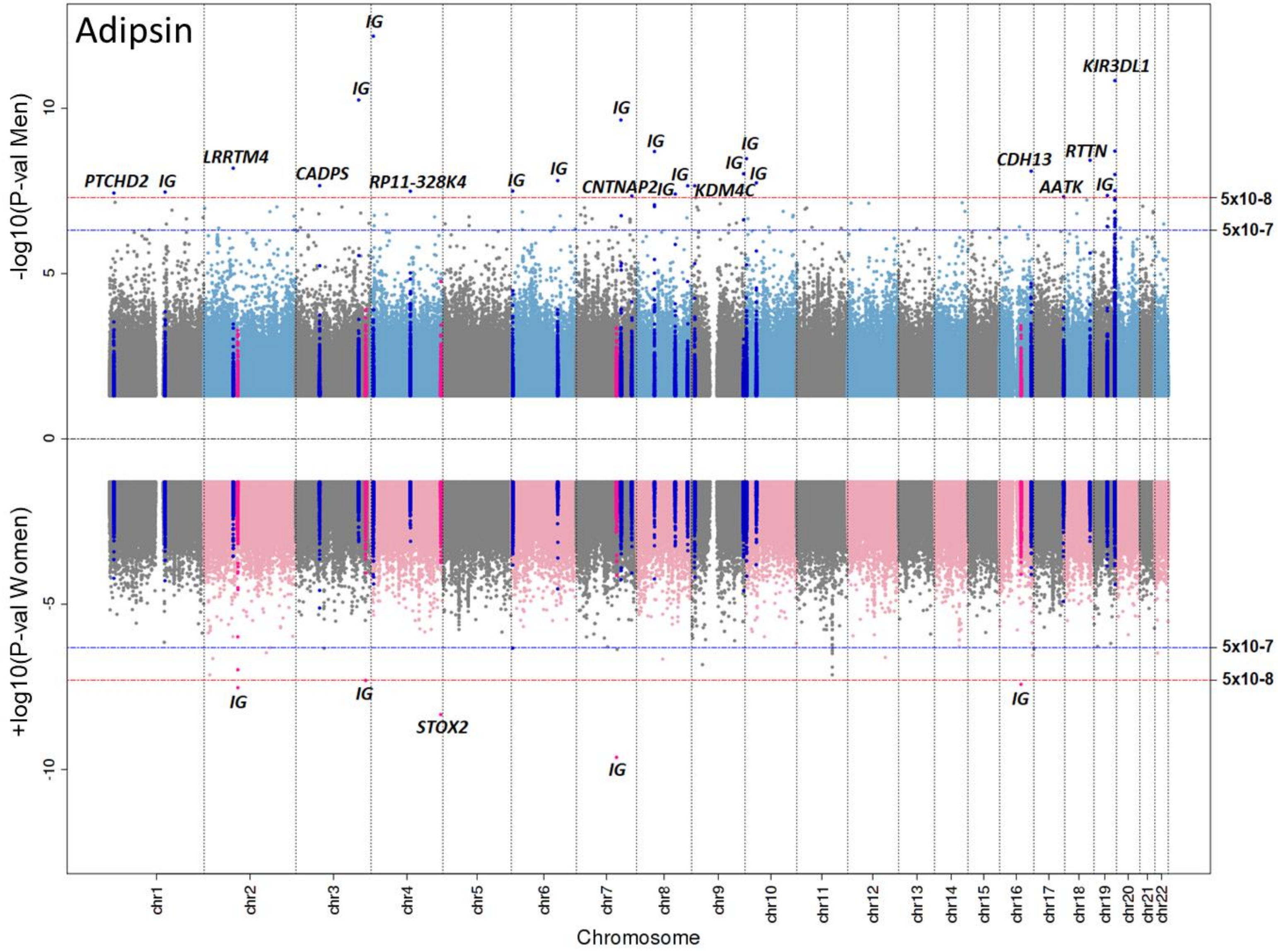

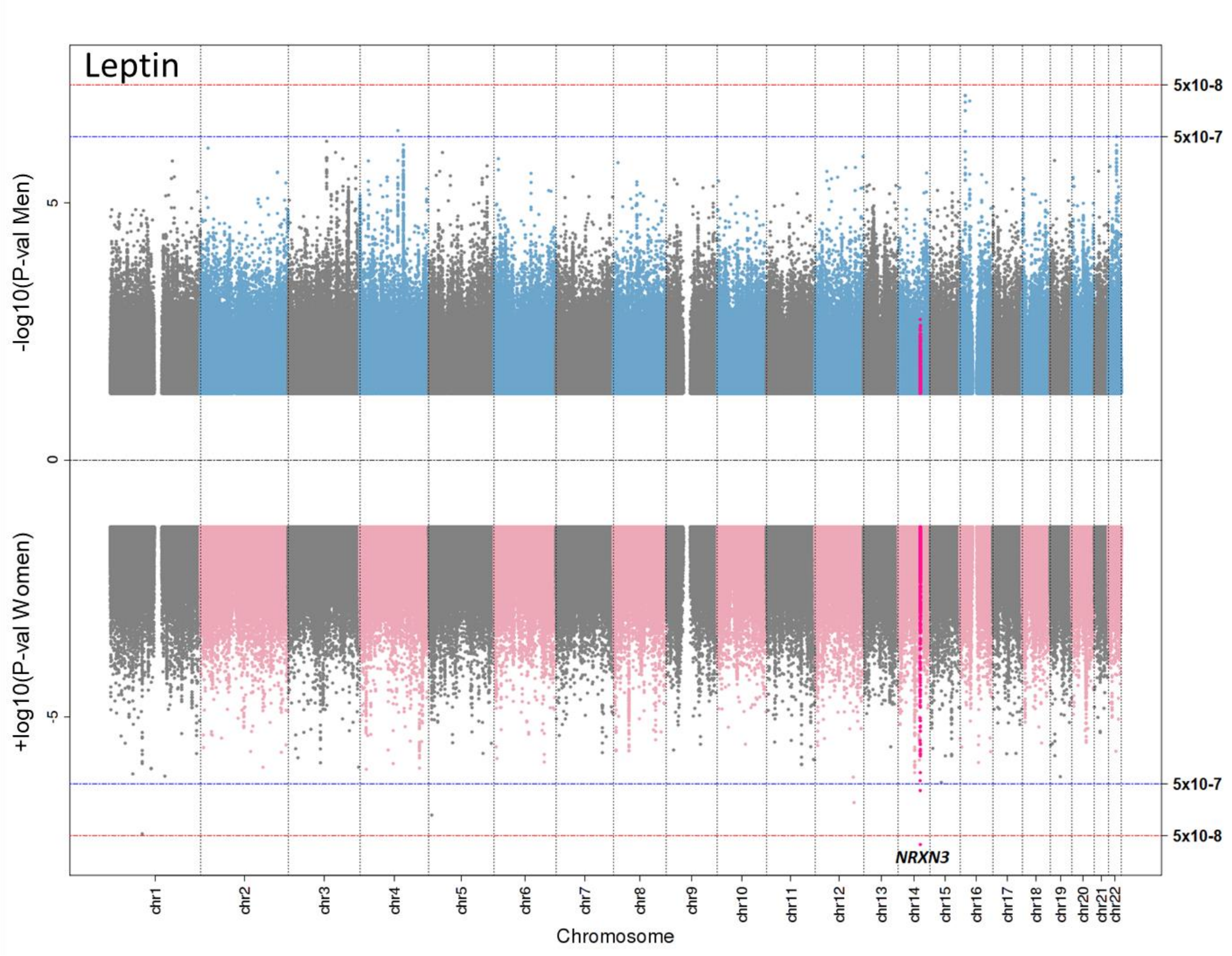

Leptin
5x10-8
5x10-7
5x10-7
5x10-8
-log10(P-val Men)
+log10(P-val Women)
5
0
-5
NRXN3
chr1
chr2
chr3
chr4
chr5
chr6
chr7
chr8
chr9
chr10
chr11
chr12
chr13
chr14
chr15
chr16
chr17
chr18
chr19
chr20
chr21
chr22
Chromosome

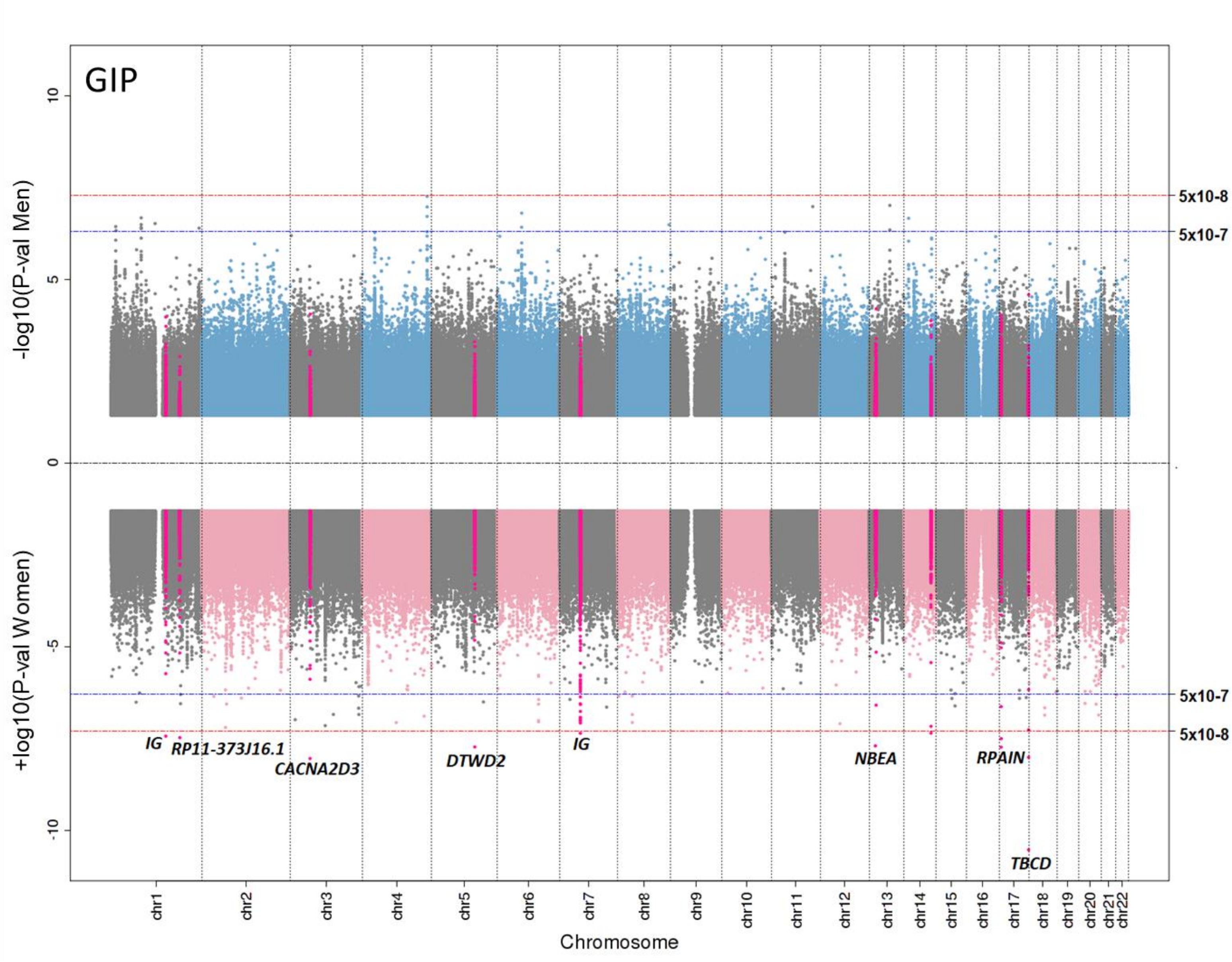
GIP
-log10(P-val Men)
+log10(P-val Women)
10
5
0
-5
-10
5x10-8
5x10-7
5x10-7
5x10-8
IG
RP11-373J16.1
CACNA2D3
DTWD2
IG
NBEA
RPAIN
TBCD
chr1
chr2
chr3
chr4
chr5
chr6
chr7
chr8
chr9
chr10
chr11
chr12
chr13
chr14
chr15
chr16
chr17
chr18
chr19
chr20
chr21
chr22
Chromosome

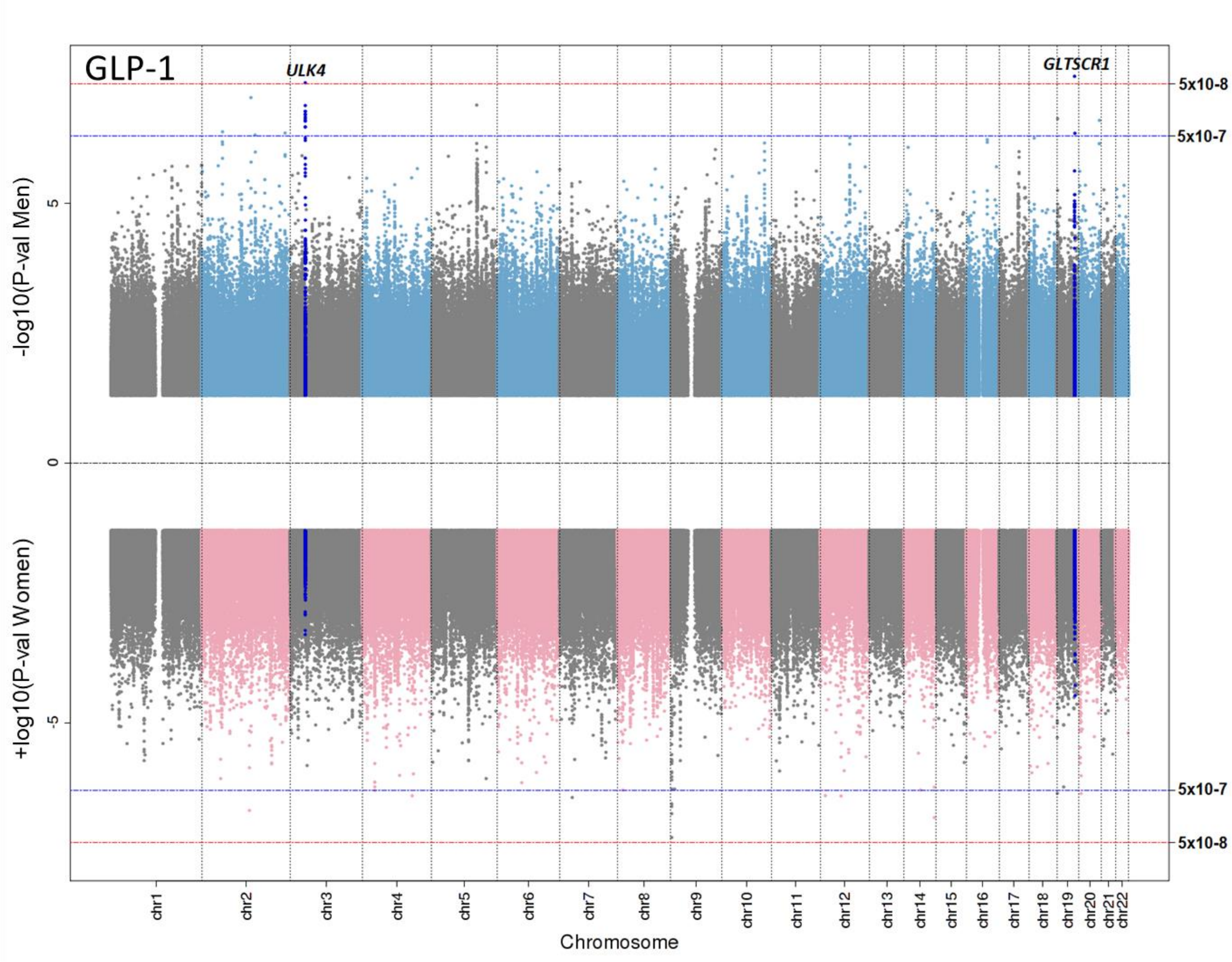
GLP-1
ULK4
GLTSCR1
5x10-8
5x10-7
5x10-7
5x10-8
-log10(P-val Men)
+log10(P-val Women)
5
0
-5
chr1
chr2
chr3
chr4
chr5
chr6
chr7
chr8
chr9
chr10
chr11
chr12
chr13
chr14
chr15
chr16
chr17
chr18
chr19
chr20
chr21
chr22
Chromosome

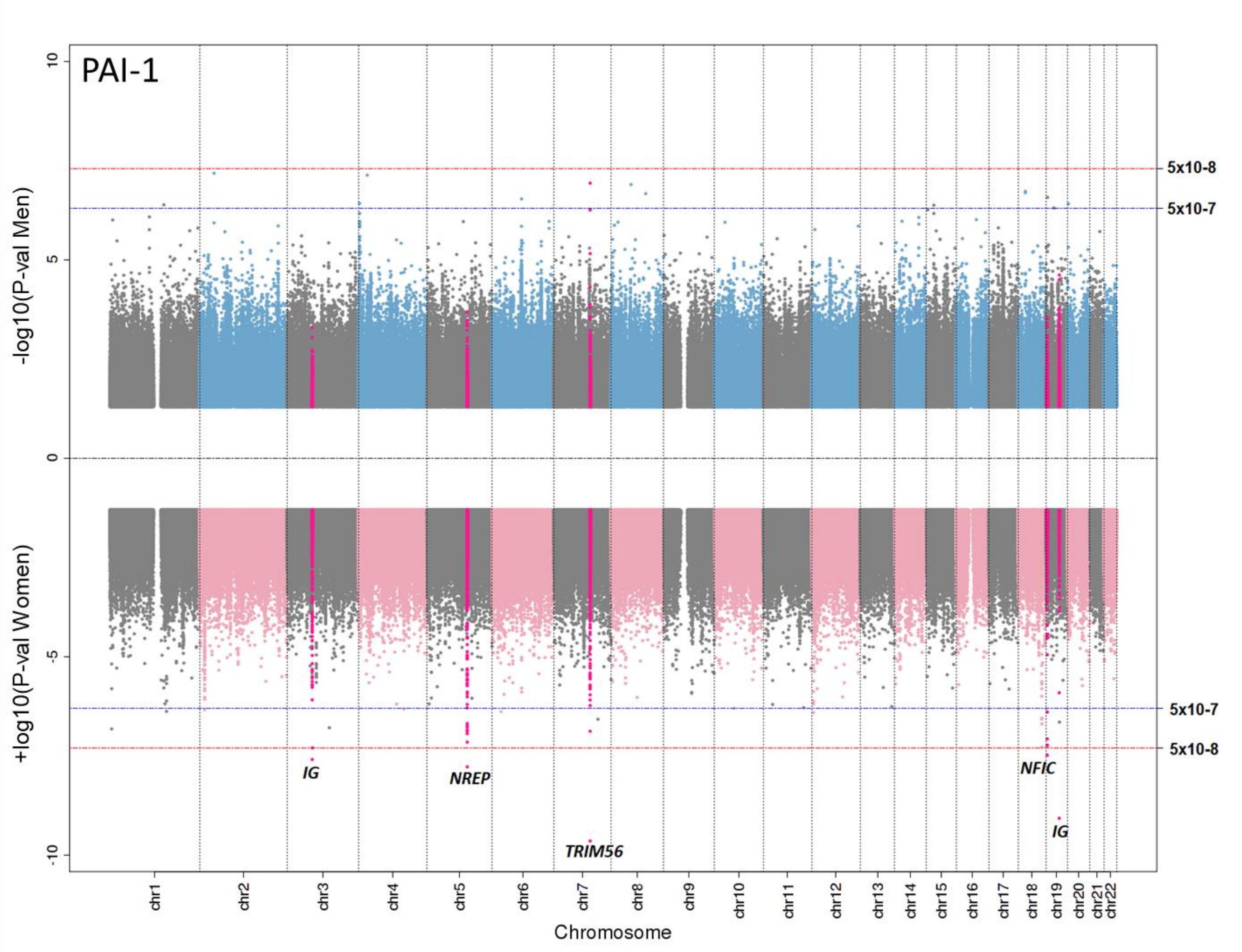
PAI-1
-log10(P-val Men)
+log10(P-val Women)
10
5
0
-5
-10
5x10-8
5x10-7
5x10-7
5x10-8
IG
NREP
TRIM56
NFIC
IG
chr1
chr2
chr3
chr4
chr5
chr6
chr7
chr8
chr9
chr10
chr11
chr12
chr13
chr14
chr15
chr16
chr17
chr18
chr19
chr20
chr21
chr22
Chromosome

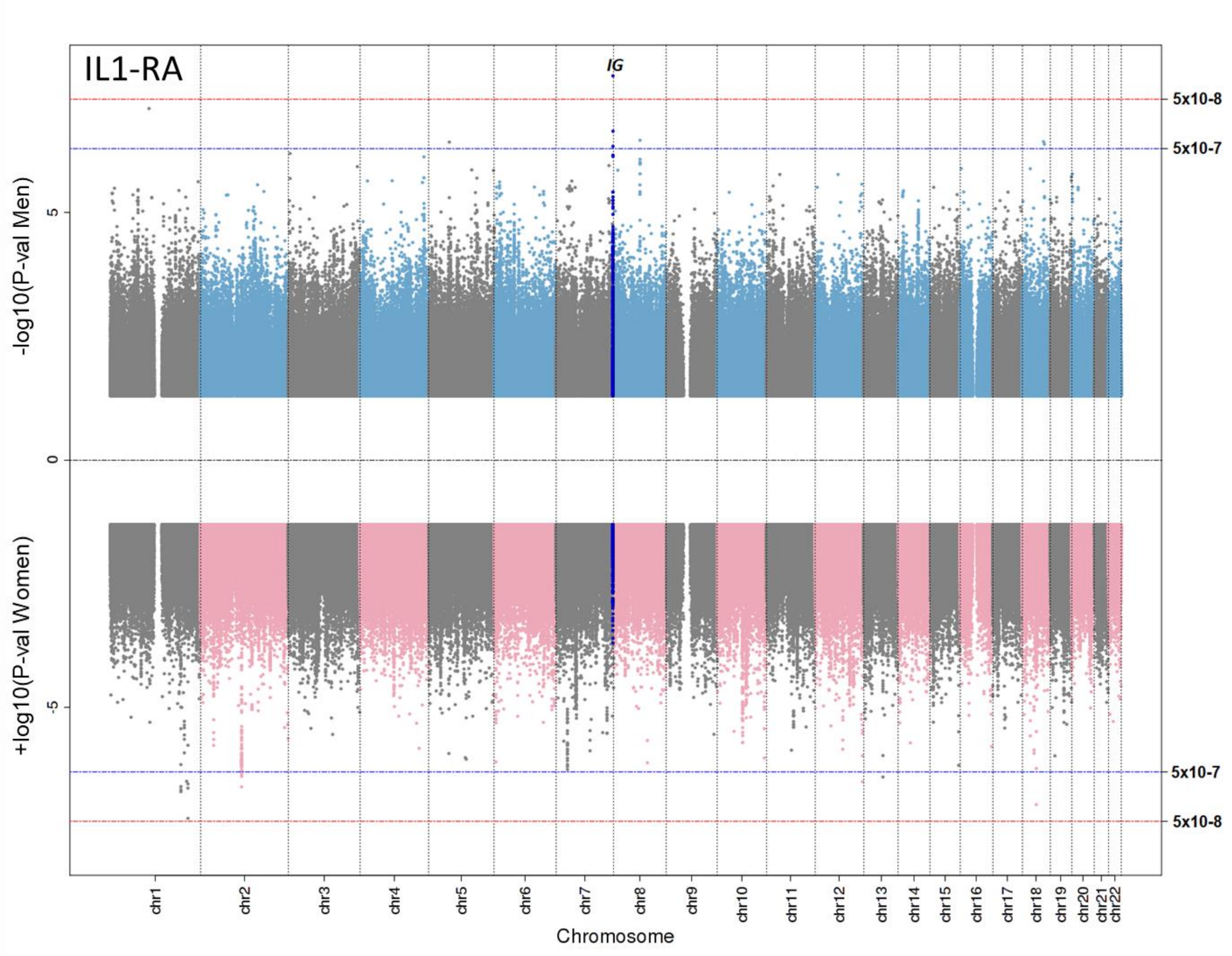
IL1-RA
IG
5x10-8
5x10-7
5x10-7
5x10-8
-log10(P-val Men)
+log10(P-val Women)
5
0
-5
chr1
chr2
chr3
chr4
chr5
chr6
chr7
chr8
chr9
chr10
chr11
chr12
chr13
chr14
chr15
chr16
chr17
chr18
chr19
chr20
chr21
chr22
Chromosome

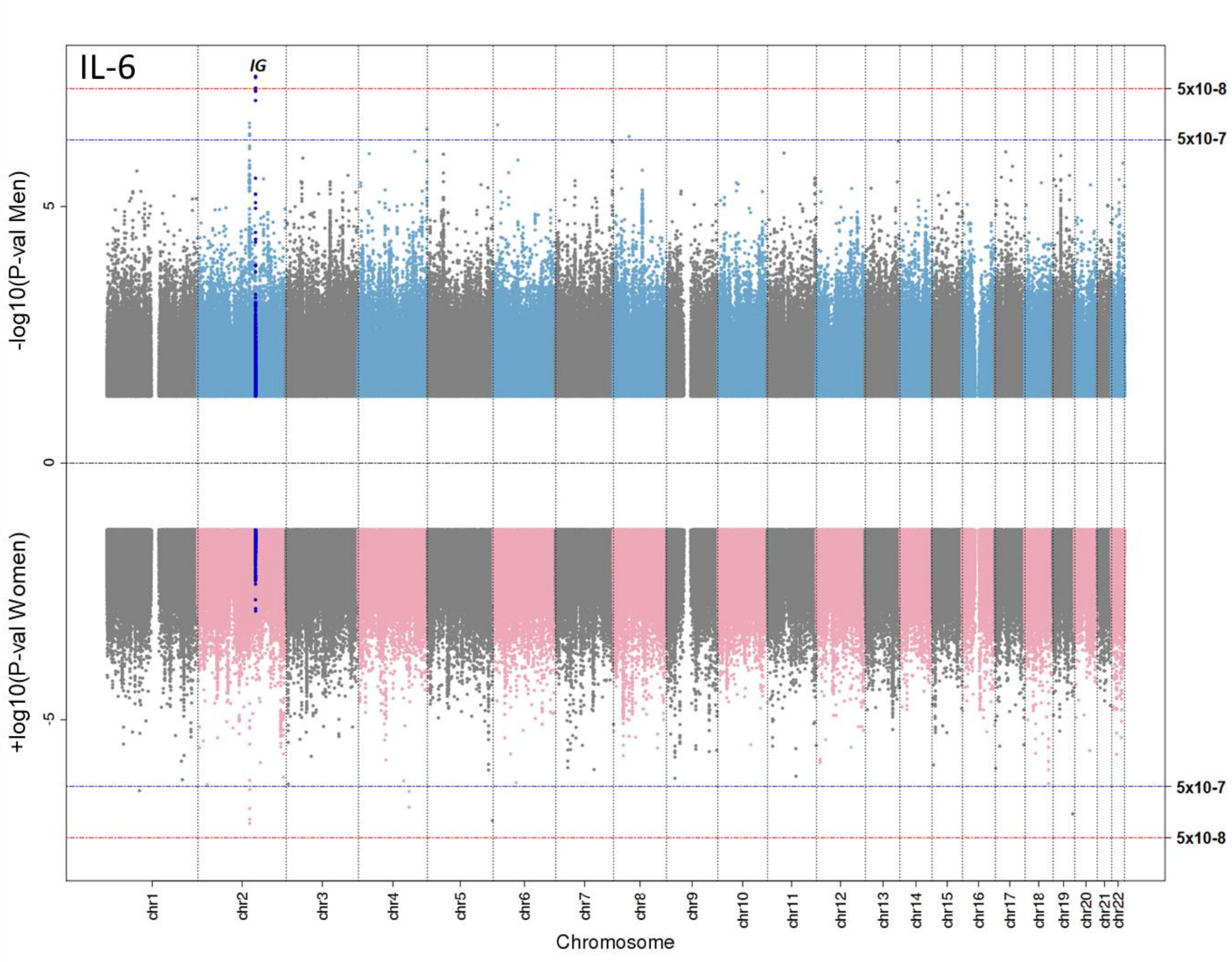
IL-6
IG
5x10-8
5x10-7
5x10-7
5x10-8
-log10(P-val Men)
+log10(P-val Women)
5
0
-5
chr1
chr2
chr3
chr4
chr5
chr6
chr7
chr8
chr9
chr10
chr11
chr12
chr13
chr14
chr15
chr16
chr17
chr18
chr19
chr20
chr21
chr22
Chromosome

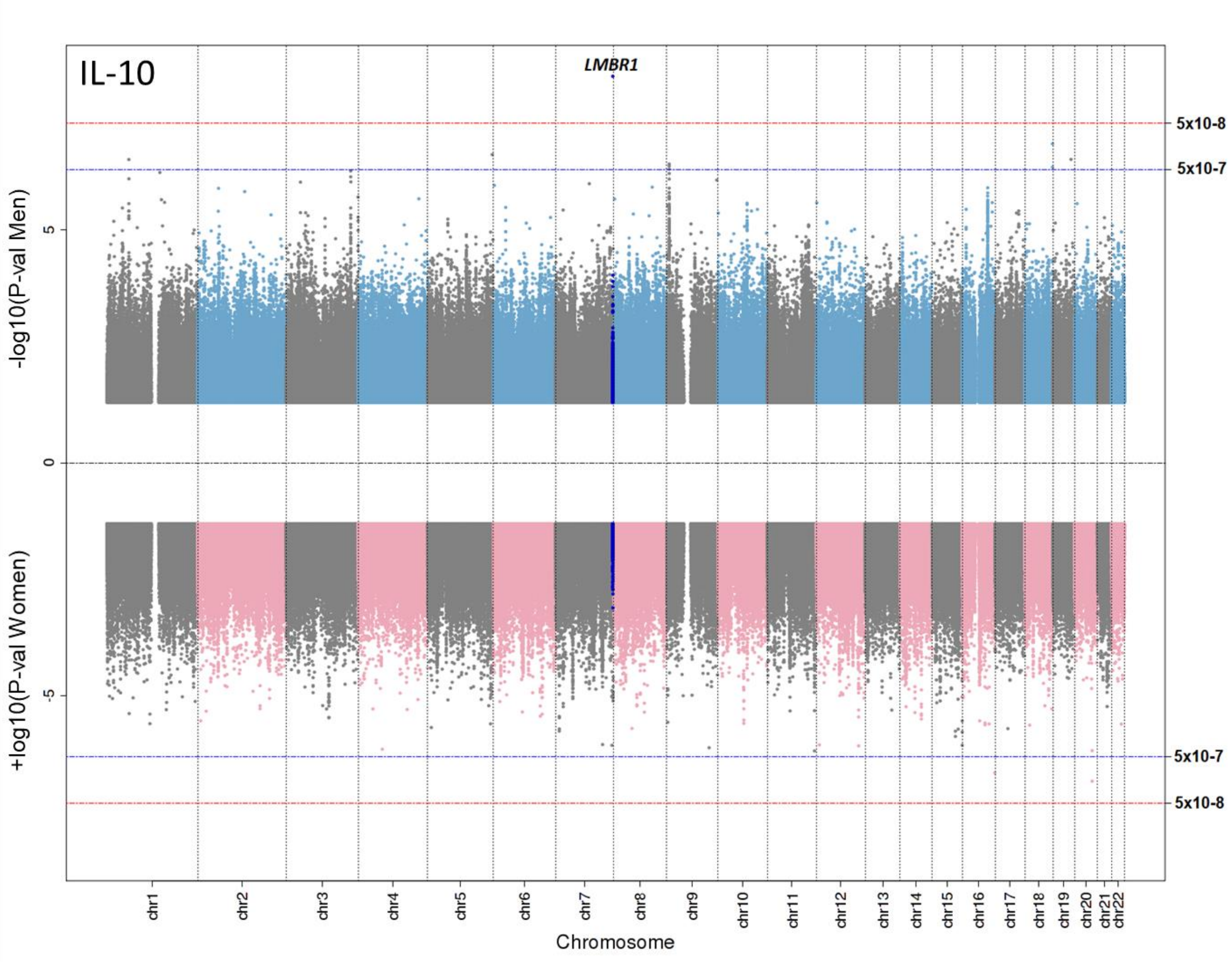
IL-10
LMBR1
5x10-8
5x10-7
-log10(P-val Men)
+log10(P-val Women)
5
0
-5
chr1
chr2
chr3
chr4
chr5
chr6
chr7
chr8
chr9
chr10
chr11
chr12
chr13
chr14
chr15
chr16
chr17
chr18
chr19
chr20
chr21
chr22
Chromosome

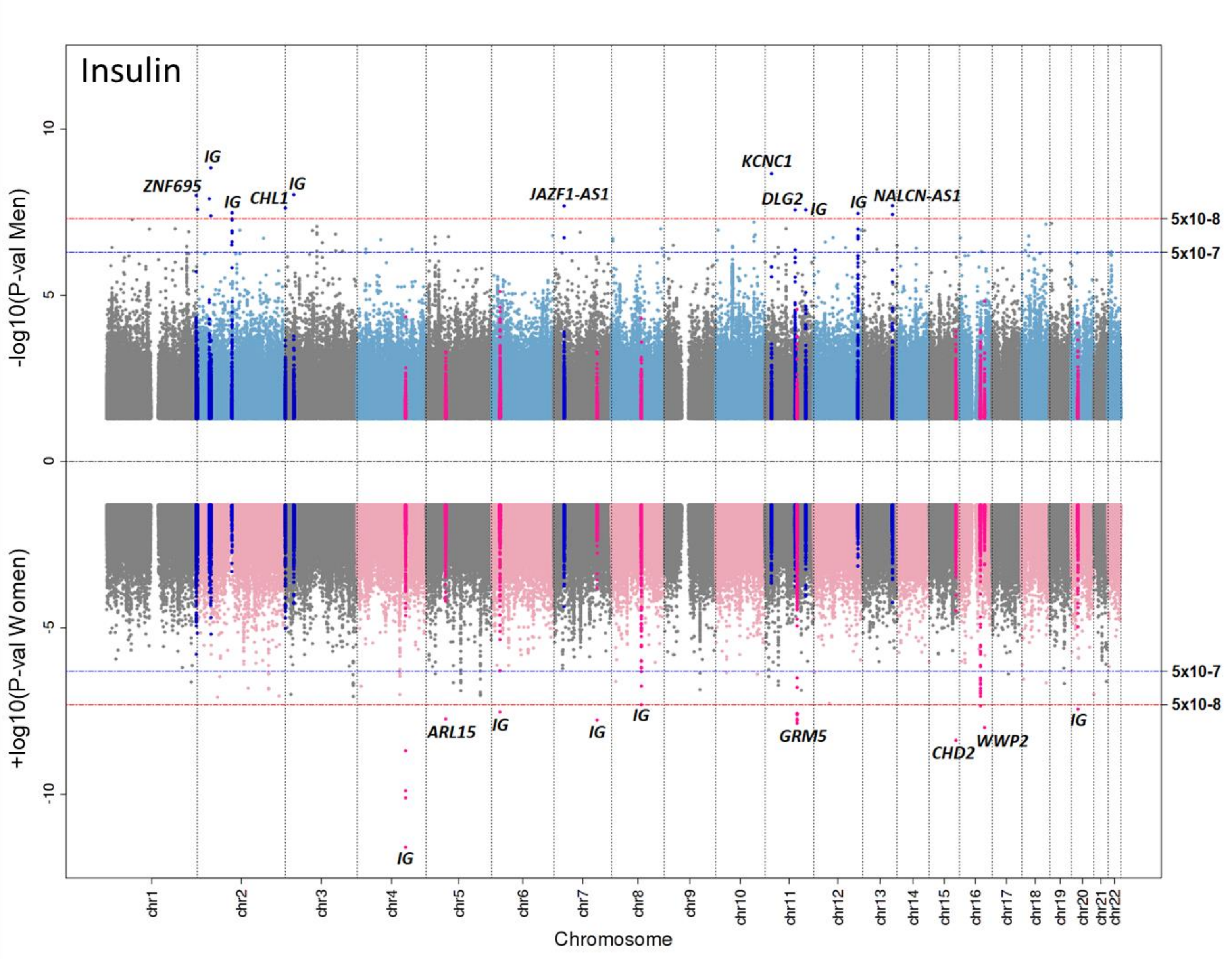
Insulin
-log10(P-val Men)
+log10(P-val Women)
10
5
0
-5
-10
5x10-8
5x10-7
5x10-7
5x10-8
ZNF695
IG
IG
CHL1
IG
JAZF1-AS1
KCNC1
DLG2
IG
IG
NALCN-AS1
ARL15
IG
IG
IG
GRM5
CHD2
WWP2
IG
IG
chr1
chr2
chr3
chr4
chr5
chr6
chr7
chr8
chr9
chr10
chr11
chr12
chr13
chr14
chr15
chr16
chr17
chr18
chr19
chr20
chr21
chr22
Chromosome

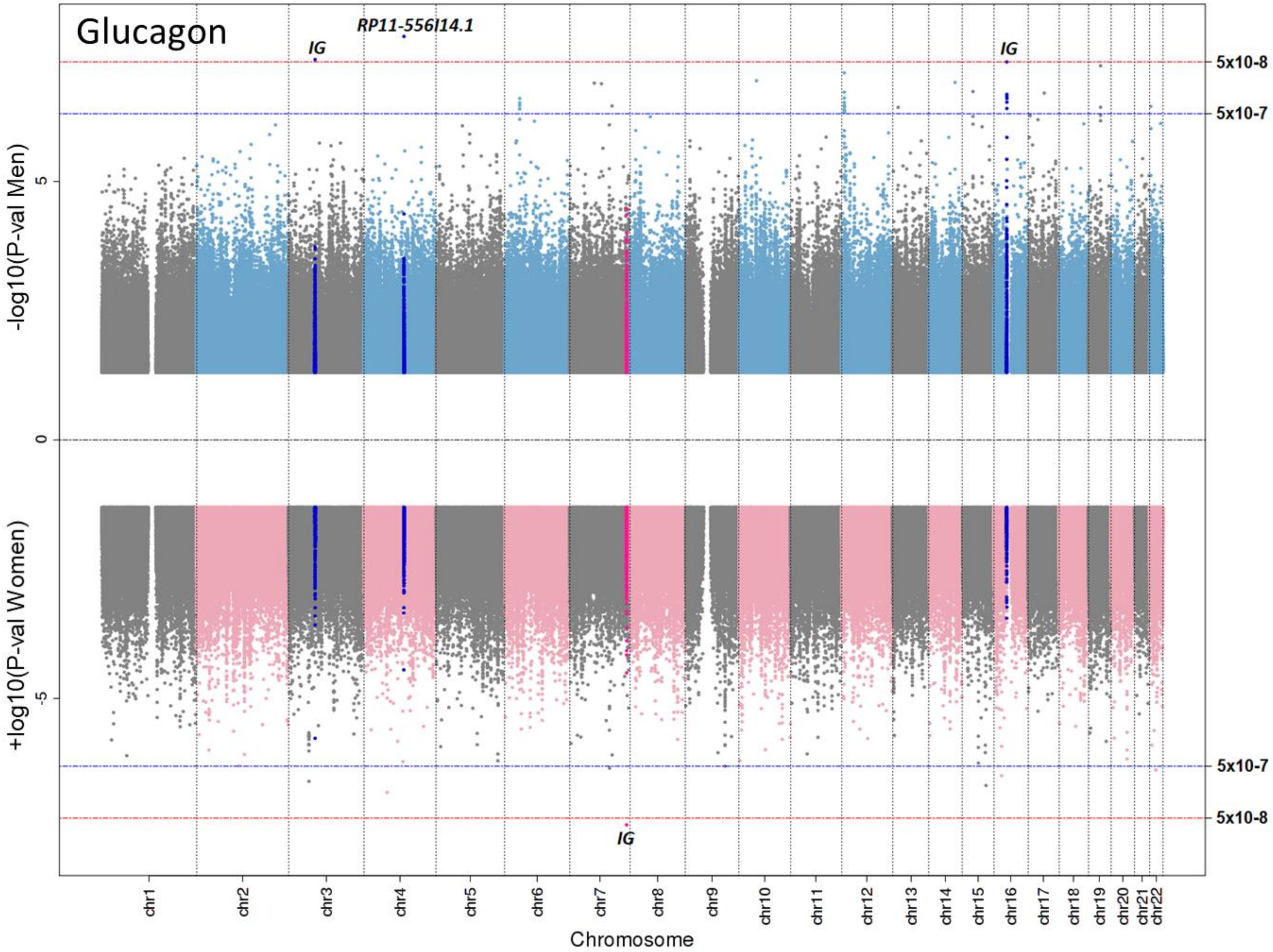
Glucagon
RP11-556I14.1
IG
IG
IG
5x10-8
5x10-7
5x10-7
5x10-8
-log10(P-val Men)
+log10(P-val Women)
5
0
-5
chr1
chr2
chr3
chr4
chr5
chr6
chr7
chr8
chr9
chr10
chr11
chr12
chr13
chr14
chr15
chr16
chr17
chr18
chr19
chr20
chr21
chr22
Chromosome

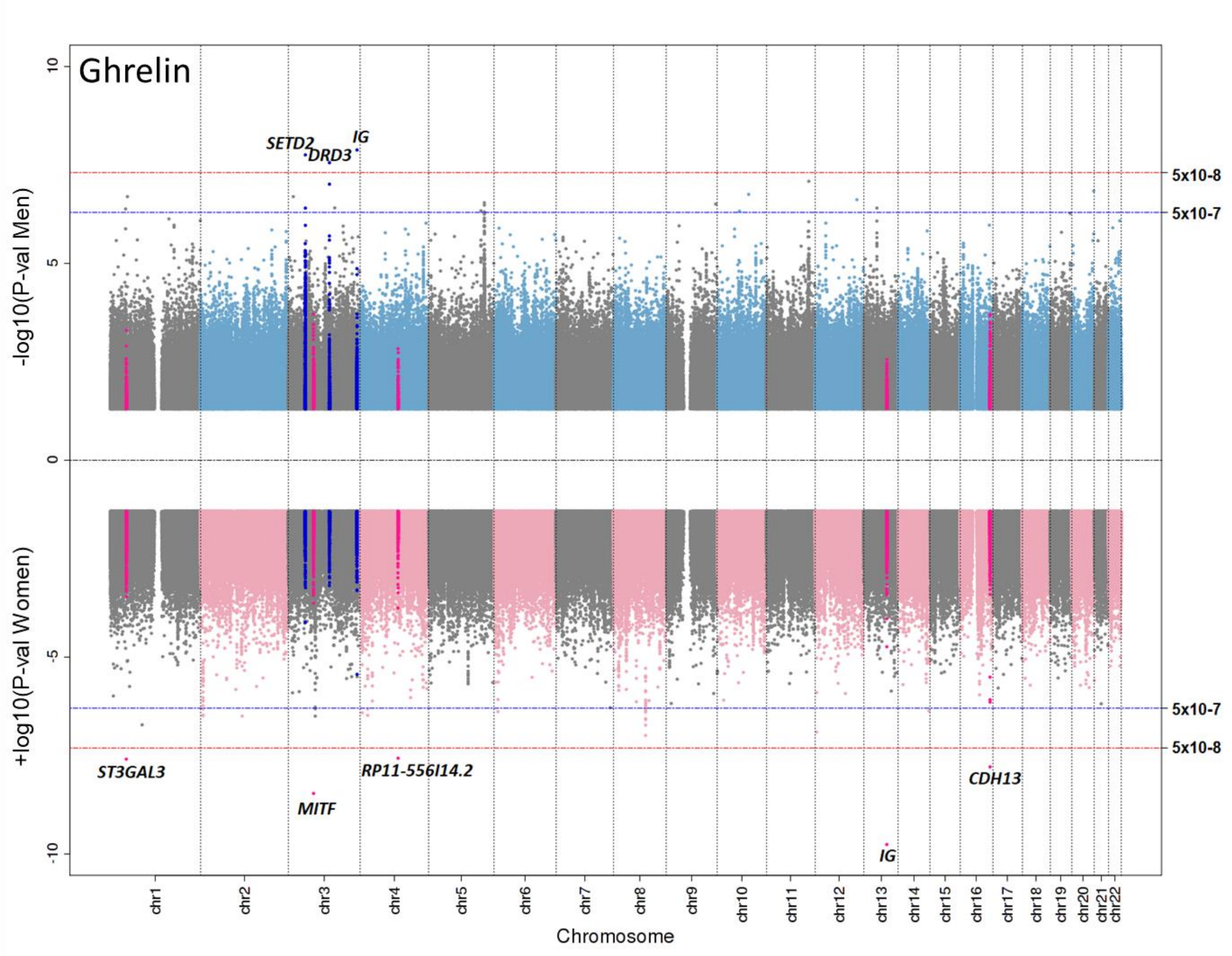
Ghrelin
SETD2
DRD3
IG
5x10-8
5x10-7
5x10-7
5x10-8
ST3GAL3
MITF
RP11-556I14.2
IG
CDH13
-log10(P-val Men)
+log10(P-val Women)
10
5
0
-5
-10
chr1
chr2
chr3
chr4
chr5
chr6
chr7
chr8
chr9
chr10
chr11
chr12
chr13
chr14
chr15
chr16
chr17
chr18
chr19
chr20
chr21
chr22
Chromosome

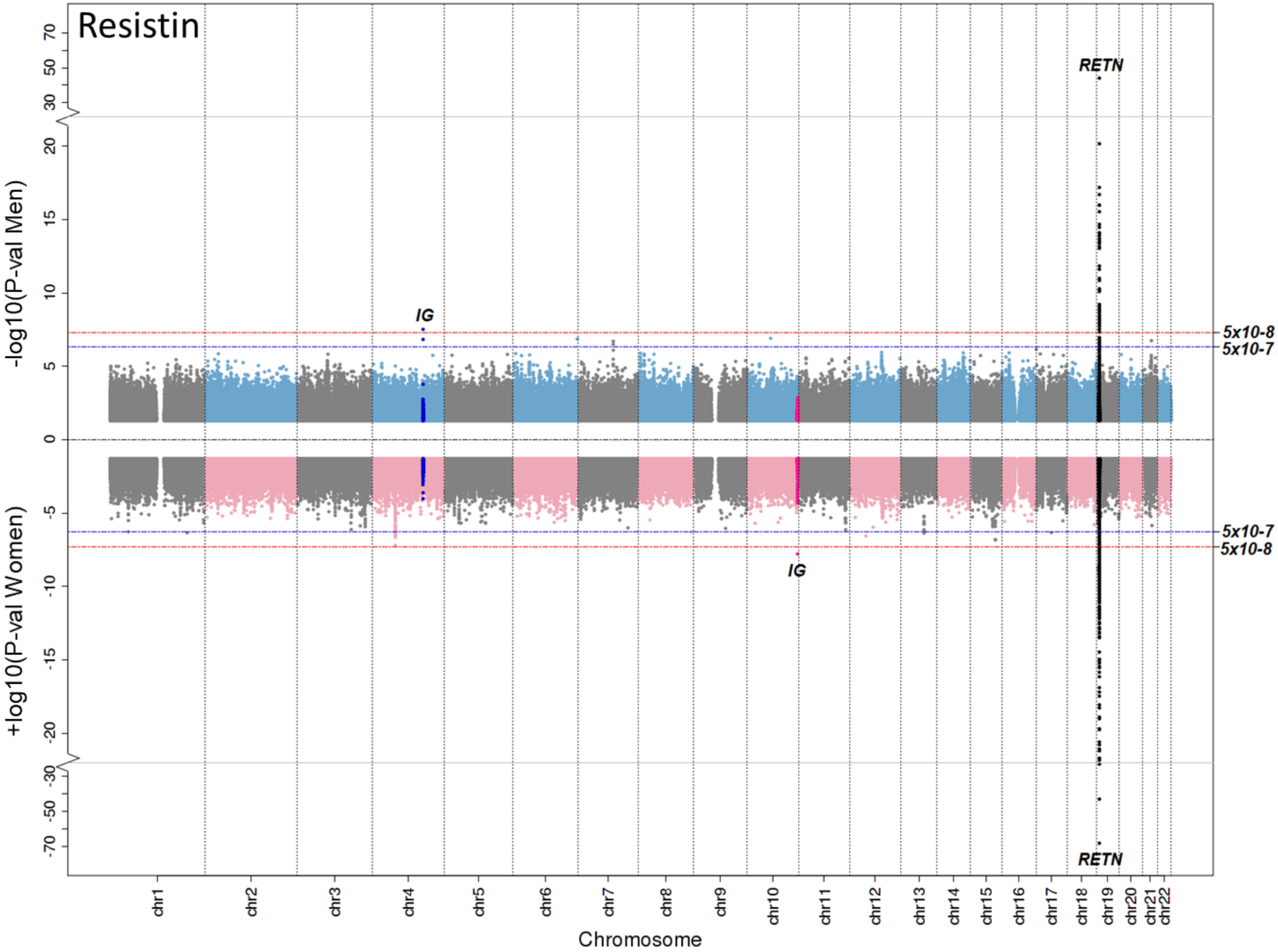
Resistin
RETN
IG
-log10(P-val Men)
+log10(P-val Women)
5x10-8
5x10-7
5x10-7
5x10-8
IG
RETN
chr1
chr2
chr3
chr4
chr5
chr6
chr7
chr8
chr9
chr10
chr11
chr12
chr13
chr14
chr15
chr16
chr17
chr18
chr19
chr20
chr21
chr22
Chromosome

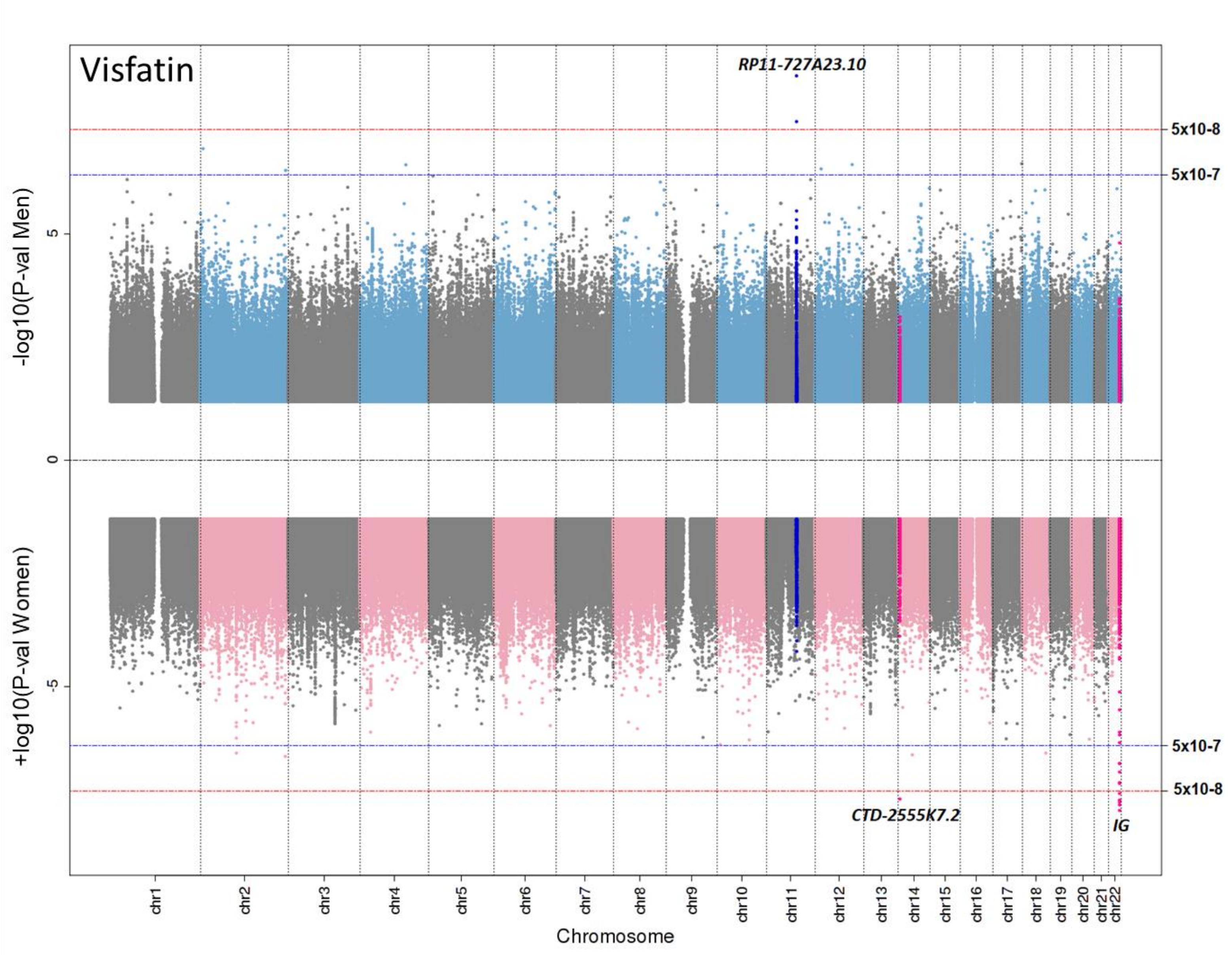
Visfatin
RP11-727A23.10
5x10-8
5x10-7
5
0
-5
5x10-7
5x10-8
CTD-2555K7.2
IG
-log10(P-val Men)
+log10(P-val Women)
chr1
chr2
chr3
chr4
chr5
chr6
chr7
chr8
chr9
chr10
chr11
chr12
chr13
chr14
chr15
chr16
chr17
chr18
chr19
chr20
chr21
chr22
Chromosome

Fig S5: Miami plots for analyses stratified on BMI status for all 13 obesity- and diabetes-related cytokines and hormones. *Lean is BMI < 25 and overweight is BMI ≥ 25. IG = Intergenic. Loci in salmon had a P-value of <5×10^{-8} in lean individuals and loci in yellow had a P-value of <5×10^{-8} among overweight.*

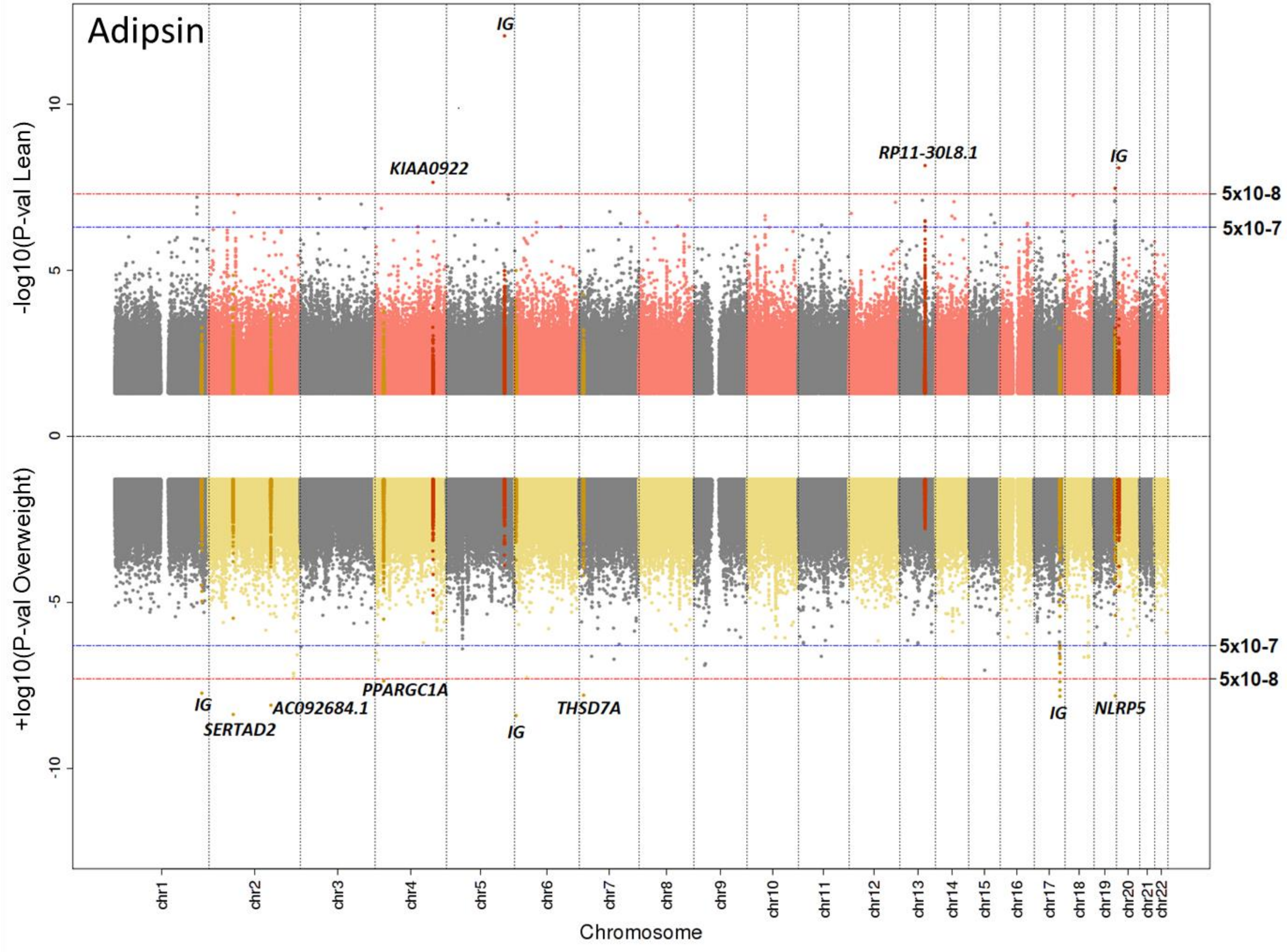

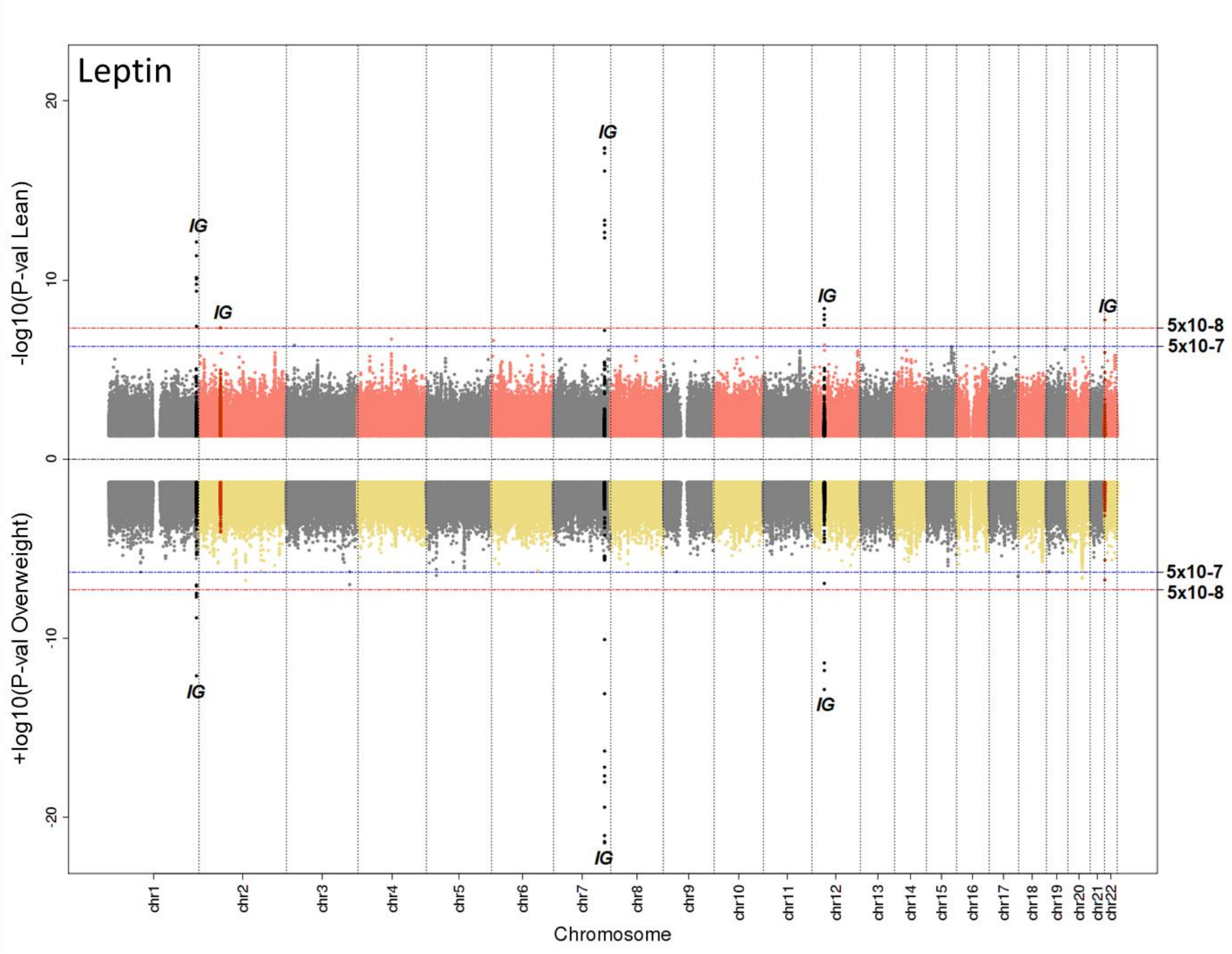
Leptin
-log10(P-val Lean)
+log10(P-val Overweight)
20
10
0
-10
-20
IG
5x10-8
5x10-7
Chromosome
chr1
chr2
chr3
chr4
chr5
chr6
chr7
chr8
chr9
chr10
chr11
chr12
chr13
chr14
chr15
chr16
chr17
chr18
chr19
chr20
chr21
chr22

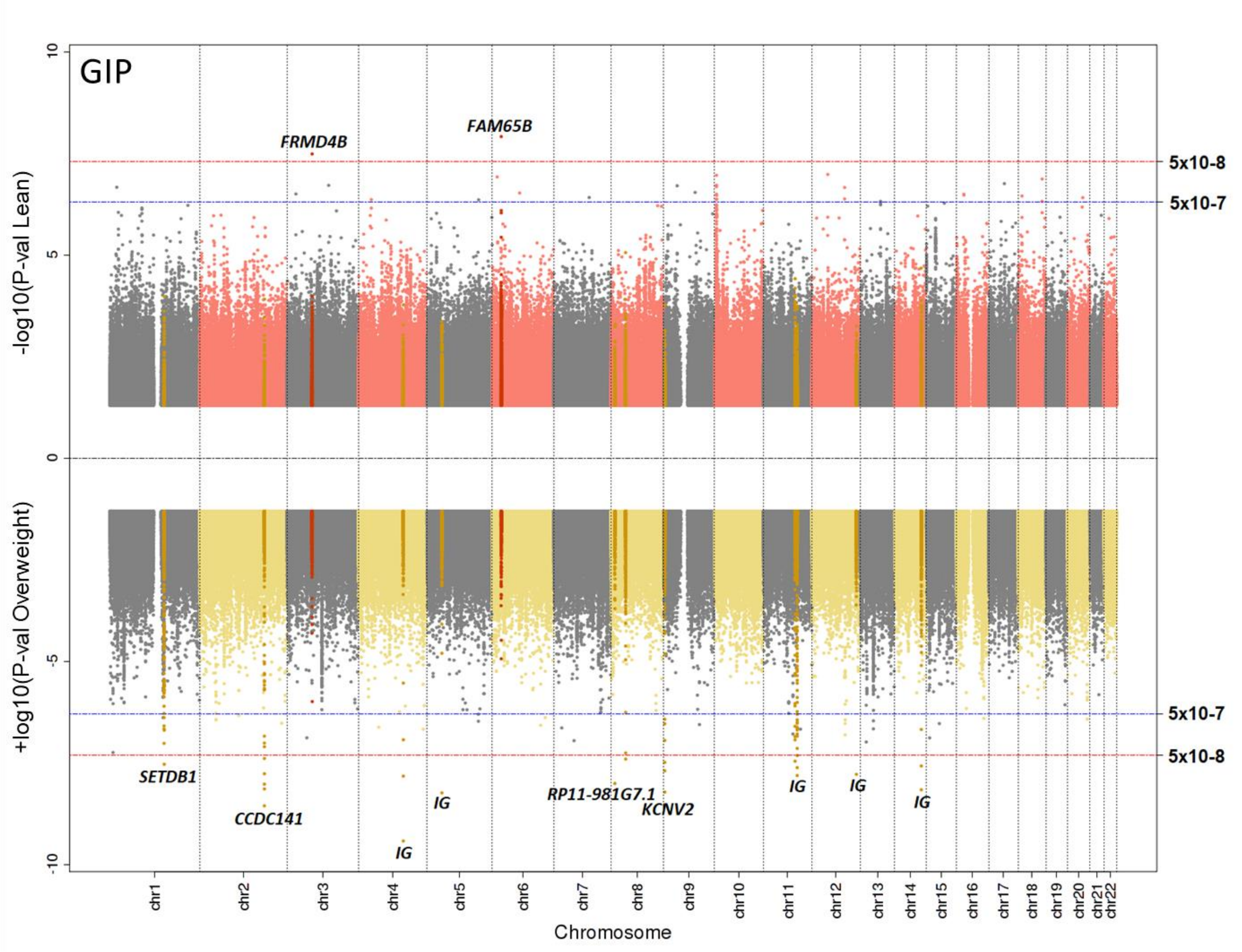
GIP
FRMD4B
FAM65B
5x10-8
5x10-7
-log10(P-val Lean)
+log10(P-val Overweight)
10
5
0
-5
-10
SETDB1
CCDC141
IG
IG
RP11-981G7.1
KCNV2
IG
IG
IG
chr1
chr2
chr3
chr4
chr5
chr6
chr7
chr8
chr9
chr10
chr11
chr12
chr13
chr14
chr15
chr16
chr17
chr18
chr19
chr20
chr21
chr22
Chromosome

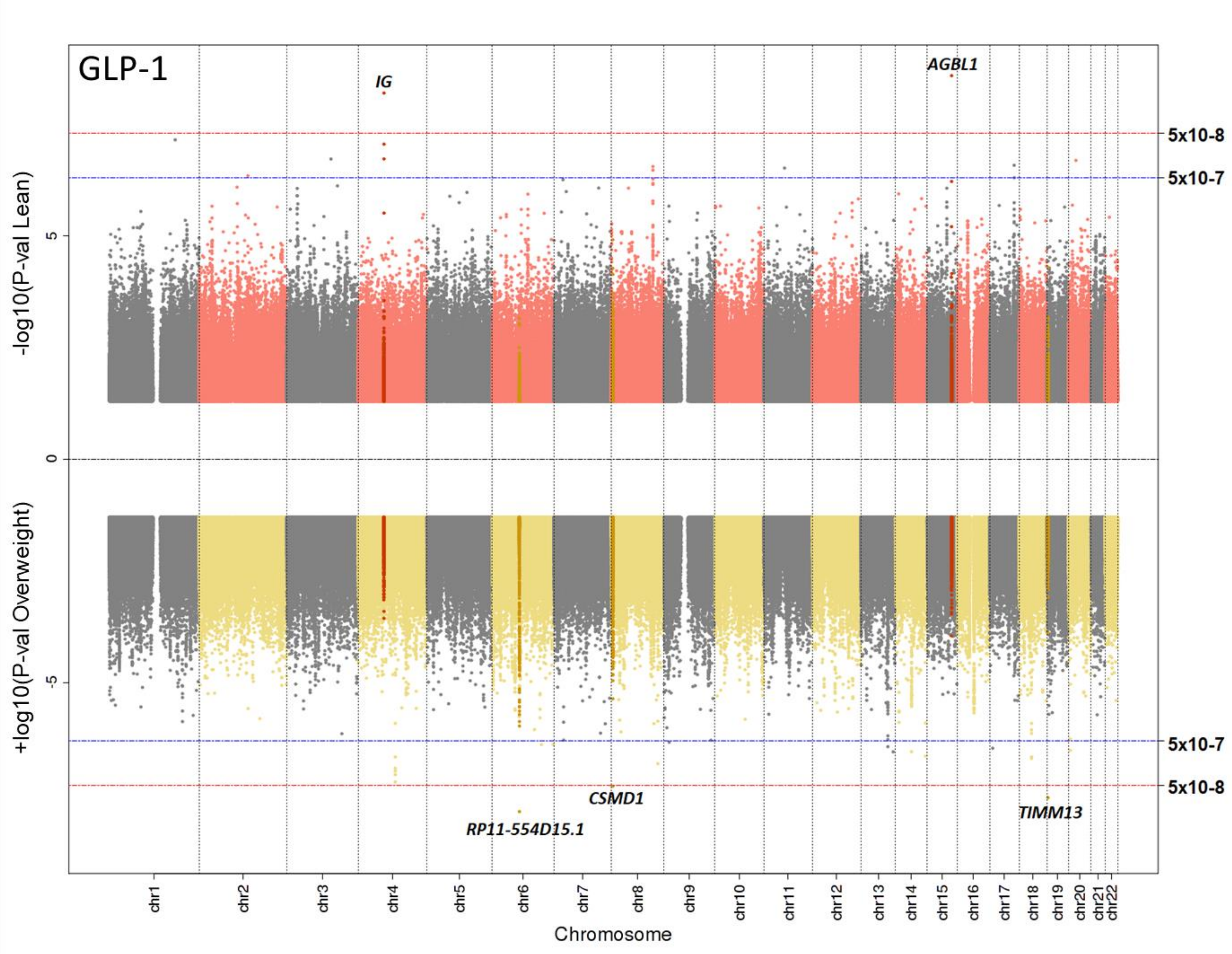

GLP-1
IG
AGBL1
5x10-8
5x10-7
-log10(P-val Lean)
+log10(P-val Overweight)
5
0
-5
CSMD1
RP11-554D15.1
TIMM13
chr1
chr2
chr3
chr4
chr5
chr6
chr7
chr8
chr9
chr10
chr11
chr12
chr13
chr14
chr15
chr16
chr17
chr18
chr19
chr20
chr21
chr22
Chromosome

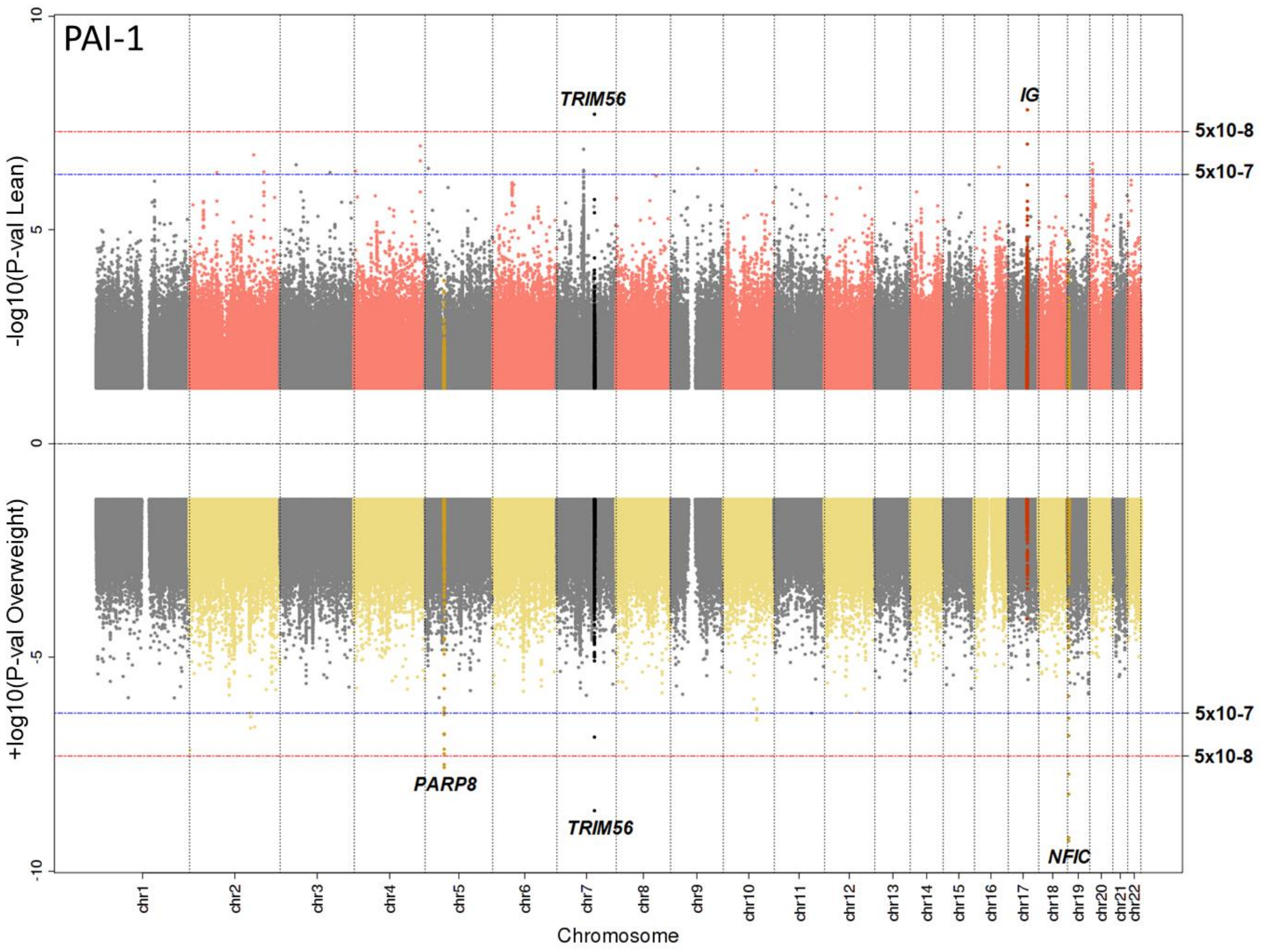
PAI-1
TRIM56
IG
5x10-8
5x10-7
-log10(P-val Lean)
+log10(P-val Overweight)
PARP8
TRIM56
NFIC
10
5
0
-5
-10
chr1
chr2
chr3
chr4
chr5
chr6
chr7
chr8
chr9
chr10
chr11
chr12
chr13
chr14
chr15
chr16
chr17
chr18
chr19
chr20
chr21
chr22
Chromosome

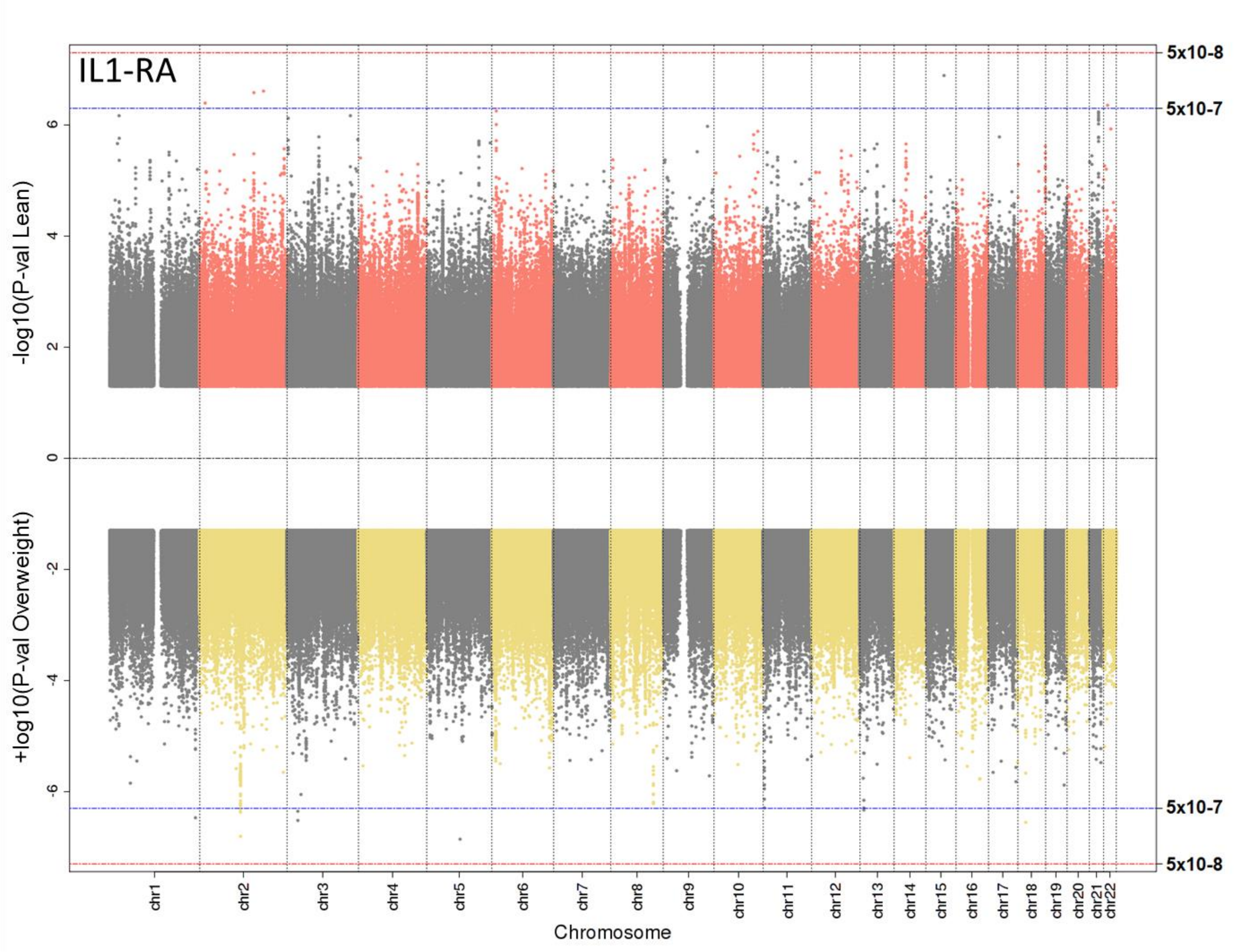
IL1-RA
5x10-8
5x10-7
5x10-7
5x10-8
-log10(P-val Lean)
+log10(P-val Overweight)
6
4
2
0
-2
-4
-6
chr1
chr2
chr3
chr4
chr5
chr6
chr7
chr8
chr9
chr10
chr11
chr12
chr13
chr14
chr15
chr16
chr17
chr18
chr19
chr20
chr21
chr22
Chromosome

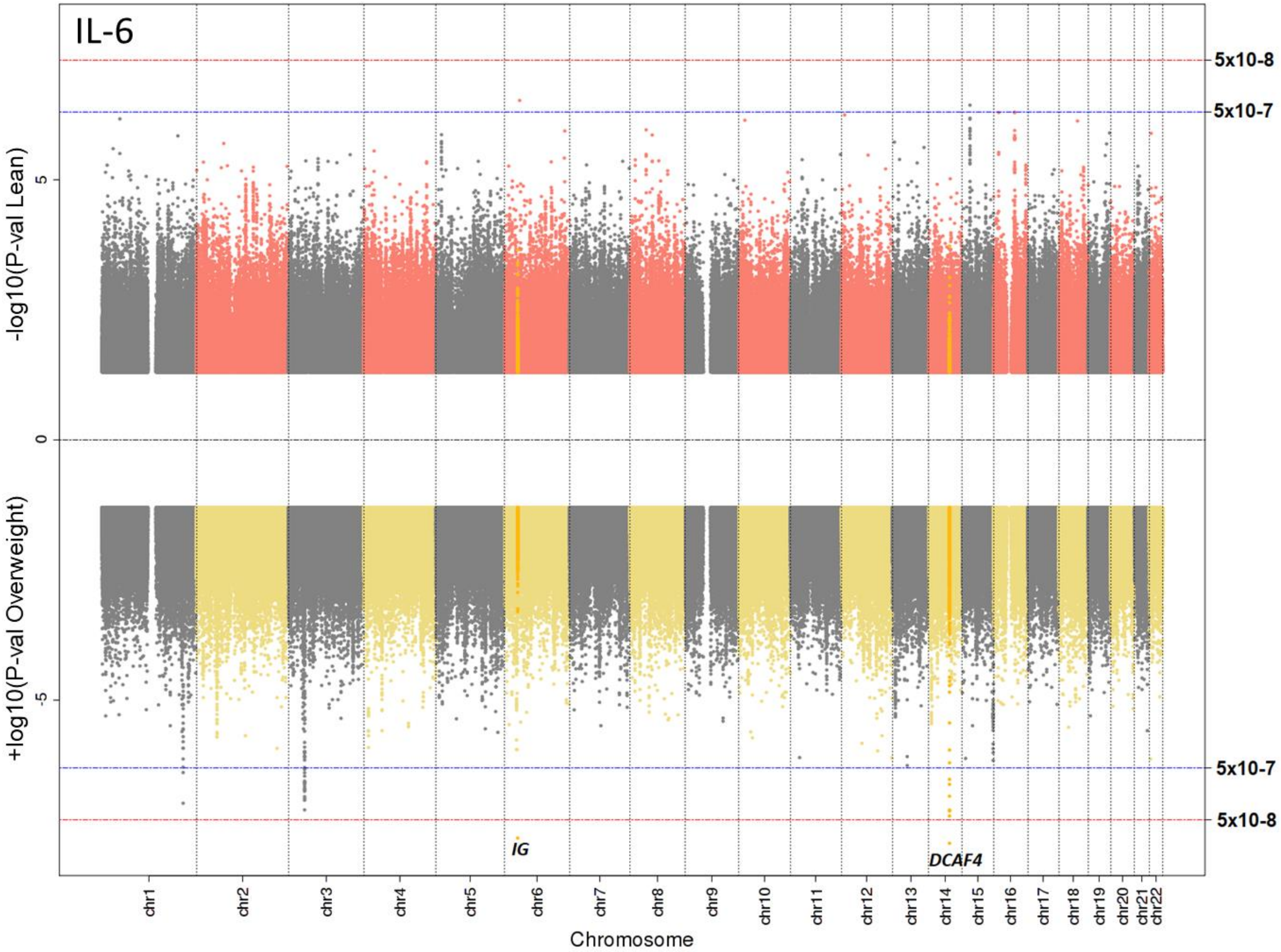
IL-6
5x10-8
5x10-7
5x10-7
5x10-8
-log10(P-val Lean)
+log10(P-val Overweight)
5
0
-5
IG
DCAF4
chr1
chr2
chr3
chr4
chr5
chr6
chr7
chr8
chr9
chr10
chr11
chr12
chr13
chr14
chr15
chr16
chr17
chr18
chr19
chr20
chr21
chr22
Chromosome

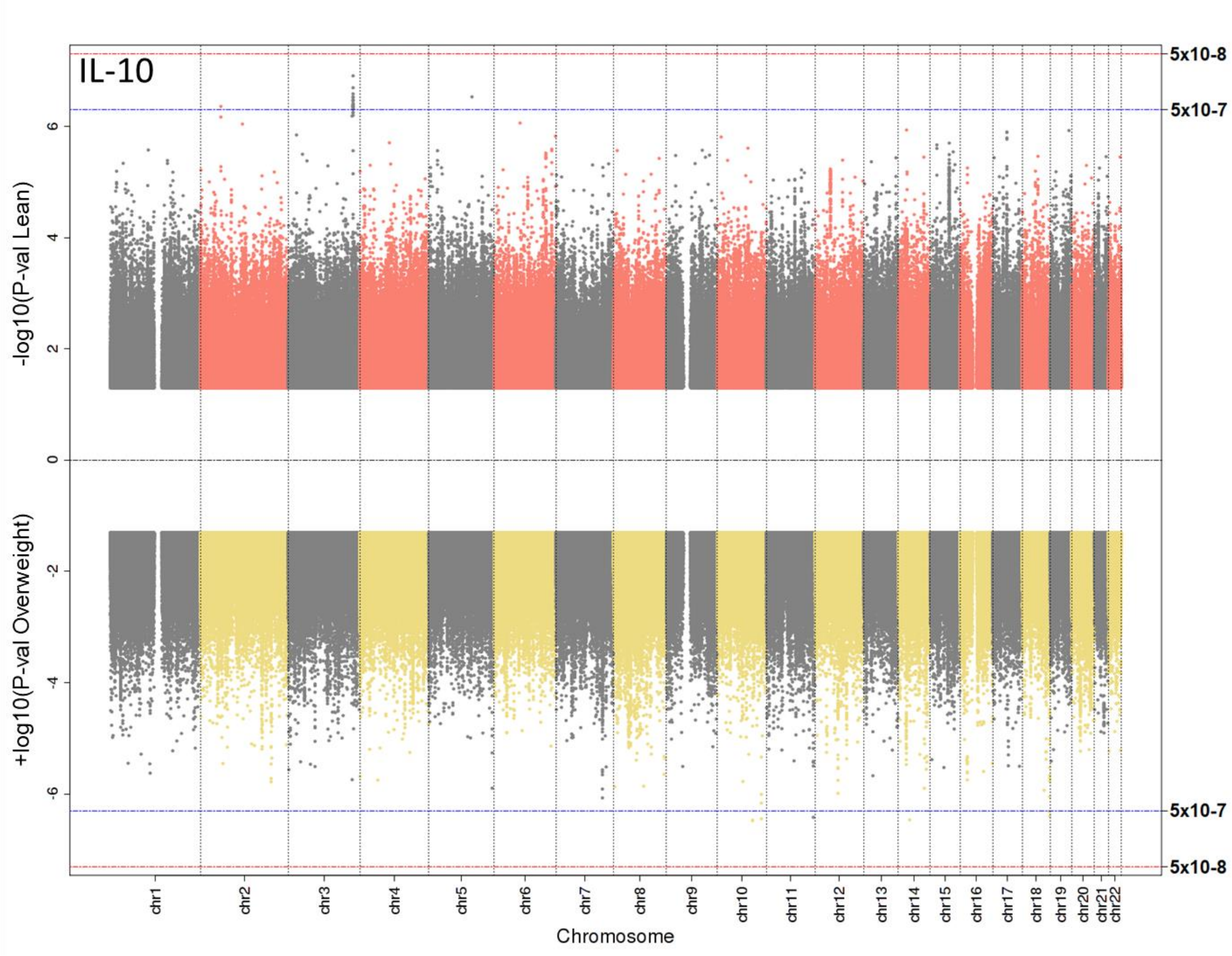

IL-10
-log10(P-val Lean)
+log10(P-val Overweight)
5x10-8
5x10-7
5x10-7
5x10-8
6
4
2
0
-2
-4
-6
chr1
chr2
chr3
chr4
chr5
chr6
chr7
chr8
chr9
chr10
chr11
chr12
chr13
chr14
chr15
chr16
chr17
chr18
chr19
chr20
chr21
chr22
Chromosome

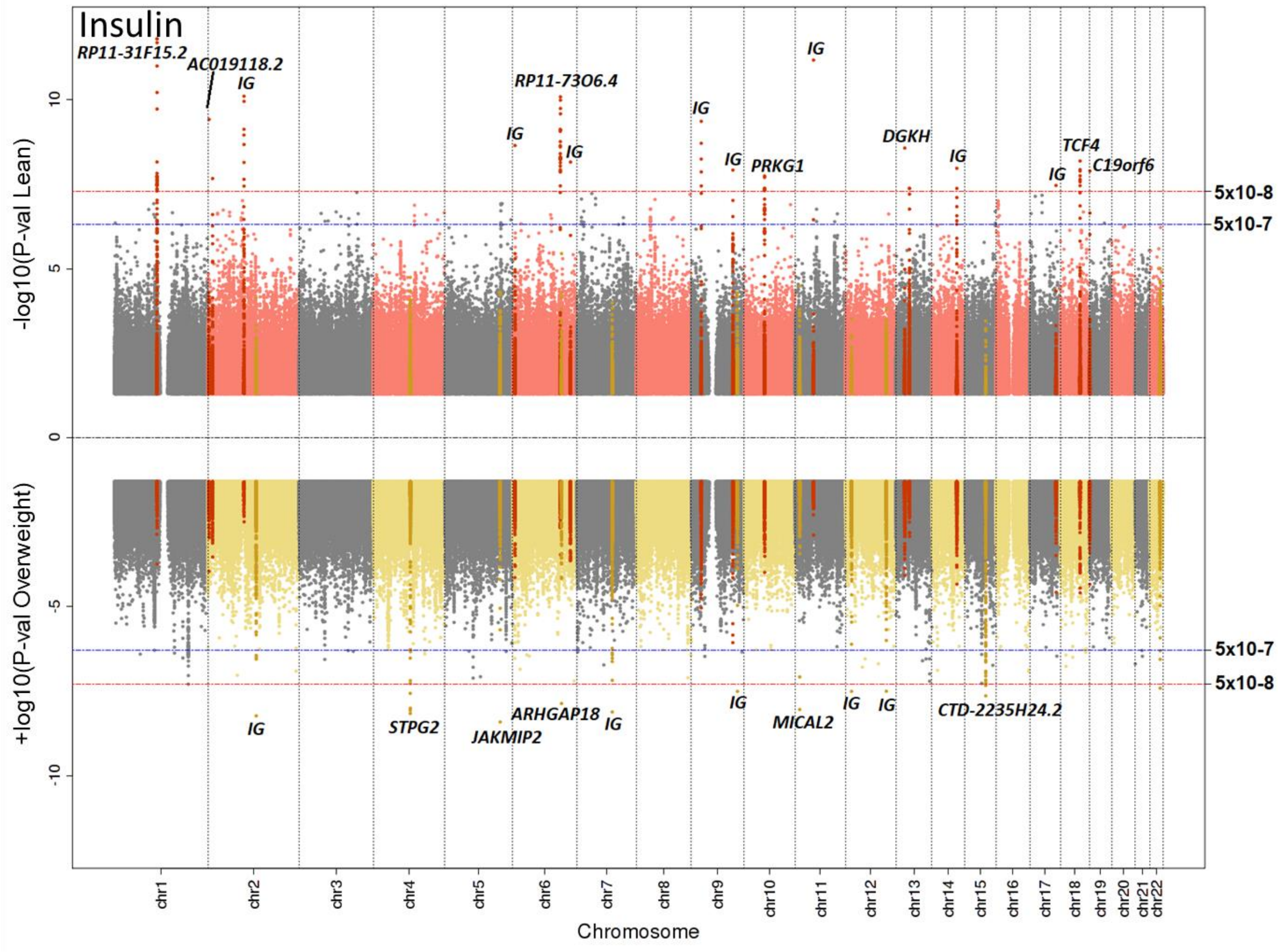
Insulin
RP11-31F15.2
AC019118.2
IG
RP11-73O6.4
IG
IG
IG
IG
PRKG1
IG
DGKH
IG
TCF4
IG
C19orf6
5x10-8
5x10-7
-log10(P-val Lean)
+log10(P-val Overweight)
5x10-7
5x10-8
IG
STPG2
ARHGAP18
JAKMIP2
IG
IG
MICAL2
IG
IG
CTD-2235H24.2
Chromosome
chr1
chr2
chr3
chr4
chr5
chr6
chr7
chr8
chr9
chr10
chr11
chr12
chr13
chr14
chr15
chr16
chr17
chr18
chr19
chr20
chr21
chr22

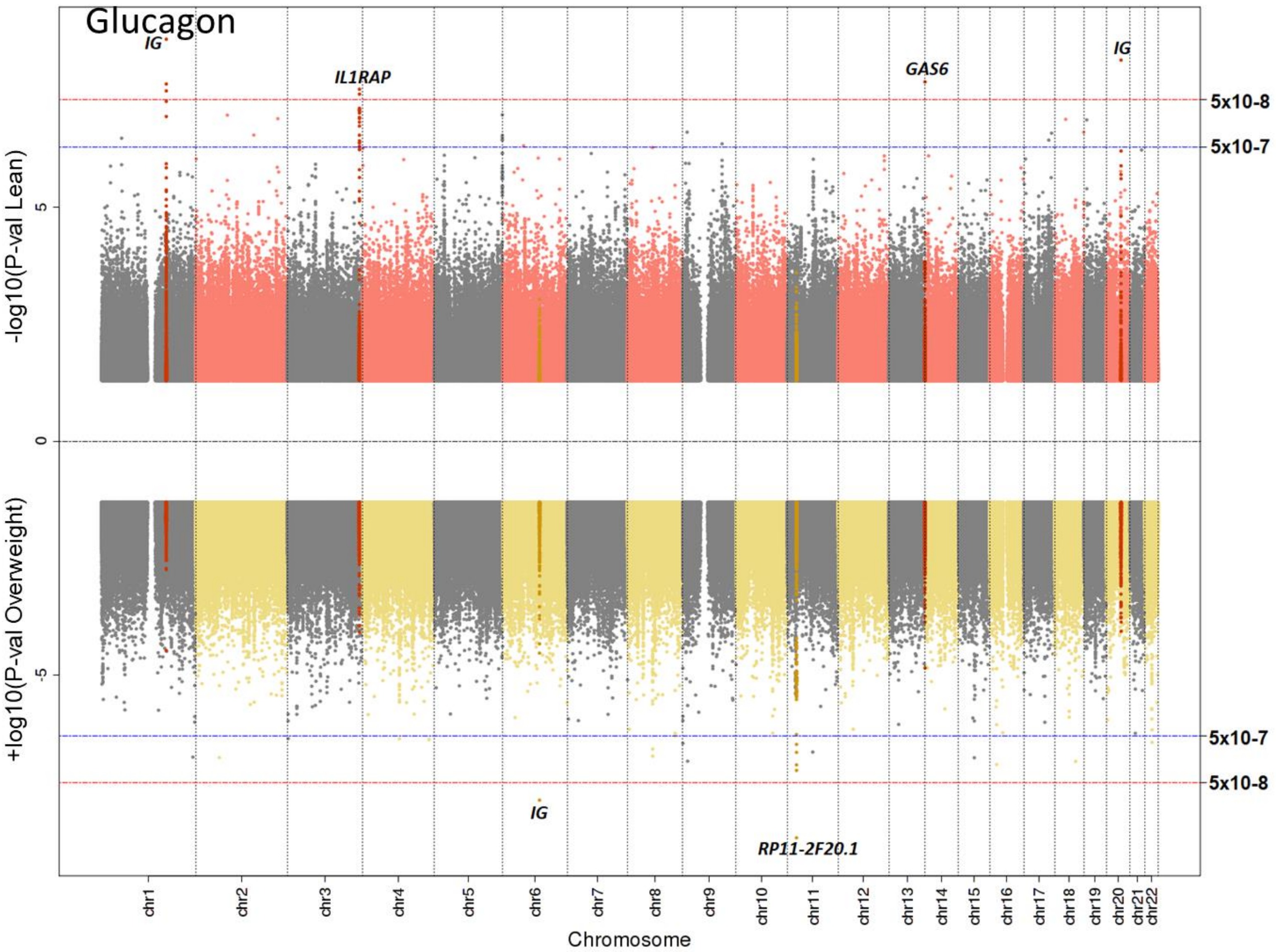
Glucagon
IG
IL1RAP
GAS6
IG
5x10-8
5x10-7
5x10-7
5x10-8
IG
RP11-2F20.1
-log10(P-val Lean)
+log10(P-val Overweight)
5
0
-5
chr1
chr2
chr3
chr4
chr5
chr6
chr7
chr8
chr9
chr10
chr11
chr12
chr13
chr14
chr15
chr16
chr17
chr18
chr19
chr20
chr21
chr22
Chromosome

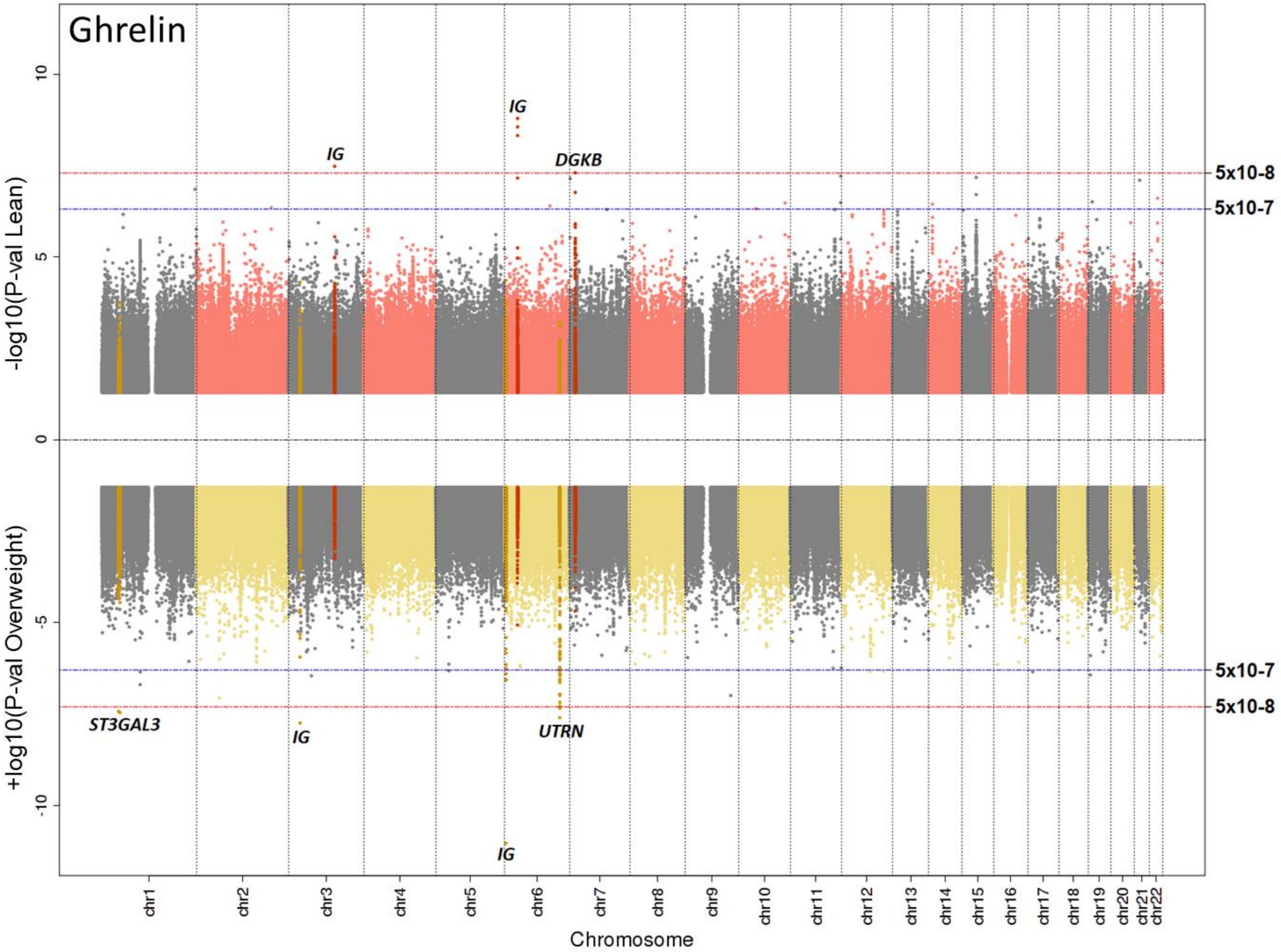

Ghrelin
-log10(P-val Lean)
+log10(P-val Overweight)
10
5
0
-5
-10
IG
IG
DGKB
5x10-8
5x10-7
5x10-7
5x10-8
ST3GAL3
IG
UTRN
IG
chr1
chr2
chr3
chr4
chr5
chr6
chr7
chr8
chr9
chr10
chr11
chr12
chr13
chr14
chr15
chr16
chr17
chr18
chr19
chr20
chr21
chr22
Chromosome

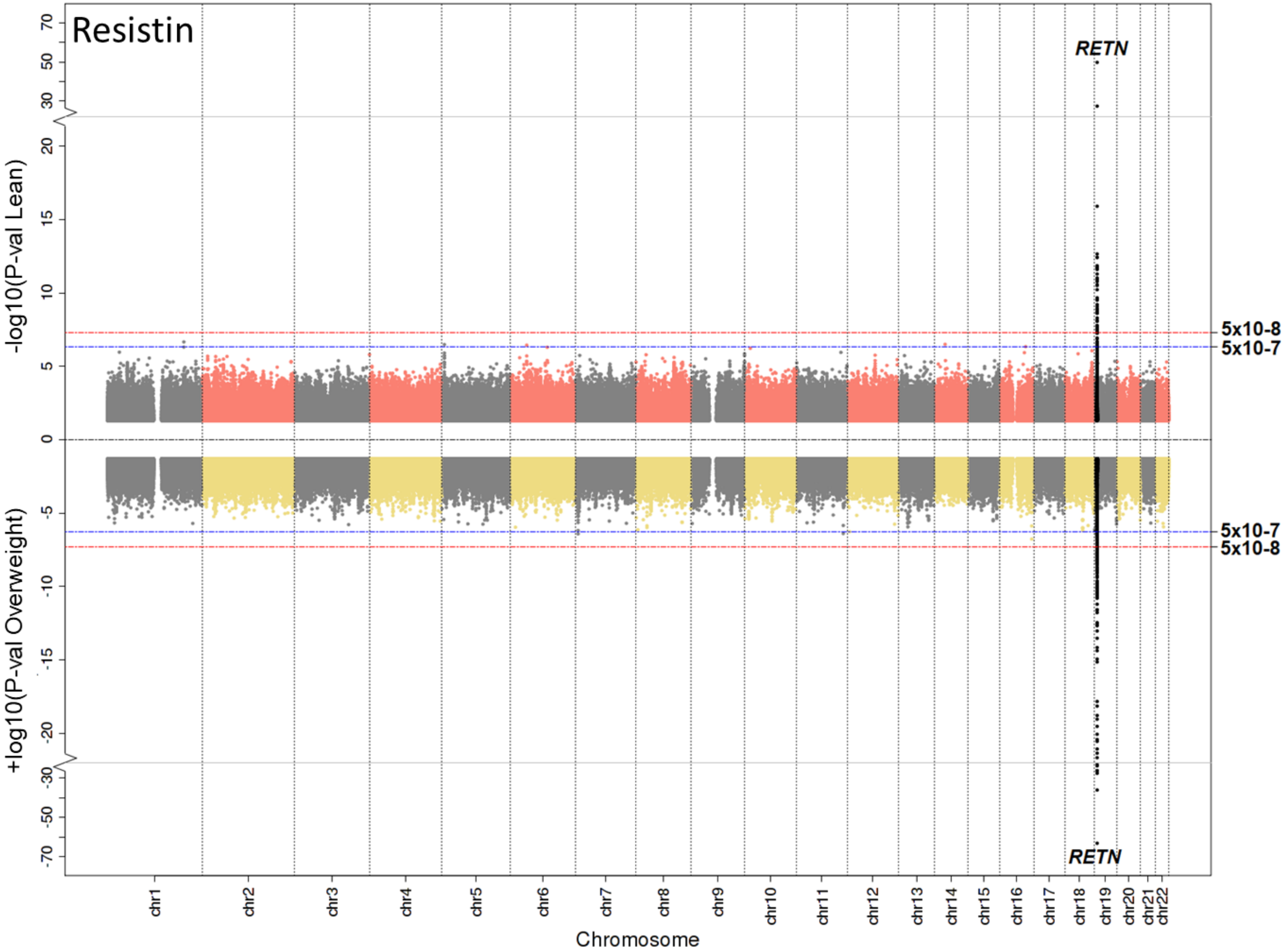

Resistin
RETN
-log10(P-val Lean)
+log10(P-val Overweight)
5x10-8
5x10-7
5x10-7
5x10-8
RETN
chr1
chr2
chr3
chr4
chr5
chr6
chr7
chr8
chr9
chr10
chr11
chr12
chr13
chr14
chr15
chr16
chr17
chr18
chr19
chr20
chr21
chr22
Chromosome

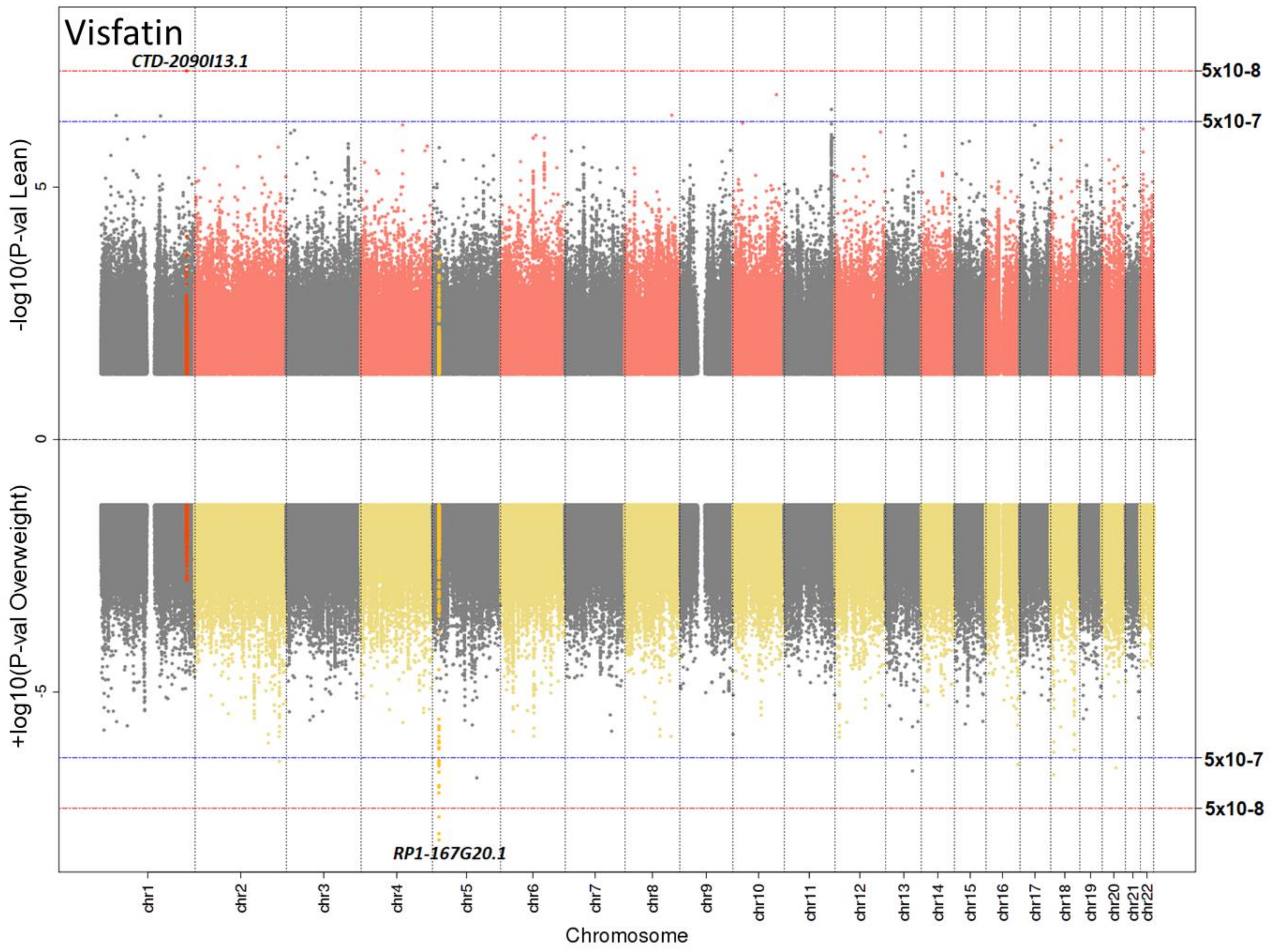
Visfatin
CTD-2090I13.1
RP1-167G20.1
5x10-8
5x10-7
5x10-7
5x10-8
-log10(P-val Lean)
+log10(P-val Overweight)
5
0
-5
chr1
chr2
chr3
chr4
chr5
chr6
chr7
chr8
chr9
chr10
chr11
chr12
chr13
chr14
chr15
chr16
chr17
chr18
chr19
chr20
chr21
chr22
Chromosome

Fig S6: Miami plots for T2D status-stratified analyses for all 13 obesity- and diabetes-related cytokines and hormones.

IG = Intergenic. Loci in green had a P-value of <5×10^{-8} in T2D controls and loci in purple had a P-value of <5×10^{-8} in T2D cases.

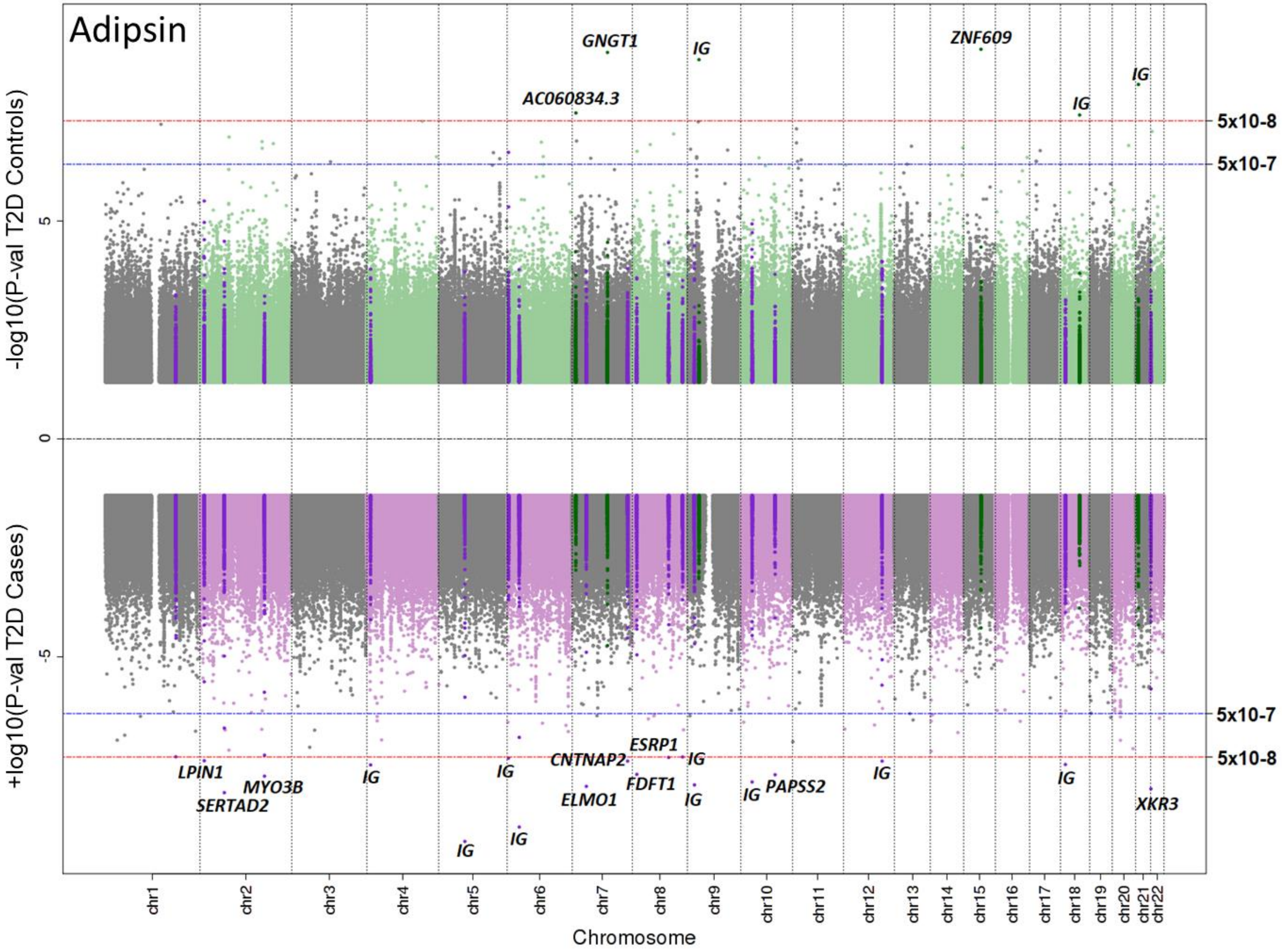

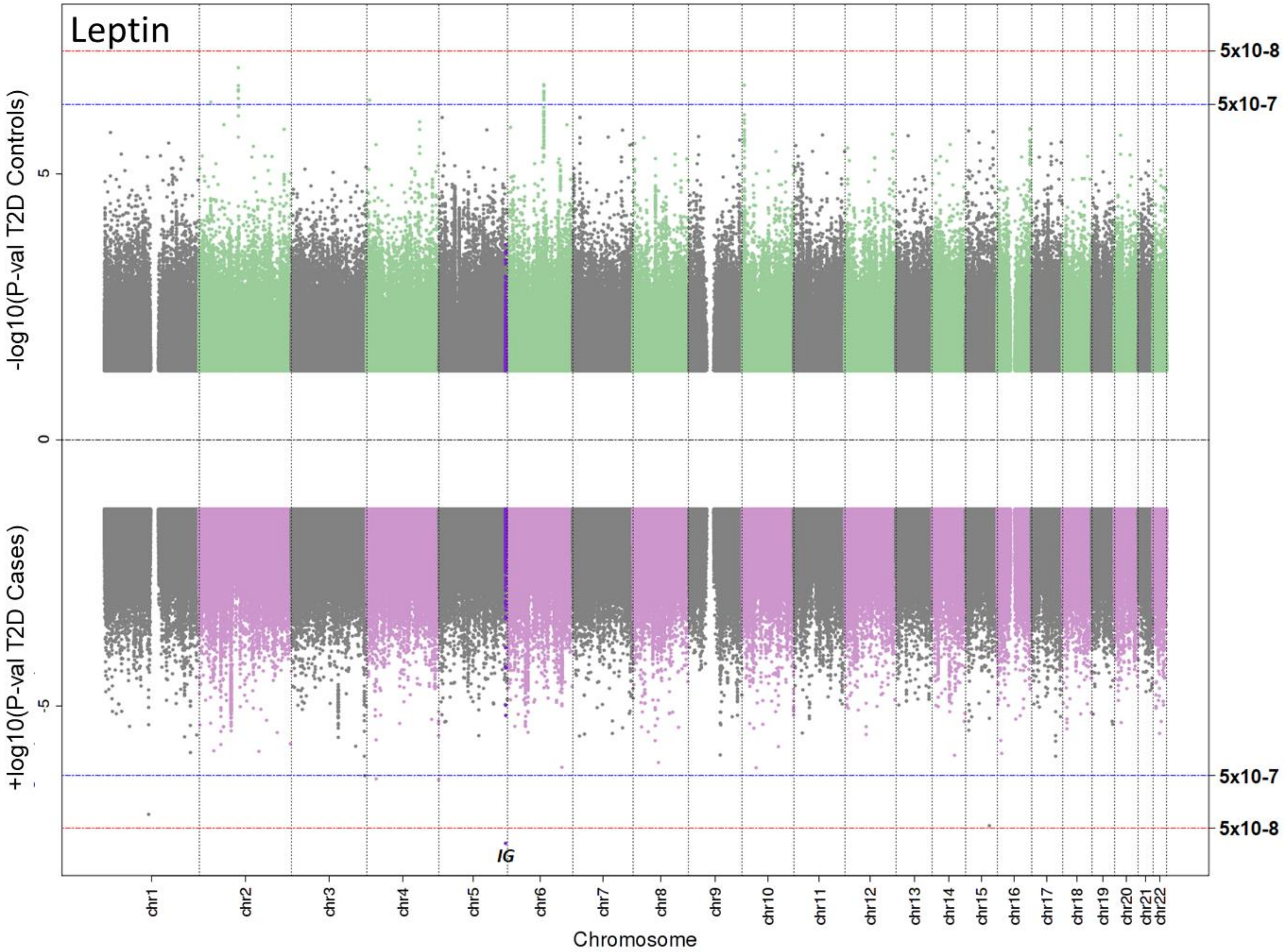
Leptin
5x10-8
5x10-7
5x10-7
5x10-8
-log10(P-val T2D Controls)
+log10(P-val T2D Cases)
5
0
-5
IG
chr1
chr2
chr3
chr4
chr5
chr6
chr7
chr8
chr9
chr10
chr11
chr12
chr13
chr14
chr15
chr16
chr17
chr18
chr19
chr20
chr21
chr22
Chromosome

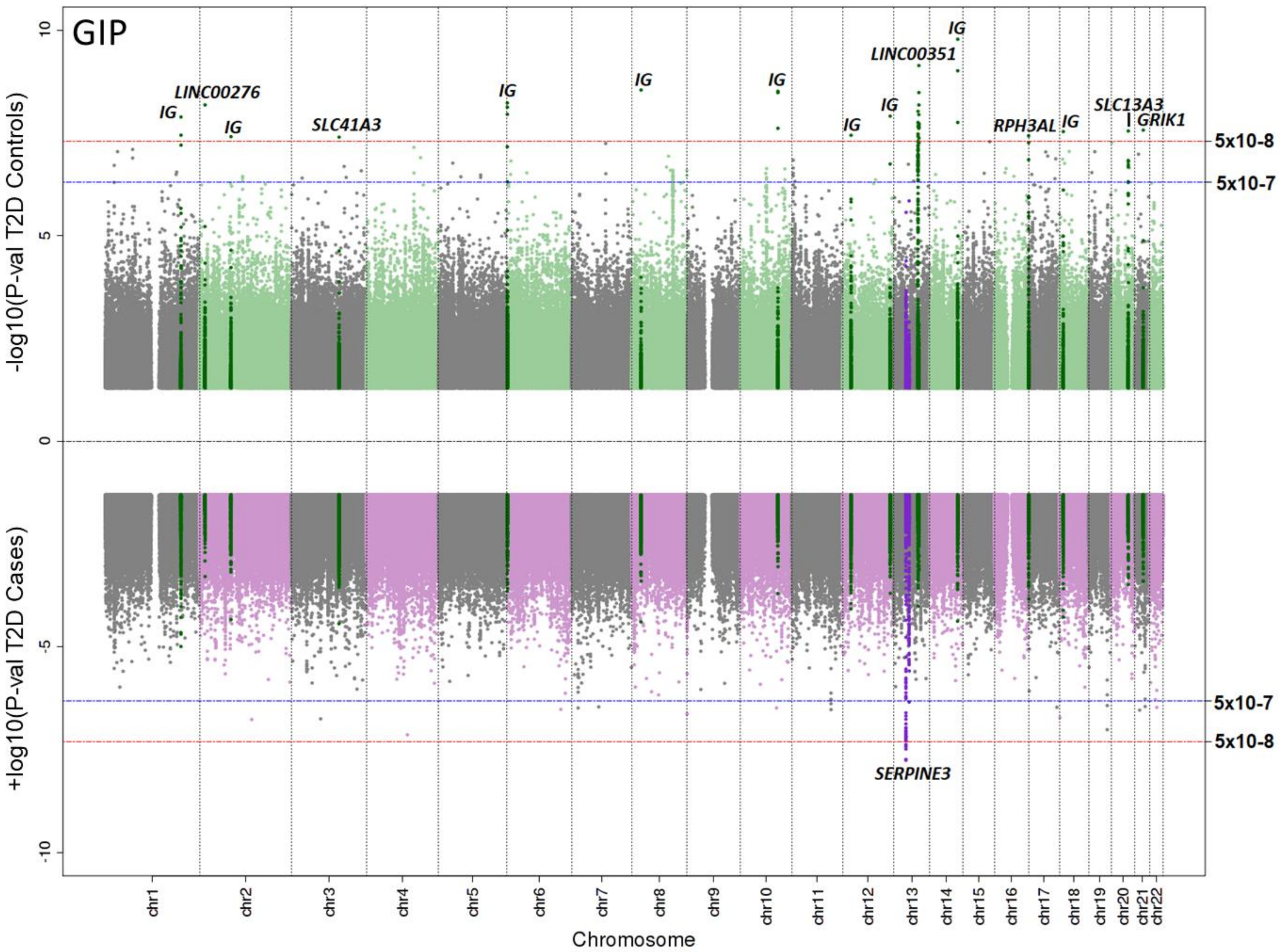
GIP
-log10(P-val T2D Controls)
+log10(P-val T2D Cases)
10
5
0
-5
-10
IG
LINC00276
IG
SLC41A3
IG
IG
IG
IG
IG
LINC00351
IG
RPH3AL
IG
SLC13A3
GRIK1
SERPINE3
5x10-8
5x10-7
5x10-7
5x10-8
chr1
chr2
chr3
chr4
chr5
chr6
chr7
chr8
chr9
chr10
chr11
chr12
chr13
chr14
chr15
chr16
chr17
chr18
chr19
chr20
chr21
chr22
Chromosome

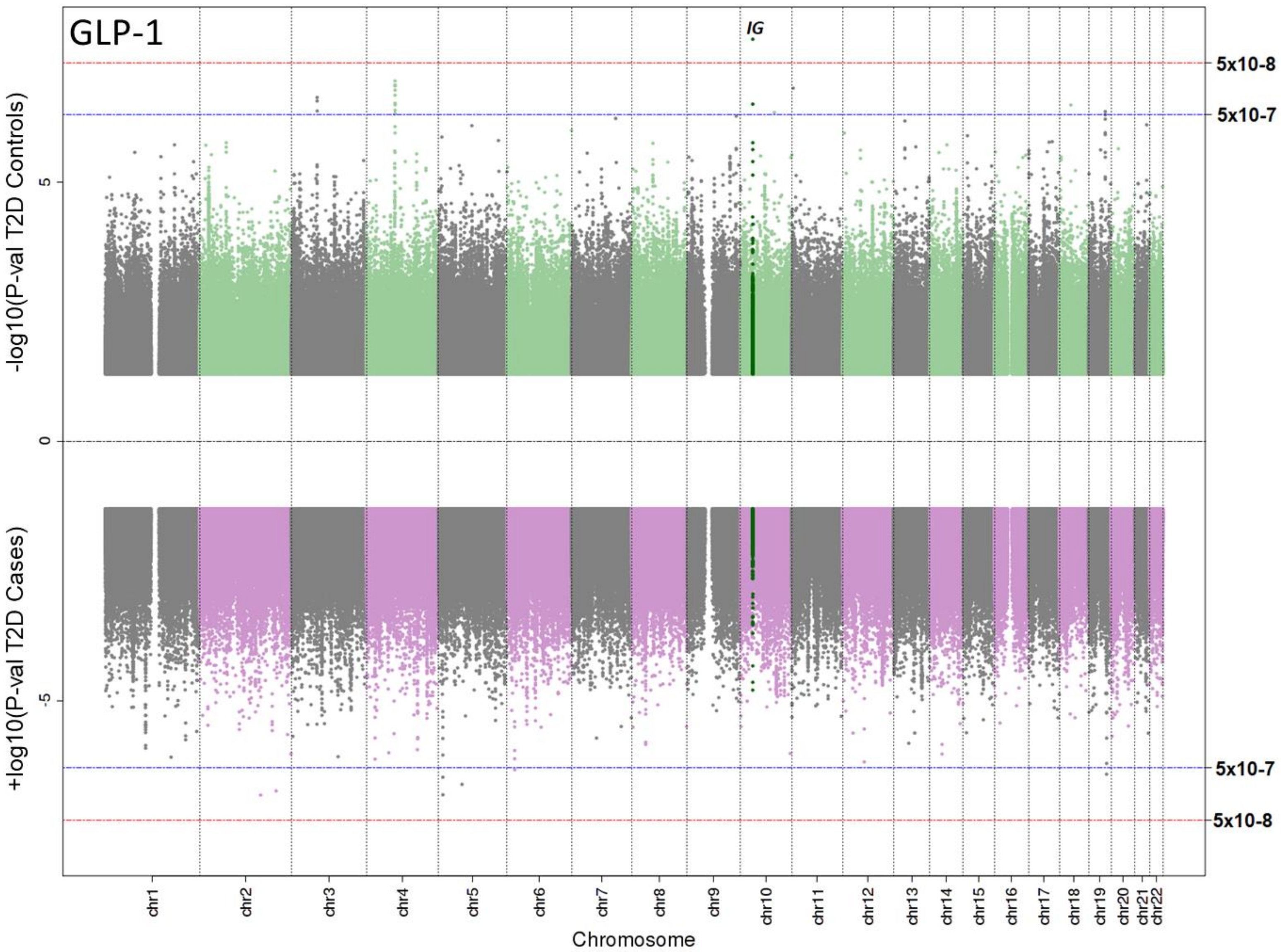
GLP-1
IG
5x10-8
5x10-7
5x10-7
5x10-8
-log10(P-val T2D Controls)
+log10(P-val T2D Cases)
5
0
-5
chr1
chr2
chr3
chr4
chr5
chr6
chr7
chr8
chr9
chr10
chr11
chr12
chr13
chr14
chr15
chr16
chr17
chr18
chr19
chr20
chr21
chr22
Chromosome

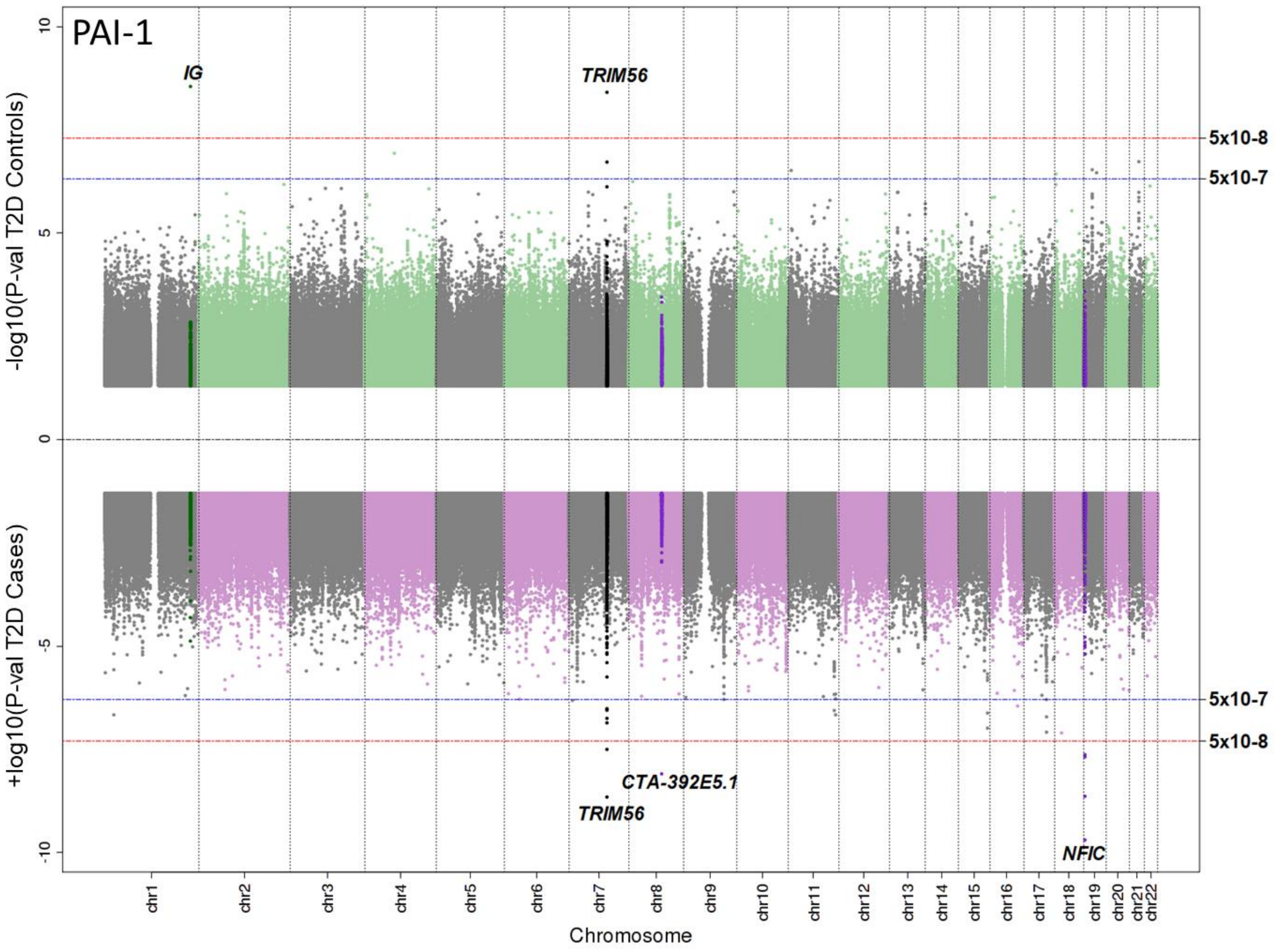
PAI-1
IG
TRIM56
CTA-392E5.1
TRIM56
NFIC
5x10-8
5x10-7
5x10-7
5x10-8
-log10(P-val T2D Controls)
+log10(P-val T2D Cases)
10
5
0
-5
-10
chr1
chr2
chr3
chr4
chr5
chr6
chr7
chr8
chr9
chr10
chr11
chr12
chr13
chr14
chr15
chr16
chr17
chr18
chr19
chr20
chr21
chr22
Chromosome

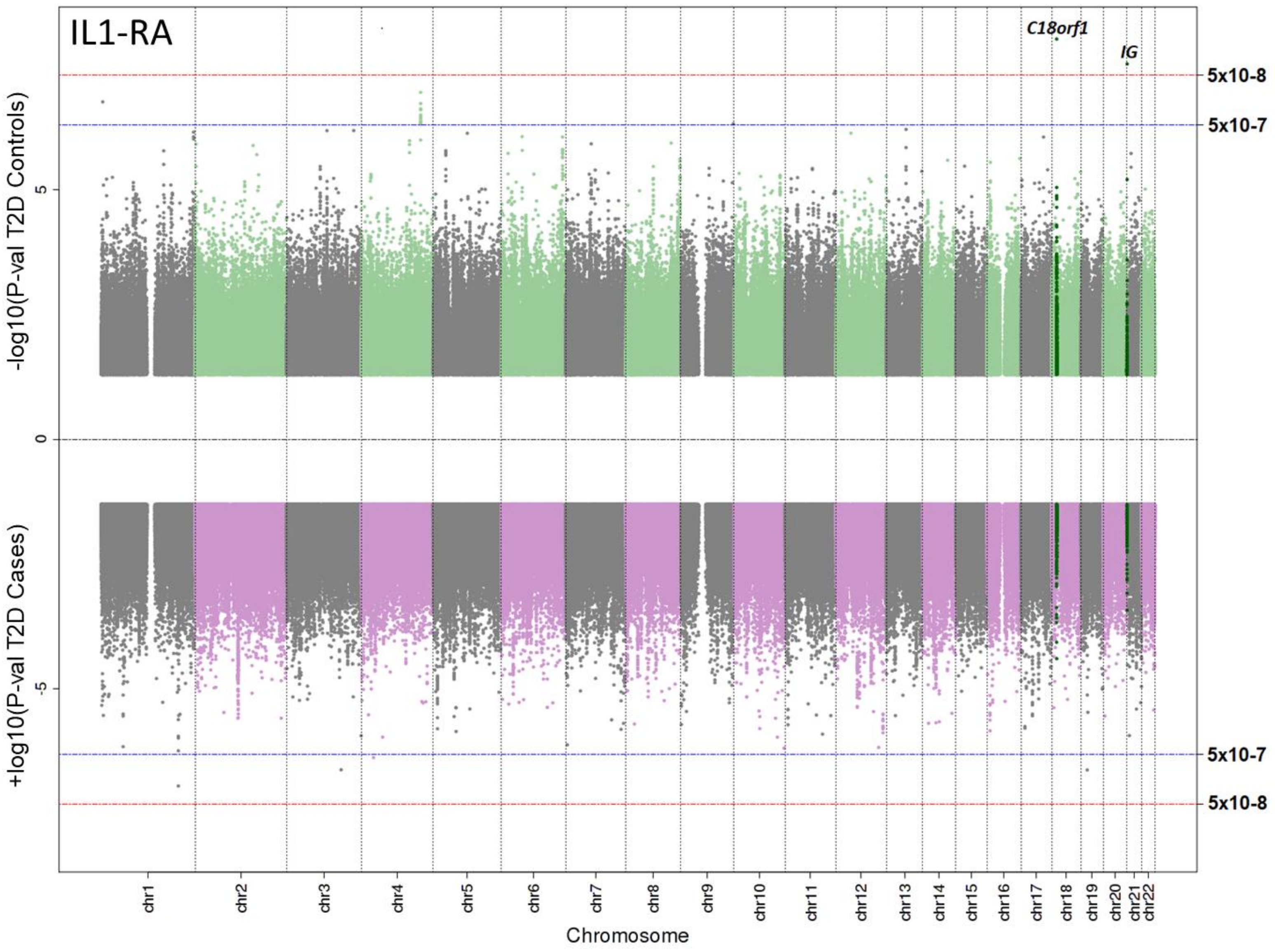

IL1-RA
C18orf1
IG
5x10-8
5x10-7
-log10(P-val T2D Controls)
+log10(P-val T2D Cases)
5
0
-5
chr1
chr2
chr3
chr4
chr5
chr6
chr7
chr8
chr9
chr10
chr11
chr12
chr13
chr14
chr15
chr16
chr17
chr18
chr19
chr20
chr21
chr22
Chromosome

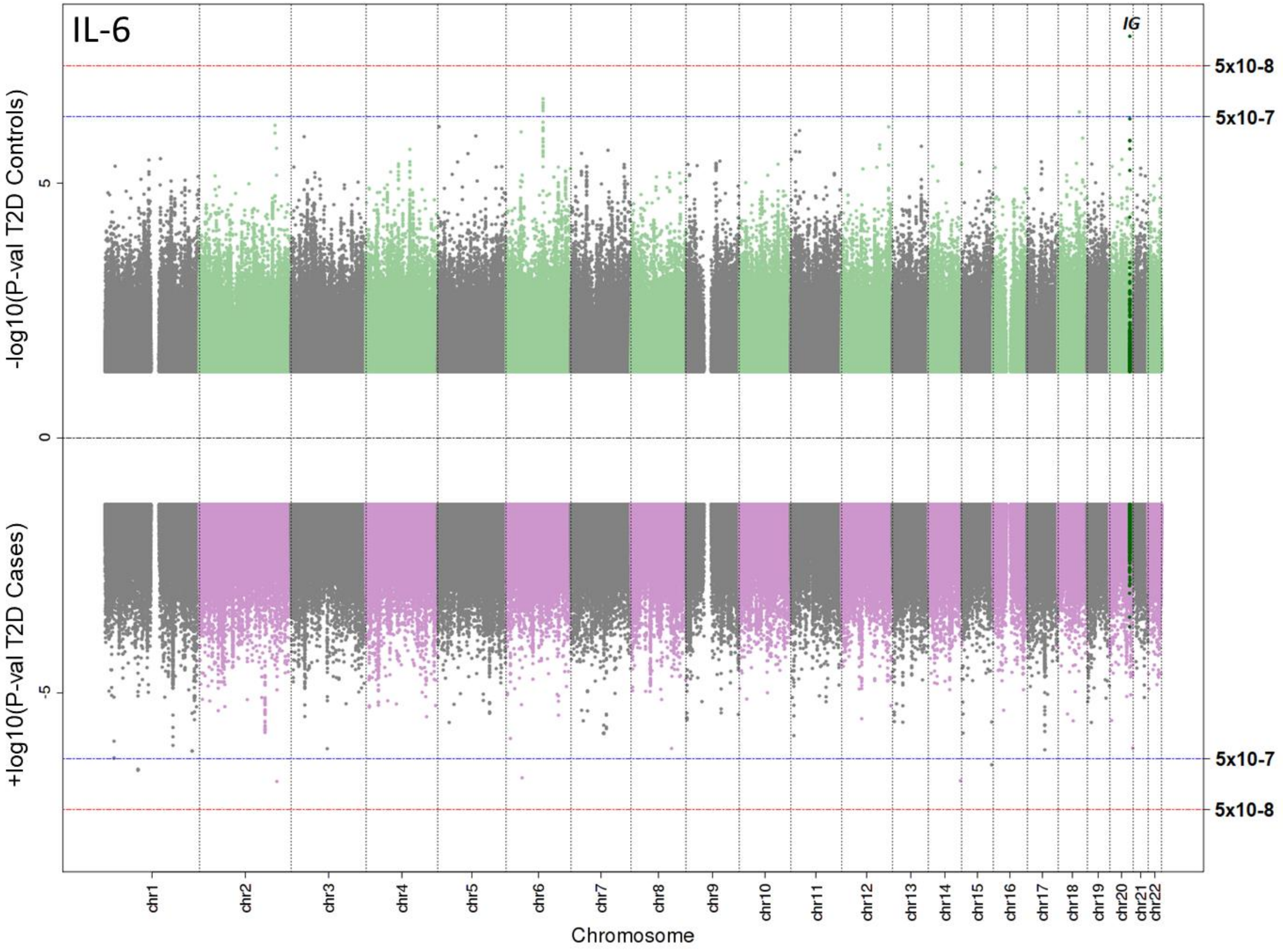

IL-6
IG
5x10-8
5x10-7
5x10-7
5x10-8
-log10(P-val T2D Controls)
+log10(P-val T2D Cases)
5
0
-5
chr1
chr2
chr3
chr4
chr5
chr6
chr7
chr8
chr9
chr10
chr11
chr12
chr13
chr14
chr15
chr16
chr17
chr18
chr19
chr20
chr21
chr22
Chromosome

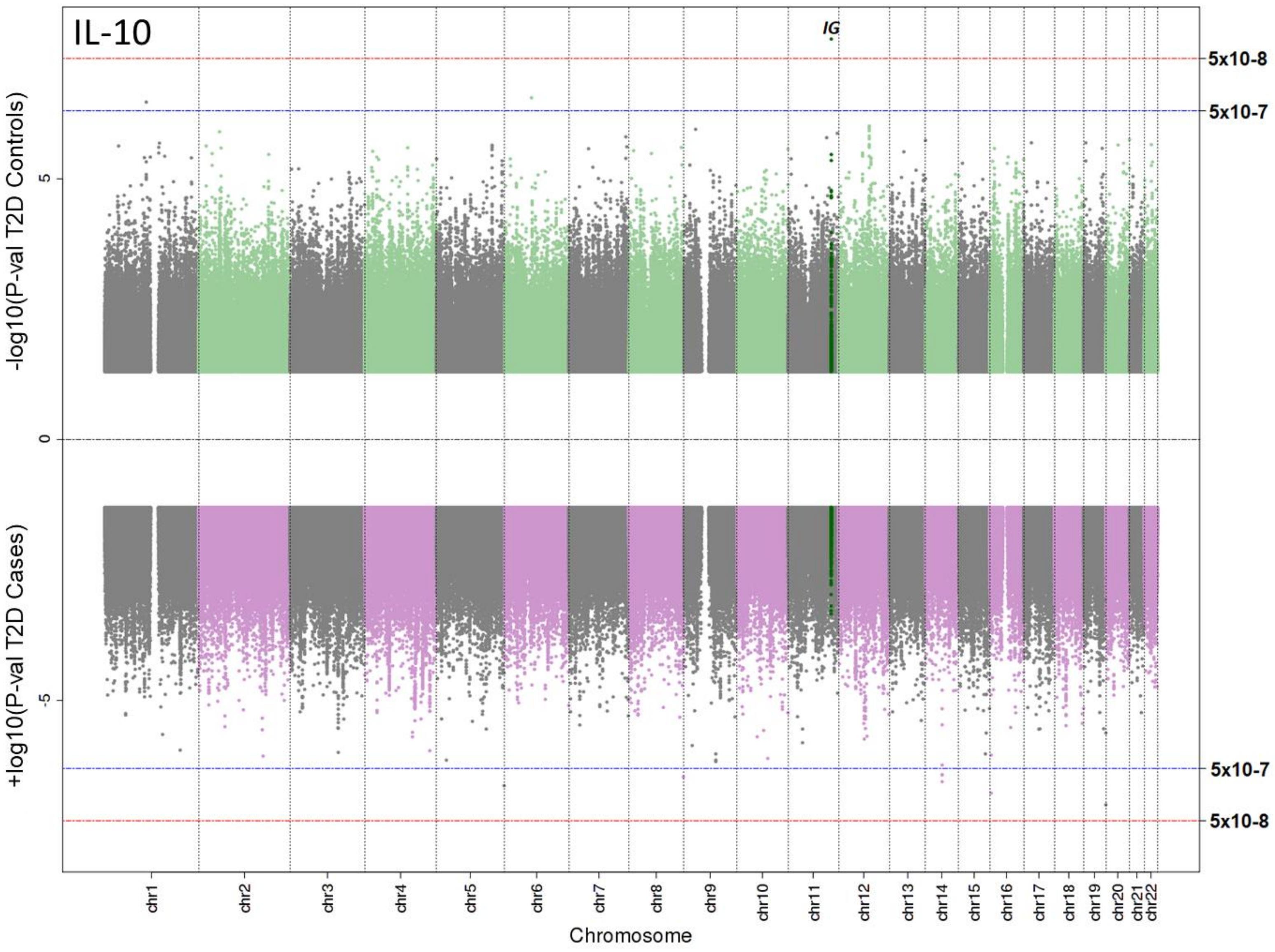
IL-10
IG
5x10-8
5x10-7
5x10-7
5x10-8
-log10(P-val T2D Controls)
+log10(P-val T2D Cases)
5
0
-5
chr1
chr2
chr3
chr4
chr5
chr6
chr7
chr8
chr9
chr10
chr11
chr12
chr13
chr14
chr15
chr16
chr17
chr18
chr19
chr20
chr21
chr22
Chromosome

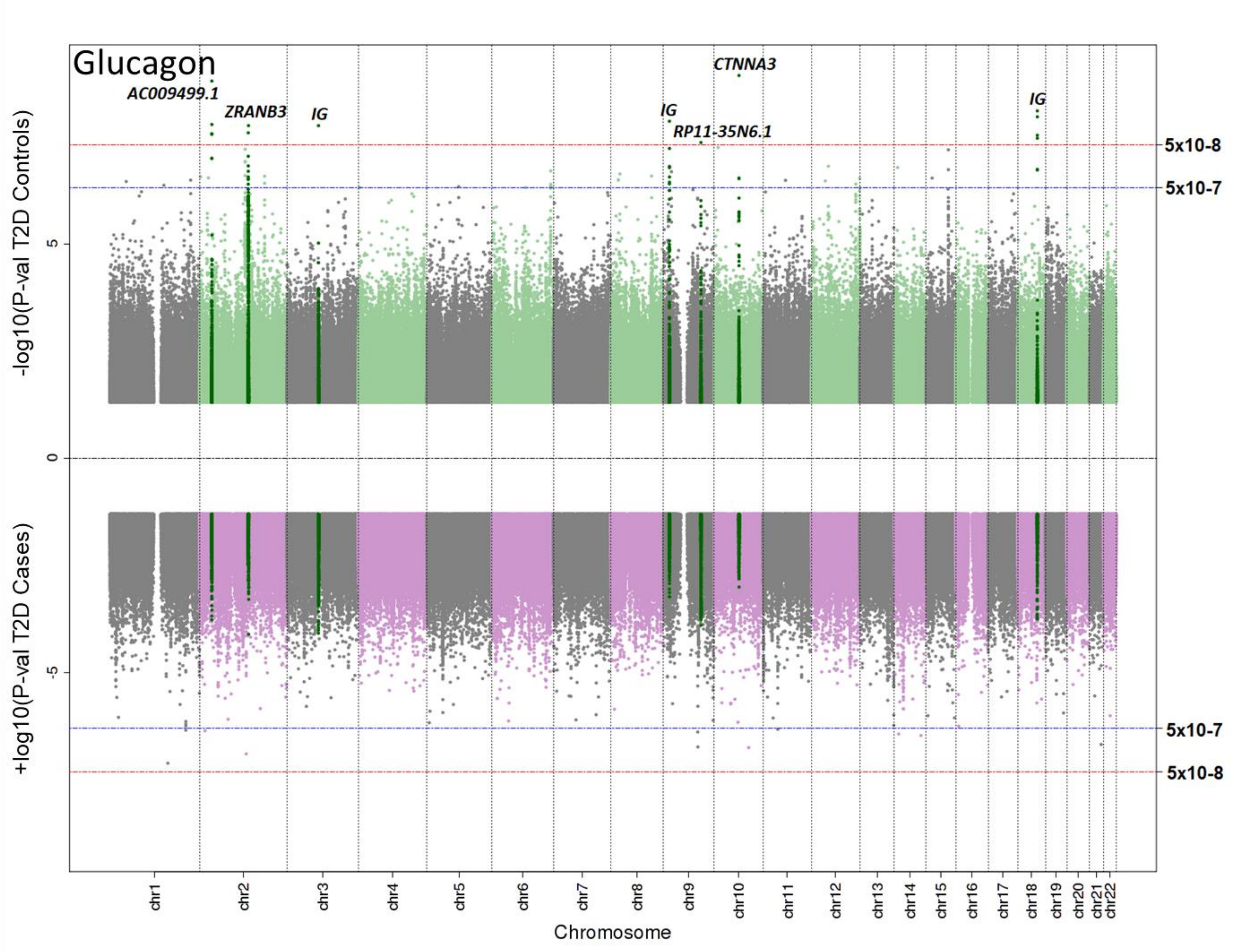

Glucagon
AC009499.1
ZRANB3
IG
IG
CTNNA3
RP11-35N6.1
IG
5x10-8
5x10-7
5x10-7
5x10-8
-log10(P-val T2D Controls)
+log10(P-val T2D Cases)
5
0
-5
chr1
chr2
chr3
chr4
chr5
chr6
chr7
chr8
chr9
chr10
chr11
chr12
chr13
chr14
chr15
chr16
chr17
chr18
chr19
chr20
chr21
chr22
Chromosome

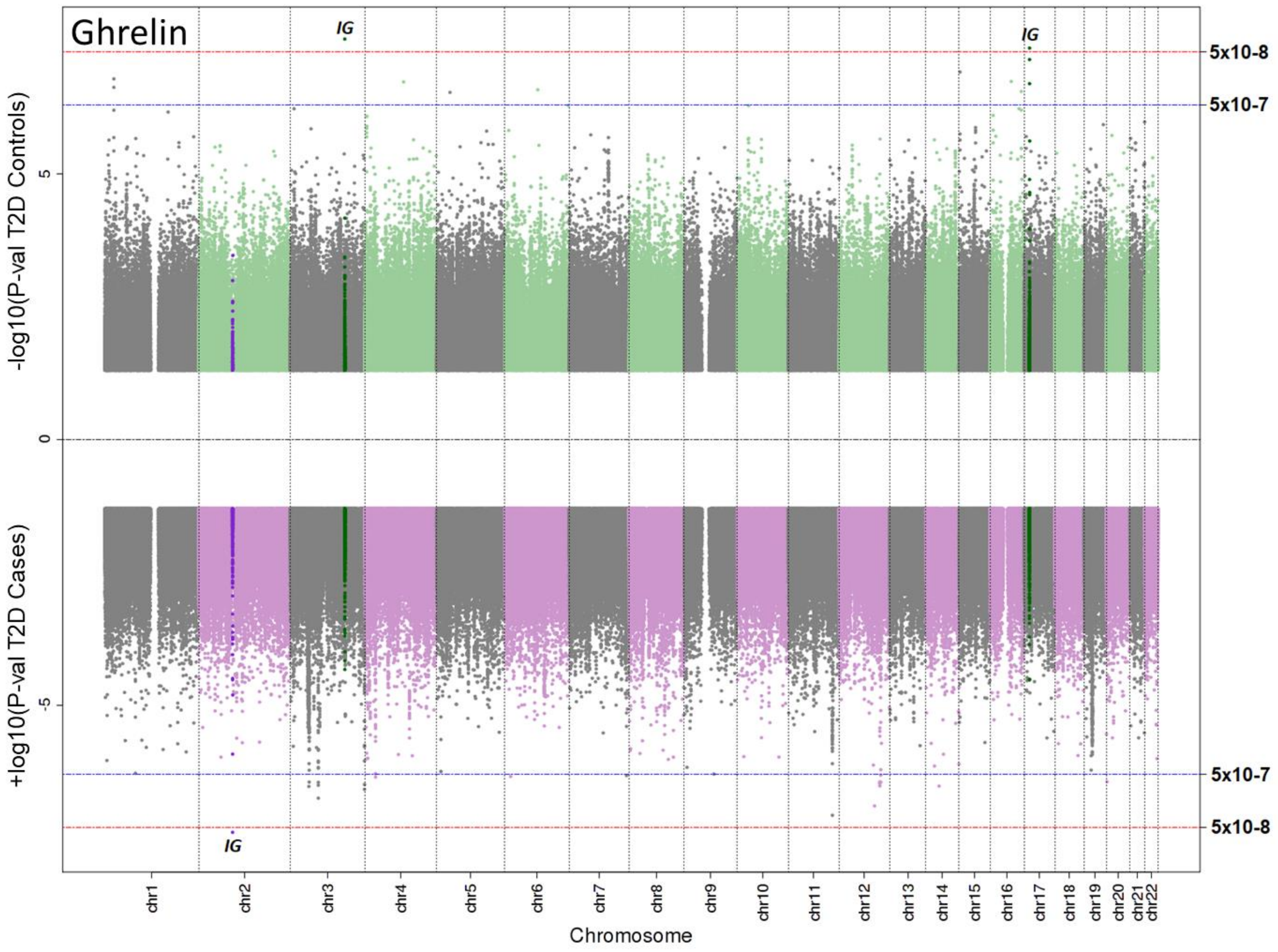

Ghrelin
IG
IG
IG
5x10-8
5x10-7
5x10-7
5x10-8
-log10(P-val T2D Controls)
+log10(P-val T2D Cases)
5
0
-5
chr1
chr2
chr3
chr4
chr5
chr6
chr7
chr8
chr9
chr10
chr11
chr12
chr13
chr14
chr15
chr16
chr17
chr18
chr19
chr20
chr21
chr22
Chromosome

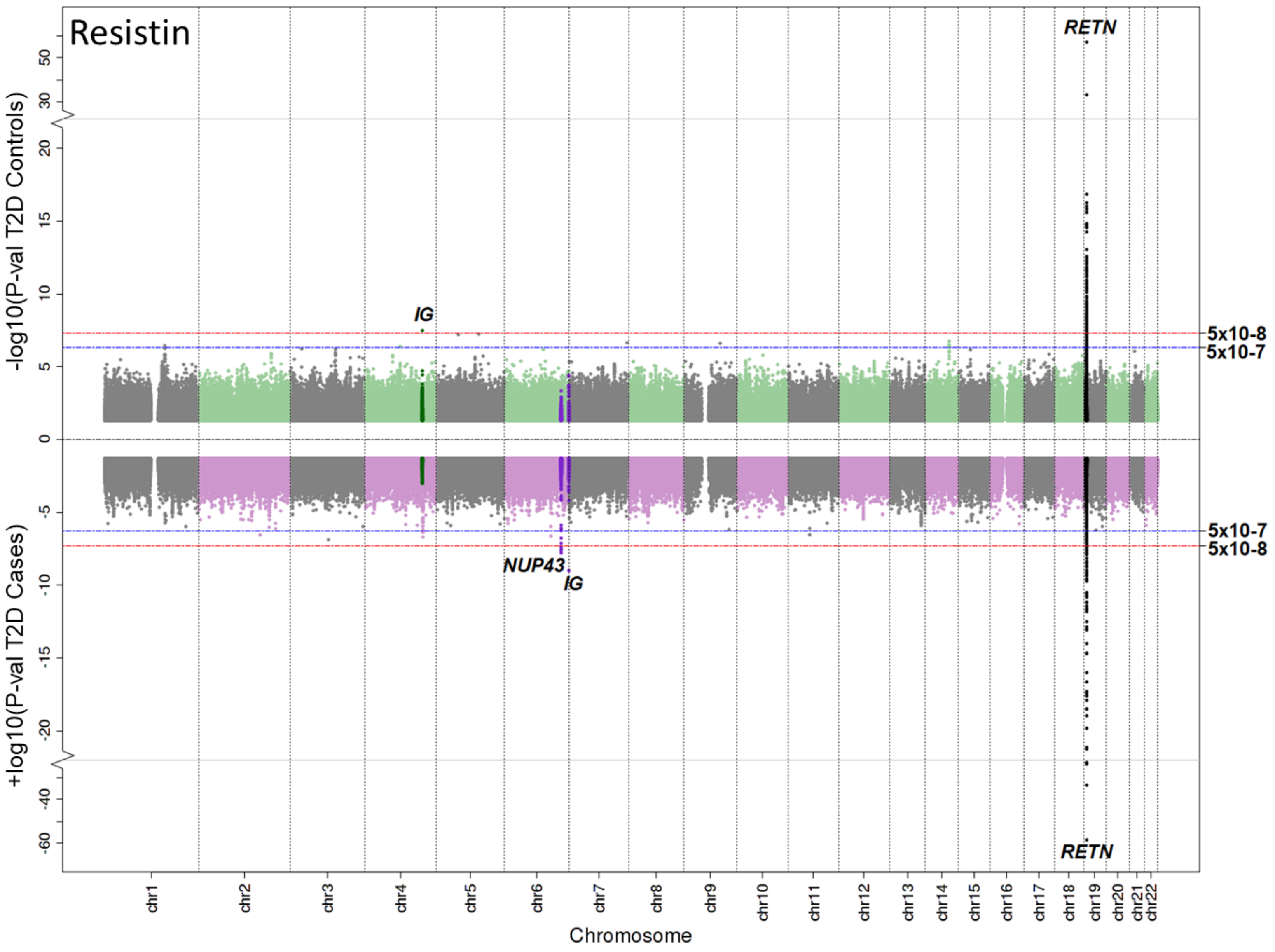
Resistin
RETN
IG
NUP43
IG
RETN
5x10-8
5x10-7
5x10-7
5x10-8
-log10(P-val T2D Controls)
+log10(P-val T2D Cases)
Chromosome
chr1
chr2
chr3
chr4
chr5
chr6
chr7
chr8
chr9
chr10
chr11
chr12
chr13
chr14
chr15
chr16
chr17
chr18
chr19
chr20
chr21
chr22

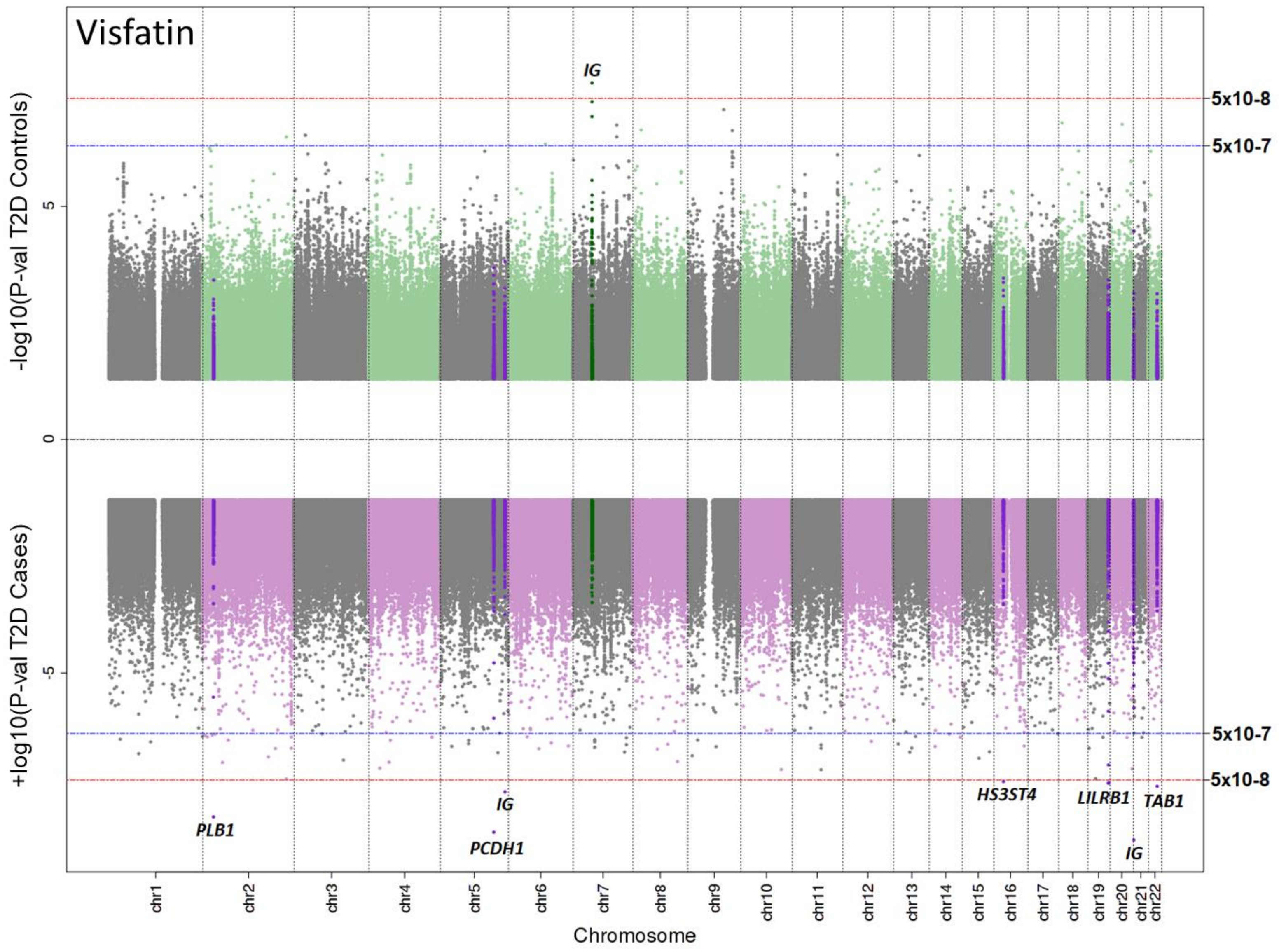
Visfatin
IG
5x10-8
5x10-7
-log10(P-val T2D Controls)
+log10(P-val T2D Cases)
5
0
-5
5x10-7
5x10-8
PLB1
IG
PCDH1
HS3ST4
LILRB1
TAB1
IG
chr1
chr2
chr3
chr4
chr5
chr6
chr7
chr8
chr9
chr10
chr11
chr12
chr13
chr14
chr15
chr16
chr17
chr18
chr19
chr20
chr21
chr22
Chromosome
